# Automated tracking of cell migration in phase contrast images with CellTraxx

**DOI:** 10.1038/s41598-023-50227-9

**Published:** 2023-12-27

**Authors:** Børge Holme, Birgitte Bjørnerud, Nina Marie Pedersen, Laura Rodriguez de la Ballina, Jørgen Wesche, Ellen Margrethe Haugsten

**Affiliations:** 1 SINTEF Industry, Forskningsveien 1, 0373 Oslo, Norway; 2 Department of Tumor Biology, Institute for Cancer Research, The Norwegian Radium Hospital, Oslo University Hospital, Montebello, 0379 Oslo, Norway; 3 Centre for Cancer Cell Reprogramming, Institute of Clinical Medicine, Faculty of Medicine, University of Oslo, Montebello, 0379 Oslo, Norway; 4 Department of Molecular Cell Biology, Institute for Cancer Research, The Norwegian Radium Hospital, Oslo University Hospital, Montebello, 0379 Oslo, Norway; 5 Department of Nursing, Health and Laboratory Science, Faculty of Health, Welfare and Organisation, Østfold University College, PB 700, NO-1757 Halden, Norway; 6 Department of Molecular Medicine, Institute of Basic Medical Sciences, University of Oslo, 0372 Oslo, Norway

**Keywords:** Cell migration, Software

## Abstract

The ability of cells to move and migrate is required during development, but also in the adult in processes such as wound healing and immune responses. In addition, cancer cells exploit the cells’ ability to migrate and invade to spread into nearby tissue and eventually metastasize. The majority of cancer deaths are caused by metastasis and the process of cell migration is therefore intensively studied. A common way to study cell migration is to observe cells through an optical microscope and record their movements over time. However, segmenting and tracking moving cells in phase contrast time-lapse video sequences is a challenging task. Several tools to track the velocity of migrating cells have been developed. Unfortunately, most of the automated tools are made for fluorescence images even though unlabelled cells are often preferred to avoid phototoxicity. Consequently, researchers are constrained with laborious manual tracking tools using ImageJ or similar software. We have therefore developed a freely available, user-friendly, automated tracking tool called *CellTraxx*. This software makes it easy to measure the velocity and directness of migrating cells in phase contrast images. Here, we demonstrate that our tool efficiently recognizes and tracks unlabelled cells of different morphologies and sizes (HeLa, RPE1, MDA-MB-231, HT1080, U2OS, PC-3) in several types of cell migration assays (random migration, wound healing and cells embedded in collagen). We also provide a detailed protocol and download instructions for CellTraxx.

## Introduction

The ability of cells to migrate is fundamental in many physiological processes, including immune responses, embryonic development, angiogenesis, regeneration, and wound healing. In addition, cancer cells depend on their ability to migrate in order to spread and metastasize. Cell migration is crucial at almost all steps of the metastatic process, including breaching the basement membrane, escaping from the primary tumour, movement to blood and lymphatic vessels, intravasation, extravasation, and migration into distant organs^1^. Therefore, cell migration has been under extensive investigation. A common, low-cost way to study cell migration is time-lapse imaging of cells using optical microscopy, and analysis of their movements over time. In these experiments cells are either seeded out sparsely to allow individual cells to migrate around the dish (random single-cell migration assays) or densely with an open area without cells, to allow migration into the open area (wound healing assays). The latter allows cells to migrate in sheets or strands into the open area and is used to study collective cell migration. A major challenge in high and semi-high throughput cell migration screening is the quantification of cell migration. In common approaches, such as the wound healing assays, the area occupied by the migrating cells over time is estimated to reflect the migration properties of the cells. Such an approach might lead to false results if, for example, cell proliferation or cell size is not constant during the experiment. Therefore, tracking cell movements to determine parameters such as velocity, directness, accumulated distance, and Euclidian distance is preferable.

In many laboratories, ours included, tracking of unlabelled migrating cells is often performed manually using the Manual Tracking tool plugin in ImageJ/Fiji^2,3^. Although this manual tracking tool works well, tracking cells in this manner is subjective and extremely time-consuming, which may lead to poor reproducibility. It requires clicking on each cell to be tracked in each image throughout the whole image series. In an experiment with two conditions and with imaging every 10 min for 10 h (a total of 60 images) tracking 50 cells per condition, adds up to 6 000 clicks (60 images × 50 cells × 2 conditions). Repeating the experiment 3 times requires 18 000 clicks, which takes several hours of manual work. Including more conditions, longer imaging times, or high throughput experiments makes manual tracking unmanageable. To overcome this, several computer-based tools to track migrating cells have been developed^4,5^. Several recent papers summarize and compare such programs^4–8^. Segmentation of cells and accurately defining their centre points, are the main challenges of automated cell tracking. To ease segmentation most of the automated tools are based on fluorescently labelled cells^5^. Fluorescence can be potentially risky since the added probes can alter the properties and behaviour of the cells. In addition, phototoxicity is often observed when cells are exposed to fluorescent light over time^9^. Even though segmentation and tracking of cells in phase contrast images is difficult, there are some options available. The different options can be divided into three: (1) conventional tracking tools, such as iTrack4U^10^ and AMIT^11,12^, (2) tracking platforms which allow external segmentation algorithms such as TrackMate^13,14^, CellProfiler^15,16^, and Lineage Mapper^17^, (3) segmentation and tracking tools applying deep learning algorithms such as Usiigaci^18^ and EmbedTrack^19^. There are in general few conventional tracking tools for phase contrast images. iTrack4U^10^ is a user-friendly ImageJ-based tool, suitable for smaller datasets as it requires manual selection of the cells to be tracked. AMIT is able to track immune cells, macrophages and fungal cells in phase contrast images, but requires experience with the C ++ language^11,12^. Combined with robust segmentation algorithms TrackMate, CellProfiler and Lineage Mapper are powerful tracking tools^13–17^. The recent successes of deep learning-based cell segmentation, such as Cellpose^20^, provide excellent segmentation of cell outlines in phase contrast images. TrackMate is a user-friendly tracking plugin for ImageJ/Fiji. Cellpose can now be incorporated into TrackMate (Cellpose + TrackMate, later abbreviated to CP + TM) for a more user-friendly experience^13^. However, this still requires the local installation of Cellpose, which involves setting a dedicated Python environment that complies with all package dependencies, and mapping of the Cellpose installation and local models within the TrackMate plugin. CellProfiler is a powerful tool for image analysis that allows for object segmentation and tracking. Lineage Mapper was originally developed in MatLab but has been converted into a ImageJ/Fiji plugin^17^. Another tracking tool for stain-free phase contrast images based on deep learning algorithms for segmentation is Usiigaci^18^. The Usiigaci segmentation module is based on the Mask-R-CNN allowing tracking of cell position as well as cell morphology over time. Cell tracking of the segmented images can be done in ImageJ, or other tracker software such as Lineage Mapper or Usiigaci tracker. EmbedTrack is a tracker that applies deep learning algorithms for both cell segmentation and cell tracking^19^. Clearly, with artificial intelligence and deep learning-based algorithms for segmentation and tracking, the tools emerging in recent years are very powerful, able to accurately segment and track different cells from different image types. However, most of these tools have a high user threshold for pure biologists. Experience in programming languages that are unfamiliar to many biologists as well as complicated software installation including compilers and language packages are often required. In many cases, several tools and/or software packages are needed. With deep learning algorithms, time-consuming training of datasets might be necessary to obtain good segmentation. Therefore, many biologists still end up tracking their cells manually.

We have developed a fully automated, easy-to-use and freely available tracking tool, called *CellTraxx*, which allows label-free tracking of cells in phase contrast images. CellTraxx was developed to measure the migration velocity in hundreds or thousands of videos with several hundred cells per image. The user determines the initial settings based on the first video, then CellTraxx analyses all videos with no need for user interference or post processing of tracks. By analysing large numbers of cell movements, the overall cell velocity distribution can be accurately measured, despite some imperfections in trajectories of individual cells. CellTraxx does not find the exact outline of the cell’s periphery, but instead determines the nuclei’s centroid based on their darker shades of grey in the phase contrast images.

In brief, CellTraxx reads an image series, corrects poor image alignment, segments and identifies individual cell nuclei, and matches cells with the previous image. CellTraxx was originally written to batch process several videos, while running in a command window. In addition, an optional, user-friendly, window-based interface more familiar to most users is provided through a macro in ImageJ. CellTraxx can thus be downloaded freely as an exe file and an ImageJ macro, plus six additional ancillary files. The user-friendly interface in ImageJ provides a dialog window to set the most important parameters, as well as an interactive tuning window to change and evaluate the key settings. We have chosen to use phase contrast image series from the Incucyte S3 microscope (Sartorius) since this is a user-friendly, live-cell, automated, high throughput microscope utilized in many laboratories. However, since consecutive images from the Incucyte microscope are not always well aligned, CellTraxx can automatically align images to ensure proper tracking. As output, CellTraxx generates a new image series with valid tracks superimposed on the (aligned) phase contrast images, several plots (cell trajectory plots, velocity over time and others) in addition to several CSV (Comma-Separated Values) files with quantifications and statistics for each image series. Further, CellTraxx creates several summary files with one row of data for each image series (video) that was analysed simultaneously. Users can easily find most of the results they need in one of these summary files. This is convenient when analysing hundreds or thousands of videos in one run of the program.

We here demonstrate that CellTraxx is capable of tracking a wide range of cells with different morphologies, sizes, and speeds (HeLa, RPE1, MDA-MB-231, HT1080, U2OS, PC-3) in both random single-cell migration assays and in wound healing experiments. In addition, CellTraxx is able to recognize and track cells embedded in matrices such as collagen. We also show that the results from CellTraxx compare well with results obtained by our former standard tracking method of using the manual tracking plugin in ImageJ as well as with tracking by TrackMate combined with cell segmentation by Cellpose. In addition, we demonstrate that CellTraxx can be used for cell counting for proliferation experiments. We provide a detailed protocol as well as download instructions for CellTraxx. Since CellTraxx can process large numbers of long image series (videos), we propose that the software can be used for automated high/semi-high-throughput screening of cell migration.

## Results

### How CellTraxx works

**Figure 1:**
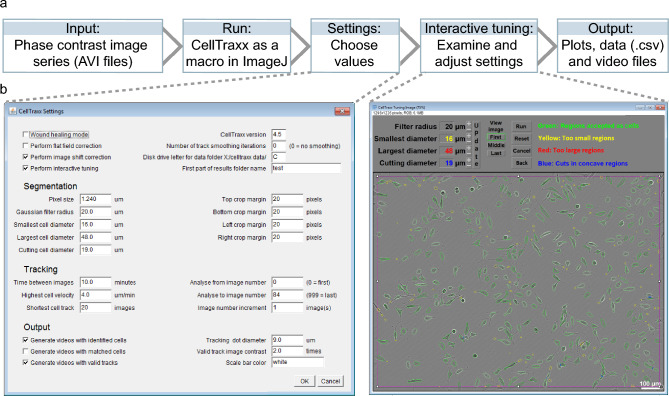
Overview of CellTraxx. (**a**) Outline of CellTraxx workflow. (**b**) CellTraxx graphical user interfaces, the settings window (to the left) and the interactive tuning-window (to the right).

CellTraxx and examples videos can be downloaded from GitHub (https://github.com/borge-holme/celltraxx_download). A detailed protocol including troubleshooting for CellTraxx is found there as well as in Supplementary File S1. The program reads phase contrast image series (AVI videos), tracks cells from one image to the next and calculates migration measures such as velocity, directness, accumulated distance, and Euclidian distance. The user will have to export the phase contrast image series from an Incucyte microscope (or other microscope with similar phase contrast images) as AVI files. Then, start CellTraxx as a macro in ImageJ, set the desired parameters for tracking of the particular cell line, inspect and optimize the cell selection settings in the tuning window, run the program and finally read and evaluate the output data (Fig. 1).

CellTraxx was developed for phase contrast image series from Incucyte S3, applying the 10X objective. In these image series, the cells have a fairly good contrast compared to, for example, bright field imaging. CellTraxx can process from one to thousands of image series in one run since intermediate images are stored on the hard drive and not in the computer’s active memory (RAM). Since, CellTraxx is written in C, it is also relatively fast. We have successfully used CellTraxx to analyse a cell migration drug screen with approximately 2000 videos in which more than 500,000 cells were tracked (data will be published elsewhere).

To optimize tracking of Incucyte image series, we have generated an optional function in CellTraxx to correct poorly aligned images. Videos generated by Incucyte are often not perfectly aligned in the *X* or *Y* direction despite the use of special imaging plates (Incucyte® ImageLock 96-well plates from Sartorius) that aid revisiting and imaging at the same (*X,Y*) position in the well. To ensure accurate alignment, CellTraxx finds how far the next image must be shifted laterally to look most similar to the previous image and then performs the shift in fractional pixels. For a detailed description on how CellTraxx corrects image shifts, see Supplementary File S2. Figure S1a shows a plot of velocity versus time for HeLa cells in an image series analysed with and without image shift correction. The largest spike in the purple curve appears where the next image has a shift of 3.1 µm relative to the previous image. This gives an artificial increase of 0.11 µm/min in the average velocity of the non-corrected image series at this point in time. (The velocity increase is not as large as 3.1 µm / 10 min = 0.31 µm/min since not all velocity vectors are changed by the same amount for any given image shift). Figure S1b,c show how the parameter called “Velocity sum per cell” (the common movement of all cells from one image to the next) can reveal image misalignments in videos run without image shift correction. The velocity sum per cell strongly correlates with the difference in velocity obtained without and with image shift correction (Fig. S1b). Normally the velocity sum per cell should be below 0.1 µm/min (Fig. S1c). However, if the video has visible shifts and/or the velocity sum per cell has peaks well above the 0.1 µm/min threshold, the image series should be analysed again with image shift correction turned on. For image series generated by Incucyte, we always recommend to keep image shift correction turned on. Running CellTraxx with image shift correction will increase the analysis time by about 20%.

Next, CellTraxx segments the cells. This is performed by first running a wide Gaussian smoothing filter over the image to smear out the dark spots in the cell nuclei. Then, CellTraxx analyses the grey levels in the image and determines the grey level limit for segmentation as the darkest pixels in the cell-free background. All pixels darker than this limit are marked as belonging to candidate cells. Wherever two or more cells are in close proximity, the segmentation may not always manage to separate them as individual cells. Therefore, a special routine has been added to CellTraxx which cuts connected regions at convex points on the periphery. We developed a method which analyses the local curvature along the edge of the regions and draws a separation line between nearby, convex points that have a small radius of curvature. For a more detailed description see Supplementary File S2. This cutting routine often manages to separate two or more connected cells. CellTraxx will then identify and number the candidate cells while scrapping regions that are smaller or larger than the user-defined limits.

After segmentation and identification of cells, CellTraxx calculates the geometric “centre of mass” (centroid) of all pixels which constitute an identified cell. Then, CellTraxx matches the identified cells in one image with the corresponding cells in the previous image, using a nearest neighbour approach similar to Yang et al.^21^. See more details in Supplementary File S2. The cells that have moved the least are matched first, since they are considered the most certain matches. Then the matching continues until the longest allowed cell shift is reached. If a cell is not recognized in the previous image, a simple track-repair algorithm in CellTraxx searches for the closest unmatched cell in the image two time-steps earlier. If an acceptable candidate cell is found, the track is repaired by placing the missing cell in the previous image midway between the two identified cells. If the cell is not matched by examining two images back, then CellTraxx stores the cell location for the possibility of beginning a new track if a matching cell is found in the next image. A cell might not be recognized in the previous images due to, for example, changes in contrast or cell morphology leading to loss of the dark spots in the nucleus, or if the cell is hidden behind or too close to other cells. Some cells may move outside the analysis region, but then come back later. Therefore, some tracks are ended prematurely and restarted, especially if cells are confluent. Thus, CellTraxx generates a somewhat higher number of tracks than the actual number of cells identified in the first image. This is partly due to abruptions such as those mentioned above, but also due to cell division. As a cell divides, CellTraxx will normally continue to track the nearest daughter cell while generating a new track for the other (see Movie 1). However, sometimes the program instead starts two new tracks. If, after running an analysis, the number of tracks strongly exceeds the number of cells recognized in the first and last image, the settings could probably be improved. For a detailed troubleshooting list se Supplementary File S1, CellTraxx *User Manual*.

The “CellTraxx Settings” window in the ImageJ interface (Fig. 1b, left image) presents the user with default parameters from the previous run. Here, general settings as well as settings for segmentation, tracking, and output, are selected. The interactive tuning tool (Fig. 1b, right image) allows the user to examine the effect of and fine-tune the most relevant selection parameters. A more detailed description of the settings can be found in the CellTraxx *User Manual* (Supplementary File S1).

**Figure 2:**
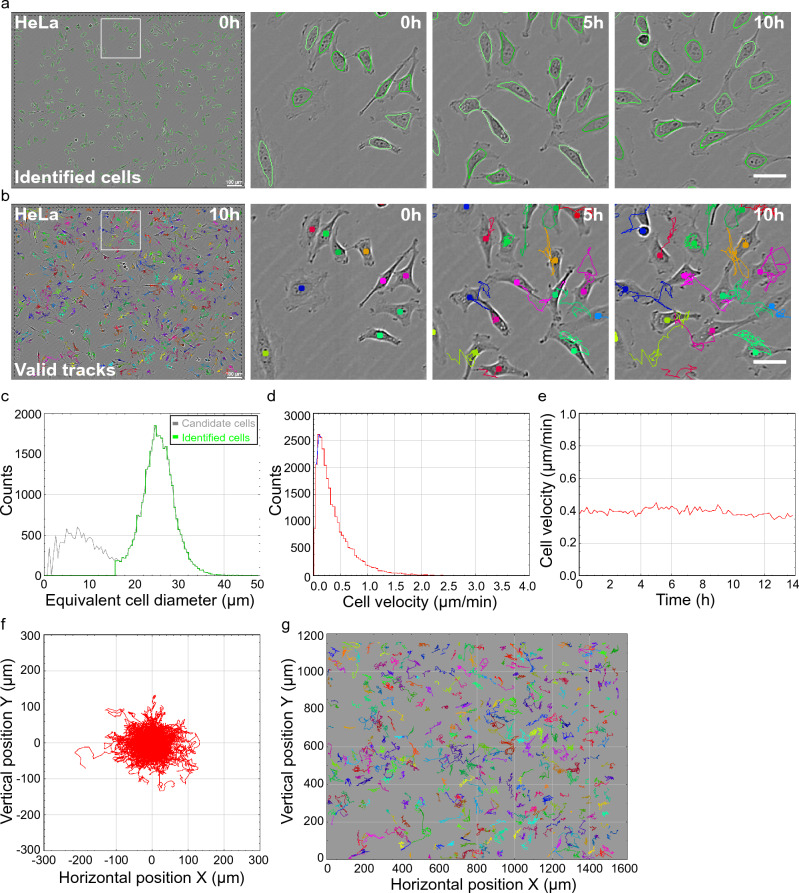
Example of tracking and output files automatically generated by CellTraxx. HeLa cells were imaged every 10 min in Incucyte S3 for 14 h. The image series was then analysed by CellTraxx. An overview of the used settings can be found in Supplementary file S3. (**a**,**b**) Images of HeLa cells showing identified cells outlined in nuances of green (**a**) and valid tracks in different colours (**b**). The whole image is shown to the left while enlarged areas from different time points are presented to the right. Scale bar 100 µm, enlarged images scale bar 50 µm. (**c**) Size distribution curves with number of cells plotted versus the equivalent circular cell diameter for all candidate cells (grey) and the identified cells (green). Note that the cell diameter refers to the equivalent diameter of the region *outlined* by CellTraxx, which is generally smaller than the outer diameter of the cell. Candidate cells are all objects recognized by CellTraxx while identified cells are the recognized objects that are accepted as cells (based on size). (**d**) The velocity distribution is shown in a velocity histogram (red) with a fitted parabola (blue). (**e**) A plot representing the mean velocity over time. (**f**) A plot showing the cell trajectories plotted from a common origin. (**g**) A plot showing the cell trajectories located as in the images.

After running CellTraxx, several output files are generated, including AVI videos, bitmap images, numerical data as CSV files, and summary plots generated from selected CSV files. Detailed descriptions of the different output files can be found in the CellTraxx *User Manual* provided in Supplementary File S1. Some examples are also shown in Fig. 2.

### CellTraxx execution and output

To illustrate how CellTraxx works, we used a phase contrast image series generated by Incucyte S3 of sparsely seeded, untreated HeLa cells. CellTraxx and the ImageJ macro were installed as described in Supplementary File S1. Next, the parameters were adjusted using the tuning window (see Fig. 1a,b and Supplementary File S1). CellTraxx was then run to analyse the velocities of the HeLa cells (for full list of settings used, see Supplementary File S3). As demonstrated in Fig. 2a, CellTraxx recognizes most cells in the images, indicated by ovals in different shades of green in the image series output file named “Identified cells” (Fig. 2a and Movie 2). In the image series “Valid tracks” each cell that is part of a valid track (defined by settings such as “Shortest cell track”) is represented by a coloured dot (Fig. 2b and Movie 3). As the time-laps progresses, we can observe trajectories (tracks) behind the cells, indicated by a coloured line. The tracks show the path that the cells have migrated (Fig. 2b and Movie 3) from image to image.

To ease the analysis of the data generated by CellTraxx, a handful of useful plots are automatically produced. First, a histogram showing the size distributions of candidate cells (grey) and accepted/identified cells (green) (Fig. 2c). This can help the user to understand whether the size exclusion criteria are reasonable or not. Note that cell size refers to the equivalent diameter of the region outlined by CellTraxx, which is generally smaller than the actual outer diameter of the cell. Second, the velocity distributions are shown in a velocity histogram curve along with a fitted parabola (Fig. 2d). The velocity distribution curve can aid in determining the “Highest cell velocity” parameter for a cell type under given conditions. Another useful plot illustrates the velocity over time (Fig. 2e). The actual cell trajectories can be examined either plotted from a common origin (Fig. 2f) or with starting points as in the video (Fig. 2g). When plotted from a common origin, the tracks are shown in red (as in Fig. 2f), or drawn in colours corresponding to the valid tracks in the image series (not shown). The colour coded plot includes track numbers for the longest tracks. Similarly, for tracks plotted as displayed in the video, there are two versions of the plot, with (not shown) or without track numbers (Fig. 2g).

CellTraxx further generates numerical data in the form of several different CSV files. These contain common measures such as average velocity, accumulated distance, directness, forward migration index, Euclidian distance, and Euclidian velocity. Most of these standard cell migration measures are summarized in an overview file (*_Overall_summary.csv*). If several image series are run together, the overview file includes one row of these standard measures for each video. The detailed numbers behind all these measurements are found in separate files for each image series. The different files and their content are described more closely in Supplementary File S1.

### Comparing CellTraxx to manual tracking and Cellpose + TrackMate

**Figure 3:**
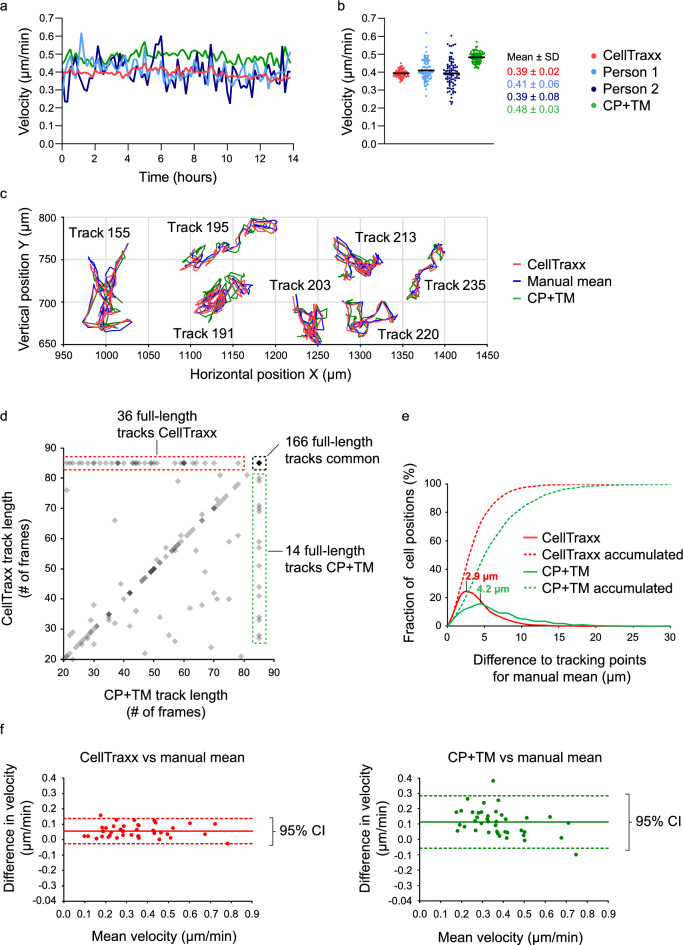
A comparison of CellTraxx with manual tracking and Cellpose + TrackMate (CP + TM). The same image series of migrating HeLa cells presented in Fig. 2 were also analysed by manual tracking and by CP + TM. (**a**) A comparison of mean velocity over time estimated from tracking by CellTraxx (red), two different persons (light and dark blue) and CP + TM (green). For person 1 and 2, 41 and 42 tracks are included (respectively) while for CellTraxx, a total of 442 tracks are included, and 479 tracks for CP + TM. (**b**) A comparison of the mean velocities from image to image tracked by CellTraxx (red), two different persons (light and dark blue) and CP + TM (green). Each dot represents the mean velocity from one time point to the next. The black line shows the overall mean velocity. (**c**) Seven different single cell trajectories tracked by CellTraxx (red), the mean of three different persons (blue) and CP + TM (green). The tracks are numbered with their track number from the CellTraxx analysis. Note that the analyses for manual tracking and CP + TM were also run on image shift corrected videos. (**d**) A comparison of the track length of the 316 matched trajectories from CellTraxx and CP + TM. A darker grey colour indicates overlapping points. The total number of frames in the video is 85. (**e**) A distribution plot showing the distances between track points for CellTraxx (red) and CP + TM (green) versus the mean of manual tracking of three different persons. Dashed lines represent the accumulated distribution. (**f**) Bland–Altman plots (difference plots) which compare the mean velocity for each of the 40 full-length trajectories that were successfully tracked by CellTraxx, CP + TM and the three persons.

We wanted to see how CellTraxx performed compared to the standard technique of manual tracking using the ImageJ/Fiji plugin Manual Tracking developed by Cordelires^2,3^ and further analysis with the Chemotaxis and Migration tool (from Ibidi, GmbH). We also compared CellTraxx to the powerful, common tracking tool, TrackMate, which is included in the Fiji version of ImageJ^13,14^. TrackMate offers the integration of a detector that relies on the deep learning-based cell segmentation tool Cellpose (CP + TM)^20,22^. For these comparisons, we used the same image series of HeLa cells as described and analysed with CellTraxx above. The cells in the image series were tracked manually by two different persons as well as being analysed by CP + TM (Movie 4). When we plot the velocity versus time (Fig. 3a) and the mean velocities of each time step (Fig. 3b), we see that the velocities generated by manual tracking correspond very well to those calculated by CellTraxx. Importantly, the velocities that we measure are within the range of velocities already reported for HeLa cells^23^. The velocities generated by CP + TM are slightly higher than for CellTraxx and manual tracking. While both CellTraxx and CP + TM track almost all cells in an image, during manual tracking only a few (minimum 40) randomly chosen cells are tracked. This is reflected in much more noise in the data generated from manual tracking (higher and lower peaks in Fig. 3a and wider spread in Fig. 3b). When only a subset of the cells were tracked by CellTraxx (by analysing an area containing approximately 40 cells), we observed more noise also in the CellTraxx data (Supplementary Fig. S2a). The nature of a population of migrating cells is generally quite heterogeneous with some cells in the population migrating fast while others might not move much at all^24^ (Movie 3). To obtain solid data, a high number of cells should be tracked. It is also worth to consider using the mode (most common) velocity or median velocity instead of the mean velocity to be less sensitive to outliers of very high velocity.

Next, we compared the actual trajectories from CellTraxx with those of CP + TM and manual tracking. Since CellTraxx can correct for image shifts which would otherwise add noise to the tracks, we decided, for a fairer comparison, to use a video where CellTraxx had performed image shift correction. The same video of HeLa cells as described above was shift corrected and used for analysis by CP + TM and manual tracking by three different persons. First, comparing CellTraxx to CP + TM, we noticed that the tracks were very similar (Fig. 3c and Fig. S2b). Moreover, the general track lengths and number of tracks were also similar (Table 1). CellTraxx had a total of 442 tracks and CP + TM found 479 tracks. A higher number of tracks could indicate more disrupted tracks, and/or tracks that the other software missed.

**Table 1:** Summary of tracking data of HeLa cells by CellTraxx and CP + TM.

	Number of cells in first image	Number of cells in last image	Total number of tracks	Number of full-length tracks	Mean track length (frames)*
CellTraxx	311	416	442	226	65
CP + TM	273^#^	356^#^	479	194	61

*Note that only tracks followed > 20 frames are included in the analysis for both CellTraxx and CP + TM, skewing the mean track length. ^#^In the case of CP + TM, only cells that are part of a track are counted, while for CellTraxx, all identified cells are included.

To compare the trajectories, we matched the tracks from CellTraxx with those of CP + TM. Tracks that started within 15 µm of each other were considered to match. The lengths of the 316 matched tracks are compared in Fig. 3d. We observed that CellTraxx and TrackMate were able to track 166 common full-length tracks. Interestingly, the two programs gave exactly the same length for several of the shorter tracks (see points along the diagonal in Fig. 3d). From the 166 full-length tracks that had common starting points, 50 randomly chosen cells were examined manually. Among these, CellTraxx had erroneously swapped to track a neighbouring cell in three cases (6% tracking error), while CP + TM only jumped in one case (2% tracking error). After cell divisions, CellTraxx and CP + TM followed different daughter cells in eight cases. CellTraxx is not set up to detect cell division events and record track inheritance, so CP + TM was run with the LAP Tracker module^25^ and similar conditions. Thus, when a cell divided, we could not consider it an error if the programs happened to follow different daughter cells.

To establish a proper and unbiased “gold truth” (commonly defined as cell positions annotated by several human operators), we randomly selected 40 tracks where CellTraxx and CP + TM followed the same cells and daughter cells through the whole video. These 40 cells were tracked manually by three different persons. The gold truth was calculated as the average cell position between the three persons for each cell and at each time step. Comparing CellTraxx and CP + TM with the mean of manual tracking (gold truth) demonstrated that both programs followed the cell trajectories very well (Fig. 3c). Plotting the corresponding trajectories obtained from manual tracking by the three persons demonstrated that also those trajectories differed somewhat (Fig. S2c).

We also calculated the differences in cell positions for the two programs compared to the gold truth. The mode (most common) difference in cell position was 2.9 µm for CellTraxx and 4.2 µm for CP + TM (Table 2, Fig. 3e). These values are quite small considering that the most common segmented cell diameter identified by CellTraxx in this video is 25 µm (Fig. 2c). Another measure for the tracking accuracy is to determine the fraction of positions that were far away from the gold truth (manual mean). We chose 10 µm as a reasonable cutoff distance and found that only 2.0% of the CellTraxx points differed more than 10 µm from the gold truth positions, while 14.4% of the CP + TM cell centres missed by more than this distance.

**Table 2:** Overview of point-to-point separation for pairwise matched trajectories from various tracking methods for HeLa cells.

	Mean (µm)	Median (µm)	Mode (µm)	Fraction > 10 µm (%)
Person 1 vs person 2	4.3	3.9	3.4	2.7
Person 2 vs person 3	3.9	3.5	3.0	2.0
Person 3 vs person 1	3.9	3.5	3.2	1.1
CellTraxx vs person 1	4.4	3.8	3.3	2.4
CellTraxx vs person 2	5.2	4.5	3.4	6.0
CellTraxx vs person 3	4.6	4.0	3.3	3.1
CellTraxx vs manual mean	4.1	3.6	2.9	2.0
CellTraxx vs CP + TM	5.3	4.2	3.6	8.0
CP + TM vs person 1	6.7	5.7	4.5	14.1
CP + TM vs person 2	7.6	6.4	4.7	19.7
CP + TM vs person 3	6.9	6.0	4.5	15.9
CP + TM vs manual mean	6.6	5.6	4.2	14.4

We also compared the velocities of the 40 full-length trajectories obtained by CellTraxx and CP + TM to the mean velocity from manual tracking (mean of measurements obtained by three persons/gold truth) (Fig. 3f). Clearly, both methods gave reasonably accurate velocities compared to manual tracking, with CellTraxx being slightly more accurate. For CellTraxx and CP + TM, the mean velocity differences to manual tracking are 0.06 µm/min and 0.11 µm/min, respectively. Also, the 95% confidence interval is approximately twice as large for CP + TM than for CellTraxx. The reason for the somewhat higher CP + TM velocity is most likely an artifact originating from the cytoplasm-based cell segmentation in Cellpose. The segmentation of the outer cell boundaries by Cellpose is generally very good. However, the cytoplasm displays more movement than the cell nucleus. Thus, when calculating the cell centre from the fluctuating outer cell boundary, there might be more noise in the cell centre coordinates than when using the centre of the nucleus. Further, in phase contrast images, the cell outline is sometimes so weak compared to the image background that Cellpose was not able to find the edge correctly (Movie 4). The nuclear-like segmentation performed by CellTraxx may generate less track noise than the cytoplasm-based segmentation by CP + TM. Thus, there was a built-in bias in favour of CellTraxx in our comparison. The added noise in the cell locations along the track will increase the track velocity, particularly for slow moving or stationary cells. For a better comparison of the two programs, we should ideally have used nuclear segmentation in the CP + TM analysis. However, we have not been able to properly segment the cells based on the nucleus in our phase contrast images, despite having tried different deep-learning image segmentation approaches such as Cellpose, Stardist^26^ or the noisy-hedgehog (10.5281/zenodo.8064806) or discreet-rooster (10.5281/zenodo.5914248) models from the Bioimage Model Zoo^27^ in combination with DeepImageJ^28^.

In summary, CellTraxx can identify and track HeLa cells. The calculated velocity is similar to that found by manual tracking and somewhat lower than our analysis by CP + TM. The trajectories obtained by CellTraxx are quite accurate compared to manual tracking. The velocities obtained by CellTraxx are generally more precise than those of manual tracking due to the higher number of cells being tracked, lowering the statistical noise in the data.

In an attempt to analyse different images captured with another microscope, we downloaded an image series of mouse muscle stem cells in hydrogel microwells (“BF-C2DL-MuSC") from the Cell Tracking Challenge (http://data.celltrackingchallenge.net/training-datasets/BF-C2DL-MuSC.zip). The hundred last images of the series were analysed using CellTraxx (Movie 5). In addition, we used TrackMate to analyse the last hundred images in a corresponding gold truth video from the same repository. The gold truth video consists of annotated images generated by several human experts in the field. Next, we compared the tracking performed by CellTraxx on the live cell images with that performed by TrackMate on the gold truth images. (For a broader discussion of the experiment, see Supplementary File S4; Comparing CellTraxx to gold truth cell positions from the Cell Tracking Challenge.) CellTraxx managed to detect the location of the real cell centres with relatively high precision (Fig. S3a,b). The mode (most common) point distance between CellTraxx and the gold truth was 2.3 µm, while 14% of the points missed by more than 10 µm. Although, CellTraxx precisely found the cell positions, the program was not particularly good at tracking the movements of the cells over long times, resulting in many small tracks (183 tracks, while TrackMate gave only 29). Despite this, the velocities based on the tracking performed by CellTraxx on the real cell video and TrackMate on the gold truth video were quite similar (Fig. S3c,e). Since the movie contains a small number of cells (15–25), there is quite a bit of statistical noise in the data (high and low peaks in Fig. S3d and wide spread in Fig. S3e). However, the fluctuations in Fig. S3d match very well between the CellTraxx analysis of live cells and the TrackMate analysis of gold truth images. This reflects the fact that the velocity measurements by CellTraxx and TrackMate are quite precise, but that the few cells move erratically from one image to the next. In summary, this test shows that CellTraxx can be used for determining cell migration velocities from other types of microscopes.

### The ability of CellTraxx to recognize and track different cell lines

**Figure 4:**
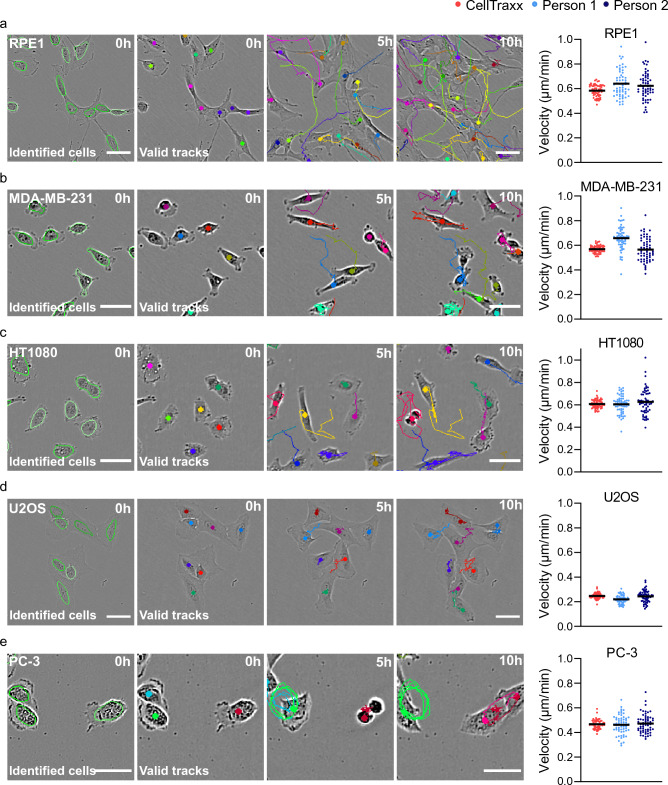
Analysis of cells with different morphologies and velocities by CellTraxx. Migrating (**a**) RPE1, (**b**) MDA-MB-231, (**c**) HT1080, (**d**) U2OS, (**e**) PC-3 cells were captured every 10 min for 10 h in Incucyte S3 and then analysed by CellTraxx. The images show an enlarged area from the movies generated by CellTraxx, illustrating identified cells at time point 0 (left image), and the same area with valid tracks at different time points. Scale bar 50 µm. The graphs to the right give a comparison of mean velocities from image to image tracked by two different persons (light and dark blue) and CellTraxx (red). Each dot represents the mean velocity from one time point to the next. The black line shows the overall mean velocity.

Next, we wanted to test the capability of CellTraxx to recognize and track cells with different morphologies and velocities. For this purpose we used: RPE1 cells (human retinal pigment epithelial cells with an elongated and thin shape, Fig. 4a, Movie 6), MDA-MB-231 (human breast adenocarcinoma cells, small in size with high contrast, Fig. 4b, Movie 7), HT1080 cells (epithelial cells derived from connective tissue from a fibrosarcoma patient with high migrating capacity, Fig. 4c, Movie 8), U2OS cells (an osteosarcoma cell line with epithelial morphology normal sized but with very low contrast and low speed, Fig. 4d, Movie 9), and PC-3 cells (a cell line originating from a bone metastasis of a prostate adenocarcinoma with high migratory capacity, Fig. 4e, Movie 10). The cells were imaged in Incucyte S3 and the image series were analysed by CellTraxx. The software seems to recognize and track the different cells very well (Fig. 4a–e, images to the left). Note that although CellTraxx can recognize most cells in all images, some cells are not included as valid tracks in the image series (see discussion). As a reference, we also tracked the cells manually using ImageJ and the Ibidi Chemotaxis program as described under *Methods*. In all cases, the velocities measured using CellTraxx were comparable to those measured by manual tracking (graphs in Fig. 4a–e). Moreover, we again found less variability in the data obtained by CellTraxx compared to manual tracking.

In the case of MDA-MB-231 and PC-3 cells, we had to use flat-field correction (optional function in CellTraxx) due to the background in the image series. This is a function included in CellTraxx to correct for uneven intensity patterns such as uneven static background illumination. Imaging with Incucyte requires the use of 96 well ImageLock plates to achieve fairly accurate repositioning during imaging. However, in some cases we can observe a background cross in our images, most likely originating from the etched pattern the Imagelock plates have for the microscope to reposition. In such cases, CellTraxx might erroneously interpret parts of the cross as cells. Usually, it is enough to increase the Gaussian filter radius, segmentation limit, “Shortest cell track” and/or reduce the highest cell velocity to avoid these errors being part of the valid tracks (see Supplementary file S1 for more information on these settings). However, sometimes this is not enough. In such cases, the CellTraxx pseudo flat-field correction might solve the problem. Movie 11 displays the same tracking of PC-3 cells as in Movie 10 but without flat-field correction. Notice all the tracks included in the middle of the images. Flat-field correction might also be useful for removing other types of static background variations.

For optimal tracking, migrating cells have to be imaged at proper intervals. A general rule of thumb is that the cells should not move more than their diameter from one image to the next in order to give reliable tracking^29^. Conversely, if none of the cells are moving more than their diameter, the images could be taken less frequently. For each image in a video, CellTraxx has to define the centre of the cell. Depending on the dark spots in the nucleus, and the general contrast of the cells, the centre point might shift slightly from image to image. If cells are imaged too frequently (for example every five minutes for slow moving cells) and not really moving much from one image to the next, more internal jumps than necessary are allowed, possibly generating an artificially high velocity. In CellTraxx, we have added the function to skip images in a series during the analysis. This will mimic longer time intervals between the images. For example, skipping every second image of a movie taken every five minutes will mimic an image series with 10-min intervals. For slow moving cells, the unwanted internal jumps will be reduced, and more real movements will be recorded. For faster moving cells, a combination of less unwanted internal jumps as well as a potential loss of some longer, true cell movements might occur. Therefore, the timing between the images should always be optimized for different cell lines.

One problem with finding the right sampling time between the images in a series, is that we often want to compare in the same analysis cells that are moving fast and slowly with the same settings such as for example upon treatment with an inhibitor or a stimulator. In these experiments, oversampling of the slower moving cells is difficult to avoid if the actual tracks of the faster moving cells should be recorded. Therefore, we have included an optional feature in CellTraxx that smoothens tracks. Figure S4 shows the same tracking of Track 155 as in Fig. 3c with and without smoothing. With one iteration (i.e. running the smoothing filter once on the track data), the track is slightly smoother, but still very similar to the original track found by CellTraxx and the manual trackers (Fig. S4). Using a higher number of iterations, the track will be even smoother, but care should be taken to avoid too much smoothening (i.e. iterations in Fig. S4). If needed, this setting can be used to better separate the speed of slow- and fast-moving cells in the same experiment. The measured velocity of slowly moving cells will be reduced considerably more with smoothing than that of the faster moving cells. This is because the small, unwanted internal jumps will be smoothed, while the actual longer cell movements are kept since the start and end points of each track remain fixed during track smoothing. The resulting valid track videos should always be examined, and the data evaluated carefully to check that the tracking dot still follows the cell centre in a reasonable manner.

### The ability of CellTraxx to analyse cells embedded in a substrate

**Figure 5:**
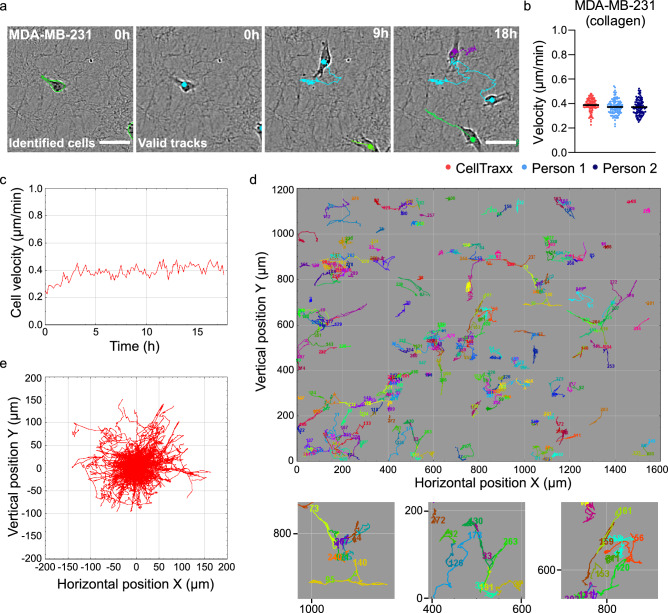
Analysis of cells in collagen by CellTraxx. MDA-MB-231 cells were embedded in collagen and then imaged every 10 min in Incucyte S3 for 18 h. (**a**) Images showing an enlarged area from the movies generated by CellTraxx, illustrating identified cells (left image) and valid tracks at different time steps. Scale bar 50 µm. (**b**) A comparison of mean velocities from image to image tracked by two different persons (light and dark blue) and CellTraxx (red). Each dot represents the mean velocity from one time point to the next. The black lines give the mean velocities. (**c**) CellTraxx plot showing velocity versus time. (**d**) CellTraxx plot representing the trajectories of MDA-MB-231 cells in collagen plotted at their actual (*X,Y*) positions. Enlarged areas are shown below with their corresponding horizontal and vertical positions indicated at the* X* and *Y* axes (respectively). (**e**) CellTraxx plot representing the trajectories of MDA-MB-231 cells in collagen plotted from a common origin.

In many cases, it can be desirable to study cells as they migrate on a substrate (such as collagen, fibronectin or Matrigel) to better mimic the extracellular environment that cells encounter in the body. Such substrates generally increase the background noise in phase contrast images substantially, causing trouble for automated segmentation and tracking of the cells. We have therefore tested if CellTraxx is able to track cells migrating when embedded in a layer of collagen. In order to keep most of the cells in the same focus plane MDA-MB-231 cells were seeded on top of a thin layer of rat tail collagen I. Afterwards, a second layer of rat tail collagen I was allowed to polymerize on top of the cells. The cells were then imaged every 10 min for 18 h in Incucyte S3. The images were analysed by CellTraxx. We observed that although the thin layer of collagen introduced an uneven background of small dark spots or fibres in the phase contrast images, CellTraxx successfully identified and tracked most of the cells (Fig. 5a, Movie 12). In addition, the velocities determined by CellTraxx were similar to those found by manual tracking (Fig. 5b). In general, the MDA-MB-231 cells in collagen moved more slowly than on plastic. The mean velocity (based on time steps) for MDA-MB-231 cells on plastic was measured to (0.57 ± 0.03) µm/min while in collagen only (0.39 ± 0.05) µm/min (the velocity is here given as the mean values first averaged for each time step and then averaged for all time steps ± one sample standard deviation calculated over all time steps). Moreover, the mean velocity of the MDA-MB-231 cells was slower in the beginning and then stabilized around 0.4 µm/min after some hours (Fig. 5c). This might be an effect of the cells not having much time to settle in the collagen matrix after seeding before imaging. In addition, we could also observe that many cells followed their own track (i.e. moving back and forth) or followed tracks of other cells (Movie 12, Fig. 5d,e). It is reasonable that the cells might follow paths in the collagen to avoid using energy on degradation of the matrix.

### Quantification of proliferation experiments using CellTraxx

**Figure 6:**
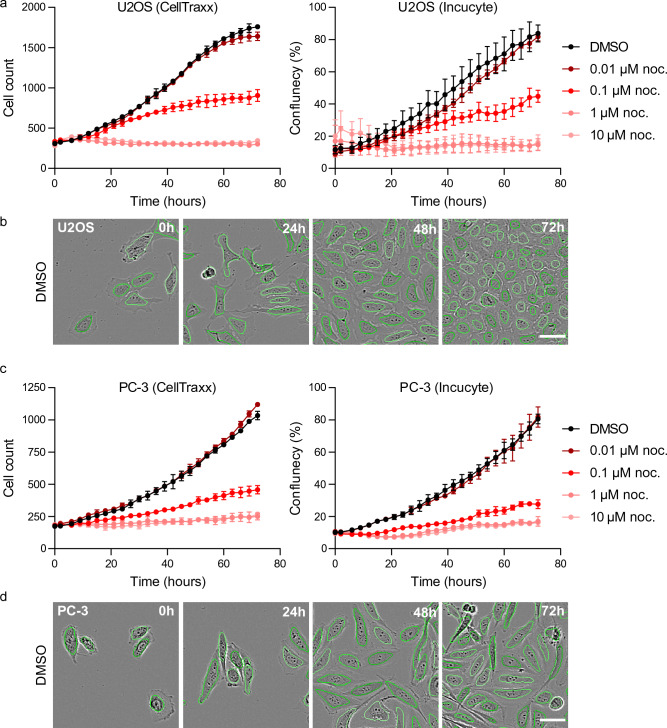
Cell counting in proliferation experiments by CellTraxx. U2OS (**a**,**b**) and PC-3 (**c**,**d**) cells treated with increasing concentrations of nocodazole (noc.) were imaged in Incucyte S3 every 3 h for 72 h. The image series was analysed by CellTraxx by counting the number of cells in each image ((**a**) and (**c**), left panel) or by the Incucyte software calculating the cell confluence over time ((**a**) and (**c**), right panel). Note that the error bars represent the standard deviation between 3 replicates from one experiment (**a**) and (**c**). Example images from CellTraxx cell counting at different time points are shown (**b**) and (**d**).

A feature of CellTraxx is that the program quite accurately segments and counts cells. CellTraxx could therefore possibly be used to determine the number of cells in proliferation and viability experiments. The Incucyte microscopes come with easy-to-use software to automatically measure the area in the well covered by cells for this purpose. In many cases, measurements of the area occupied by cells would quite well reflect the increasing/decreasing number of cells. However, in some cases it is desirable to count cells rather than indirectly measure their growth/death by area. More importantly, if cells change morphology over time (e.g. become long and thin), measurements of cell area might not properly reflect the number of cells. In such cases we recommend using CellTraxx to count cells throughout the image series. To demonstrate this principle, we treated U2OS and PC-3 cells with increasing concentrations of nocodazole and imaged every third hour for three days in Incucyte S3. Nocodazole is an agent that binds to β-tubulin and disrupts microtubule assembly/disassembly dynamics. This prevents mitosis and eventually induces apoptosis in cells^30^. Three replicate image series were analysed by CellTraxx. The number of identified cells in each image were extracted and plotted (Fig. 6a–d). The same videos were also analysed by the Incucyte software as a reference, where the confluency/area occupied by cells was estimated and plotted (Fig. 6a and c, right panel). CellTraxx accurately segmented and identified cells in the images, including the control cells that were confluent by the end of the experiment (Fig. 6b and d). For U2OS cells, which have a quite low contrast, it was difficult to find an optimal mask for area quantification and this is reflected in somewhat larger standard deviation between the replicates measured by Incucyte. However, we observed a similar reduction in viability over time with increasing concentrations of nocodazole in the data extracted from the Incucyte software and CellTraxx (Fig. 6a and c). This supports the idea that CellTraxx can be used to quantify proliferation and/or viability of cells.

### The ability of CellTraxx to analyse cells in wound healing experiments

Wound healing assays are frequently used to analyse cell migration. In these experiments, confluent cells are allowed to move into an empty area. The empty area can be made using the wound maker from Incucyte (Sartorius), by removing inserts, or simply by scratching with a pipette tip through the cell layer. To measure the migration of cells in such wound healing experiments, it is common to simply measure the area occupied by the migrating cells in the wound over time. However, the time cells use to fill an empty area might be influenced by several other factors than just their migratory properties. For example, cells with a high proliferation rate would fill the empty area faster than cells with a low proliferation rate. Thus, a treatment influencing proliferation could erroneously be interpreted as a treatment influencing cell migration, and similarly for treatments that affect cell size. In addition, these measurements are also extremely sensitive to floating cells attaching in the empty area/wound. Scratching results in floating cells that despite washing might still be present and could attach and begin to grow in the empty area. In experiments were this has happened, cell migration would erroneously seem increased. To properly measure cell migration in wound healing experiments, tracking cell movements to determine parameters such as velocity, directness, forward migration index (FMI), accumulated distance and Euclidian distance are needed.

We have provided CellTraxx with a feature that allows tracking of cells at the edge of a wound. When using CellTraxx, the user can simply select the wound healing mode available in the settings window. Then, in the tuning image, set the crop margins just outside the wound area. CellTraxx will then track the cells at the front on their way into the empty wound area.

**Figure 7:**
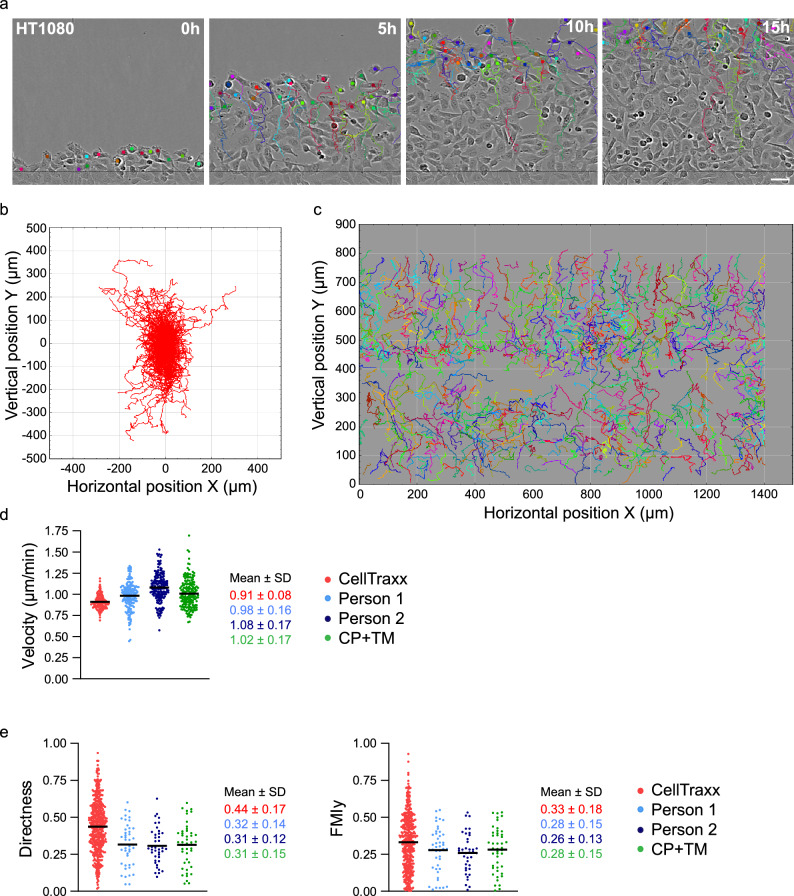
Analysis of wound healing experiments by CellTraxx. A dense layer of HT1080 cells was scratched and then imaged in Incucyte S3 every 5 min for 15 h. (**a**) An enlarged area close to the wound edge taken from the movies generated by CellTraxx, showing valid tracks at different time points. Scale bar 50 µm. (**b**) CellTraxx plot representing the trajectories of HT1080 cells migrating into the wound area plotted from a common origin. (**c**) CellTraxx plot representing the trajectories of HT1080 cells migrating into the wound area plotted at their actual (*X,Y*) positions. (**d**) A comparison of mean velocities from image to image tracked by CellTraxx (red), two different persons (light and dark blue) and CP + TM (green). Each dot represents the mean velocity from one time point to the next. The black lines show the mean velocities. (**e**) A comparison of the directness (left) and vertical forward migration index (FMIy) (right) for each track analysed by CellTraxx (red), two different persons (light and dark blue), and the 45 longest tracks from CP + TM (green). Each dot represents the mean directness/FMIy for each track. Note that CellTraxx analysis generated a total of 491. The black lines show the mean.

To investigate how well CellTraxx is able to estimate the cell front velocity during wound healing, we have performed an experiment using HT1080 cells. The cell monolayers were scratched and imaged in Incucyte S3. The videos were then analysed by CellTraxx as well as by manual tracking for comparison. Using the wound healing mode in CellTraxx, the program tracked cells at the front of the wound along the top and bottom edges (Fig. 7a, Movie 13). Plotting the trajectories from a common origin as well as their original (*X,Y*) position illustrates that most of the cells were moving into the wound (Fig. 7b,c).

The cell front velocity estimated by CellTraxx, and by manual tracking was comparable (Fig. 7d). We also analysed the cells using CP + TM. In an attempt to track cells in the front of the wound, we cropped the video to approximately one row of cells (the front) was left on each side of the wound. In addition, we analysed only the 45 longest tracks (Movie 14). The velocities obtained using CellTraxx matched quite well with those from CP + TM (Fig. 7d). As observed before, the data generated by CellTraxx is less variable than the data generated by manual tracking, mainly due to the few cells tracked manually. This was also the case for the data from CP + TM, since only 45 tracks were included.

Due to the very high cell density, we noticed that CellTraxx prematurely stopped and started several tracks. Only approximately 10% of the tracks were followed in more than half of the frames. Although 166 cells were recognized in the first image, we ended up with a total of 491 tracks. Therefore, metrics such as accumulated distance and Euclidian distance should not be used based on this tracking. However, we compared metrics such as directness and FMIy (Forward Migration Index in the vertical direction) measured for each track by the different methods (Fig. 7e). Even though most of the tracks from CellTraxx were chopped into shorter tracks, CellTraxx estimated the mean directness and mean FMIy similarly to manual tracking by two different persons as well as those calculated from the 45 longest tracks by CP + TM. Taken together, CellTraxx is able to track cells at the front of the wound in a wound healing experiment but care should be taken when estimating metrics based on track length.

### CellTraxx analysis of PC-3 cells stimulated with HGF

We further wanted to test that CellTraxx can be used to investigate different cellular migratory capabilities in both single cell random migration experiments as well as in wound healing experiments. To this end, we examined PC-3 cells stimulated or not with hepatocyte growth factor (HGF). HGF stimulation of PC-3 has previously been shown to increase their velocity^31^. The cells were imaged in Incucyte S3 and analysed using CellTraxx (Movies 15 and 16, single cell random migration, and Movies 17 and 18, wound healing). Three parallel samples were included for both conditions in both experiments to compare the accuracies of tracking from sample to sample.

**Figure 8:**
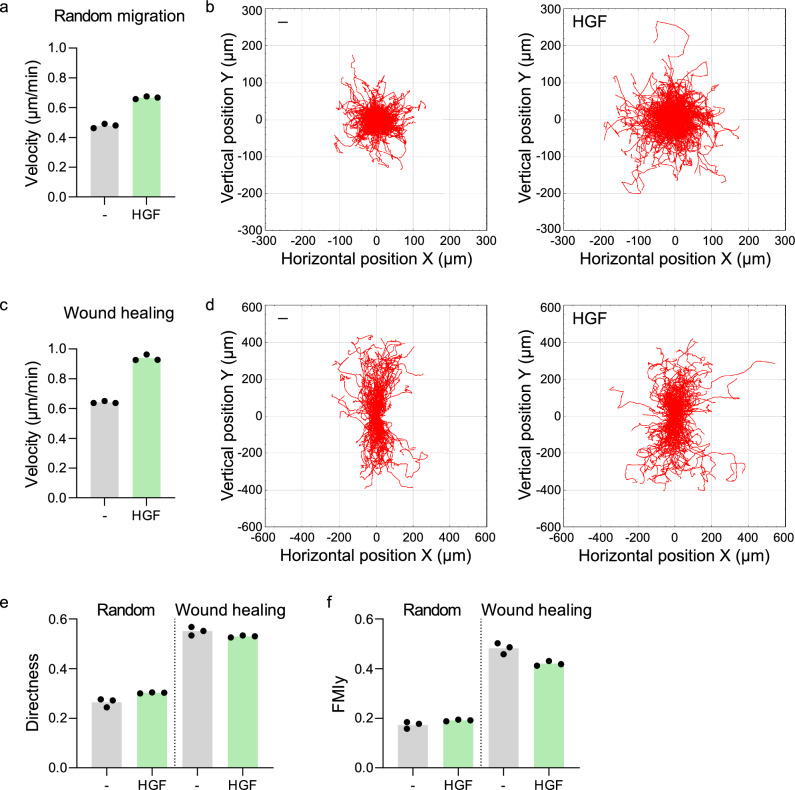
Analysis of HGF-stimulated cells by CellTraxx in single cell random migration and in wound healing. PC-3 unstimulated or stimulated HGF was compared in a single cell random experiment and in a wound healing experiment. For the random migration experiment, cells were imaged every 10 min for 10 h and for the wound healing experiment, cells were imaged every 5 min for 14 h. Three replicate samples were analysed by CellTraxx. (**a**) The mean velocities per track of cells unstimulated or stimulated with HGF in the random cell migration experiment are plotted. Each replicate is marked by a dot, while the bar represents the mean of the three replicates. (**b**) CellTraxx plot representing the trajectories of PC-3 cells unstimulated or stimulated with HGF in the random cell migration experiment plotted from a common origin. One replicate for each condition is shown. (**c**) The mean velocities per track of cells unstimulated or stimulated with HGF in the wound healing experiment are plotted. Each replicate is marked by a dot, while the bar represents the mean of the three replicates. (**d**) CellTraxx plot representing the trajectories of PC-3 cells unstimulated or stimulated with HGF in a wound healing experiment plotted from a common origin. One replicate for each condition is shown. (**e**) The directness calculated by CellTraxx is plotted for the three replicates from the random cell migration and the wound healing experiments described above. (**f**) The FMIy calculated by CellTraxx is plotted for the three replicates from the random cell migration and the wound healing experiments described above.

As expected, we observed an increase in the velocity of PC-3 cells stimulated with HGF in both the single cell random migration experiment (Fig. 8a,b) as well as in the wound healing experiment (Fig. 8c,d). The directness and FMIy was also measured for the PC-3 cells in both random migration and wound healing experiments, of which the latter gave increased values whether stimulated with HGF or not (Fig. 8e,f). In addition, we observed that the three replicate samples for all conditions were very similar, indicating robust measurements of cell migration by CellTraxx.

## Discussion

We have shown that CellTraxx, a novel automated cell migration tracking tool for label free, phase contrast images, precisely tracks the migratory path of cells of different speed and different morphology. We have demonstrated that the precision of CellTraxx is similar to that of manual tracking which is the current standard tracking tool used for label free, phase contrast images in many laboratories. In addition, due to automation, CellTraxx enables tracking of several hundred cells in one image series, improving the precision and increasing the robustness of the data compared to manual tracking, where normally only 30–50 cells are tracked per image series. We propose that CellTraxx can be used for high throughput cell migration screens and different types of migration assays.

CellTraxx has been tested by several persons with and without previous experience in ImageJ and tracking of cells. They found CellTraxx to be intuitive and user friendly when run through the ImageJ macro. A detailed user manual which can be found in Supplementary File S1, gives clear instructions and guidance on how to use CellTraxx. We have also included some recommendations for settings, since the most challenging task for a new user is to find the best parameters. CellTraxx can run as an integrated part of ImageJ with interactive settings and tuning windows. To keep CellTraxx simple and easy to use, the most important settings are easily adjusted in the ImageJ interface while some additional settings can only be changed by editing the celltraxx_defaults.txt text file (See *Advanced running* in Supplementary File S1). We recommend testing the chosen settings on a small dataset, e.g. one image series or part of an image series before starting an analysis on a large dataset of many similar videos. Advice on how to examine the settings is also included in the CellTraxx *User Manual* (Supplementary File S1). Although CellTraxx is good at segmenting cells and tracking them, with an automated analysis, there will always be a compromise to find the best settings with the least errors.

For many labs, manual tracking of cells is still the preferred method for quantifying cell migration. This is due to the lack of accurate and easy-to-use automated tracking tools, despite the large number of tracking software available^5^. Manual tracking is not only labour-intensive, but also highly susceptible to user-dependent errors. First of all, to reduce labour, only a small subset of cells in an image series is analysed. Thus, manual tracking relies heavily on the selection of a representative subset of cells. This was evident when comparing the manual tracking of two different persons (see Fig. 4b). Secondly, and along the same line, the fewer cells being tracked, the less precise are the calculated average velocities. A population of moving cells is normally quite heterogeneous with some cells hardly moving and some cells moving a lot. Therefore, the selection of representative cells is extremely important. When few cells are tracked, the inclusion of *one* extremely fast cell makes a much larger difference than when many cells are being tracked. Thirdly, determination of the cell centre (as in where to “point and click”) which serves as measuring points for cell positions during manual tracking, is also highly variable. In 2010, Huth and colleagues^32^ estimated the results of manual tracking to miscalculate velocities by up to 410%. Many of these problems are avoided with our automated tracking tool. However, as mentioned above, we do observe that also with CellTraxx the cell centre determination is influenced by internal movements of organelles within cells, resulting in some small artificial movements.

We have chosen to use Incucyte S3 phase contrast images for CellTraxx analysis. Incucyte is a user-friendly semi-high throughput microscope available in many laboratories. However, we have shown here that CellTraxx may work adequately for determining cell velocities also for images from other microscopes, provided that the cells have enough contrast relative to the background. For fluorescence images with bright cells on a dark background, one would need to reverse the contrast before using CellTraxx to analyse such image series. Our attempts to track cells from the Cell Tracking Challenge repository show that contrast reversal may be needed also for non-stained cells.

We have further shown that CellTraxx measures velocities in the same range as manual tracking, which is also comparable to what has previously been reported for the tested cell lines. However, it is worth mentioning that the velocities calculated by CellTraxx are not absolute numbers and will be heavily influenced by the settings. For example, increasing the “Highest cell velocity” beyond a physically reasonable value would in most cases increase the measured overall velocity. This is because the analysis would allow more erroneous track jumps from one cell to another which increases the number of very high velocity events. Therefore, all samples in the same experiment should be run with the same settings.

Although CellTraxx is able to recognize most cells in all images, some cells are not included in valid tracks in the image series. The user defines how long a track has to be in order to be included in the analysis. At the end of a movie, after a cell division or if a cell moves into the analysis region, there might not be enough time steps left for CellTraxx to generate a new track. We therefore often observe cells that are not part of a valid track at the end of an image series—especially if the “Shortest cell track” parameter is high. In addition, some tracks are prematurely halted, for example if a cell bumps into another in such a way that they are difficult to distinguish. Moreover, a track might prematurely halt if the cell for some reason becomes smaller or larger than the accepted sizes. Therefore, there will always be some cells in an image series that are not part of a valid track.

In wound healing experiments, since the cells are rather dense, some cells might overlap or even cross paths. Such cells are hard to distinguish, even during manual tracking. Therefore, it might be difficult for CellTraxx to track the same cell continuously from the beginning to the end of a wound healing experiment. Thus, more tracks will start and stop than during single cell random migration tracking. This is important to keep in mind when looking at the data obtained. For the measurements of cell front velocities, stopping and starting new tracks should not make a large difference. However, other measures such as the directness, accumulated and Euclidian distance, as well as FMI might be sensitive to this and should be interpreted with caution. We recommend to always examine the videos with valid tracks carefully and also consider stopping tracking before the wound is completely filled. Note also that the valid track videos generated by CellTraxx only include tracks where the cells are still present in the image. To examine all valid tracks in an image series, use the trajectory plots generated by CellTraxx (Fig. 2g).

We see several advantages of CellTraxx (Table 3). However, since all tracking software represents a compromise between different choices, CellTraxx also has some disadvantages or limitations compared to alternative tracking programs (Table 3). Despite these limitations, we believe that CellTraxx may fill a niche among the existing cell tracking tools. Since CellTraxx can analyse a large number of similar image series without user interference once the analysis parameters have been determined, this opens up for semi- and high-throughput cell migration screening experiments. We hope that CellTraxx will become a widely used and valuable tool for many researchers who study cell migration.

**Table 3:** Advantages and disadvantages with CellTraxx.

**Advantages:**
Distributed free of charge
Relatively easy installation
User friendly interface through ImageJ macro
Option for image shift correction
Option for pseudo flat-field correction
Option for wound healing analysis
Automatic and robust segmentation grey level algorithm
Runs without user interference after initial tuning of parameters
Can process thousands of videos with hundreds of images in one run
Designed for phase contrast images
Works for several different cell types with different morphologies
No training needed (not based on machine learning)
Precise detection of cell nucleus positions
Saves all results in a specific folder
Stores a variety of numerical results, charts and videos for easy checking of the data
Stores the settings used for future reference
Relatively fast execution (Running the full analysis of Cellpose + TrackMate took 50 min, while the corresponding analysis in CellTraxx took 8 min on the same PC. Thus, CellTraxx runs about 6 times faster.)
**Disadvantages:**
Does not detect cell outline (cytoplasm borders)
Does not detect cell divisions
Does not generate cell inheritance tree (lineage)
Designed for Incucyte S3 images (other microscopes may require special adaptions)
May jump from one cell to another during tracking more often (6%) than software which segments the full cell outline (2%)
No option to edit or correct tracks
The provided executable celltraxx.exe works only on Windows computers but the C source code can be compiled for any system

## Methods

### Cells

hTERT-RPE1 (CRL-4000), MDA-MB-231 (HTB-26), and PC-3 (CRL-1435), were purchased from ATCC. HT1080, U2OS, and HeLa cells were a generous gift from the Department of Molecular Cell Biology, The Norwegian Radium hospital. U2OS, HeLa, and HT1080 cells were grown in DMEM (Cat#D6546, Sigma-Aldrich) supplemented with 10% foetal bovine serum (FBS) (Cat#F7524, Sigma Aldrich) and 2 mM l-Glutamine (Cat#G8541, Sigma Aldrich). MDA-MB-231 cells were grown in RPMI-1640 (Cat#R8758, Sigma-Aldrich) with 10% FBS. hTERT-RPE1 cells and PC-3 cells were maintained in DMEM-F12 medium (Cat#31331028, Gibco, ThermoFisher) with 10% FBS. In all cases, the media was supplemented with 100 U/mL Penicillin and 100 µg/mL Streptomycin (Cat#P4458, Sigma Aldrich). All cell lines were grown in a 5% CO_2_ atmosphere at 37 °C. The cell lines were tested negative for mycoplasma contamination by PCR and their identity was verified using Powerplex16 assays.

### Random migration and wound healing assay

Cells were seeded sparsely (2500–5000 cells per well) or densely (40,000–70,000 cells per well) into Incucyte® ImageLock 96-well plates (Sartorius) for random migration and wound healing assays, respectively. For the wound healing assays, confluent cells were scratched using the Incucyte 96-well Wound maker tool (Sartorius) and washed once to remove floating cells and prevent reattachment in the wound area. For both assays, the cells were imaged in phase contrast using the 10X objective in Incucyte S3 Live Cell Analysis System with Incucyte S3 software (Sartorius). Images were taken every 10 or 5 min (as indicated) for different periods of time. In some cases (as indicated), the cells were treated with 50 ng/mL HGF (Cat#H5791, Sigma Aldrich). Images were exported from Incucyte S3 as AVI-files “as displayed” without compression and image series were further processed for cell migration analysis.

### Collagen assay

Incucyte® ImageLock 96-well plate was coated with 35 µL of ice cold 2.0 mg/mL collagen rat tail type 1 (Cat#354236, Corning) in 1X MEM (10X, Cat#2027185, Gibco) and 25 µM Hepes (1 M, Cat#15630-056, Gibco). The pH of the collagen solution was adjusted to pH 7.5 using 0.34 N NaOH. After polymerization for 5 min at 37 °C in 5% CO_2_, the collagen was washed gently with ice cold PBS (1X, Cat#14190-144, Gibco). MDA-MB-231 cells in RPMI-1640 medium with 10% FBS was then added (3000 cells/well) on top of the polymerized collagen and the plate was incubated for 45 min at 37 °C in 5% CO_2_. The medium was gently removed and approximately 50 µL of the 2.0 mg/mL collagen solution was added to each well to form the top layer of collagen. RPMI-1460 medium with 10% FBS was added to the collagen-embedded cells, and the cells were kept for 45 min at 37 °C before live-cell imaging using the Incucyte S3 phase contrast microscope. Images were taken every 10 min for 18 h.

### Cell migration analysis

Cell tracking was performed manually using the ImageJ Manual Tracking plugin developed by Cordelieres, then using the Chemotaxis and Migration Tool (Ibidi, GmbH, https://ibidi.com/chemotaxis-analysis/171-chemotaxis-and-migration-tool.html) to calculate migration parameters. CellTraxx was used for automated tracking and data analysis, see Supplementary File S1.

For Cellpose + TrackMate analysis, cell segmentation was performed using Cellpose v2.2.3^20,22^ with custom model LC2 (see https://cellpose.readthedocs.io/en/latest/models.html) and Cell diameter = 30 pixels. The default value 0.4 for flow threshold gave the best segmentation. A crop margin of 20 pixels from all four sides was used in CellTraxx when aligning the images using image shift correction for the HeLa cell video. The resulting images were converted to an image stack in ImageJ, 20 pixels from each side were cropped off and the stack saved as a TIF file for processing by the TrackMate plugin in Fiji^3,13,14^. For tracking of the cells segmented by Cellpose, we used the LAP tracker^25^ with max distance = 32 pixels, and gap closing allowed with max distance = 32 pixels, and max frame gap = 1. This tracker and these settings were the most relevant tracking conditions when comparing to CellTraxx.

For comparison of tracking with the Cell Tracking Challenge training dataset BF-C2DL-MuSC 01, we used the last 100 images of the series (t1275.tif–t1375.tif). The gold truth cell coordinates for 29 tracks were found by letting TrackMate in Fiji analyse the corresponding manually annotated images (man_track1275.tif – man_track1375.tif) in the 01_GT folder. The 16-bit images were first converted to a binary image and then inverted to get white spots (255) on a grey background (123) such that the thresholding detector could be used with a threshold grey level of 150 to easily distinguish all 2000 spots in this image series. The LAP tracker was again used for tracking the spots, but with more liberal gap closing settings in order to track the fast-moving spots as well as possible: max frame gap = 4, alternative linking cost factor = 1.05, linking max distance = 60 pixels, gap closing max distance = 100 pixels, allow gap closing = true, allow track splitting = false, allow track merging = false. The resulting spot coordinates were sorted by frame number and then by track ID before being imported into Microsoft Excel (Microsoft Corporation). CellTraxx was used to analyse the corresponding real, live cell images with the following settings: Perform flat-field correction = yes, Pixel size = 0.645 µm, Gaussian filter radius = 10 µm, Smallest cell diameter = 6 µm, Largest cell diameter = 30 µm, Cutting cell diameter = 20 µm, Top crop margin = 22 pixels, Bottom crop margin = Left crop margin = Right crop margin = 10 pixels, Time between images = 5 min, Highest cell velocity = 10 µm/min, Shortest cell track = 5 images, Segmentation Limit =– 1.5. (A negative Segmentation Limit, overrides the automatic segmentation and uses instead as grey level limit the mean grey level + Segmentation Limit * the grey level standard deviation of the image. For more information, see Supplementary Files S1, S2) The two sets of cell coordinates from the CellTraxx analysis of the live cell images and the gold truth from TrackMate's analysis of the 01_GT images were compared in Excel as described below to determine the segmentation precision and calculate the cell velocities.

### Comparisons of trajectories

In order to quantify how well the software could determine cell positions compared to a gold truth based on human evaluation, values for track number, image number and cell centroid coordinates (*X,Y*) were pasted into Excel from both datasets. Since the order of tracks was typically different between the two methods, tracks were matched by requiring that the first cell positions in a track were closer than 15 µm from each other. Making a plot of the matched tracks allowed us to confirm that the matching was good. Once the cell coordinates were aligned on the same rows for all tracks and all image numbers (time steps), a column with the point-to-point distance for all cell positions was calculated. The FREQUENCY() function in Excel was used for generating a distance distribution table with 1 µm bin size. Normalizing the distance distribution so its sum would be 100%, and plotting the distribution along with its accumulated version, gave a chart from which the most common (mode) distance and the percentage of distances larger than 10 µm could be extracted. To get precise values for the distance mode, the LINEST() function in Excel was used for fitting a parabola to the peak of the distance distribution curve. The top point coordinate of the parabola was taken as the mode distance. The percentage of points with separation > 15 µm was found by linear interpolation on the accumulated distribution.

To compare the cell velocity of matched tracks for the two methods, two other columns were generated in Excel to store the distance moved by a cell from one image to the next. By dividing these columns by the time between images, two new columns where all the velocity values for all steps in all matched tracks were listed for the two methods. The mean and median velocities were calculated by using the corresponding functions in Excel, while the mode velocity was found as described above by generating a distribution plot and fitting a parabola to find the exact peak position.

### Proliferation assay

U2OS and PC-3 cells were seeded at 3000 cells per well in a 96 well plate (Nunclon Delta Surface, Cat#167,008, Thermo Scientific). The following day the cells were either treated with DMSO (control) or with increasing concentrations (0.01–10 µM) of nocodazole (Cat#1404, Sigma Aldrich). The cells were then imaged every 3^rd^ hour for 72 h in Incucyte S3. The image series were exported as uncompressed AVI files and analysed by CellTraxx (see Supplementary File S1 for instructions on the analysis). For comparison, the image series were also analysed using the basic analyser software from Incucyte S3.

### Supplementary Information

Supplementary Movie 1.

Supplementary Movie 2.

Supplementary Movie 3.

Supplementary Movie 4.

Supplementary Movie 5.

Supplementary Movie 6.

Supplementary Movie 7.

Supplementary Movie 8.

Supplementary Movie 9.

Supplementary Movie 10.

Supplementary Movie 11.

Supplementary Movie 12.

Supplementary Movie 13.

Supplementary Movie 14.

Supplementary Movie 15.

Supplementary Movie 16.

Supplementary Movie 17.

Supplementary Movie 18.

Supplementary Figures.

Supplementary Information 1.

Supplementary Information 2.

Supplementary Information 3.

Supplementary Information 4.

Supplementary Legends.

## Data Availability

Due to their large size, example videos had to be stored on Dropbox. The link to the Dropbox folder is given in the file WhatToDo.txt in the GitHub repository. A representative movie of HeLa cells (the same as analysed and presented in Figs. 2 and 3) with the analysis settings used, as well as a representative movie of PC-3 cells in a wound healing experiment (one of the replicates from Fig. 8c–f), are available in the folder "Example_videos_for_testing". All the results files generated when analysing these two files can be found through the same link in the folder "Example_videos_for_testing_Results". All the primary data are available from the corresponding author on request.

## Code availability

The source code for "celltraxx.exe" was written in C and is available at https://github.com/borge-holme/celltraxx_download. The user-friendly interface for CellTraxx is implemented as a macro for ImageJ/Fiji. The macro code and the required associated files can be downloaded from the same repository by downloading the "celltraxx_system" folder.
