# Supplementary material for: Automated tracking of cell migration in phase contrast images with CellTraxx
Source: Sci Rep. 2023 Dec 27;13:22982. doi: 10.1038/s41598-023-50227-9 (PMC10752880; doi:10.1038/s41598-023-50227-9)
Supplement: Supplementary file 19 — Supplementary Figures. [file 41598_2023_50227_MOESM19_ESM.pdf]

Figure S1

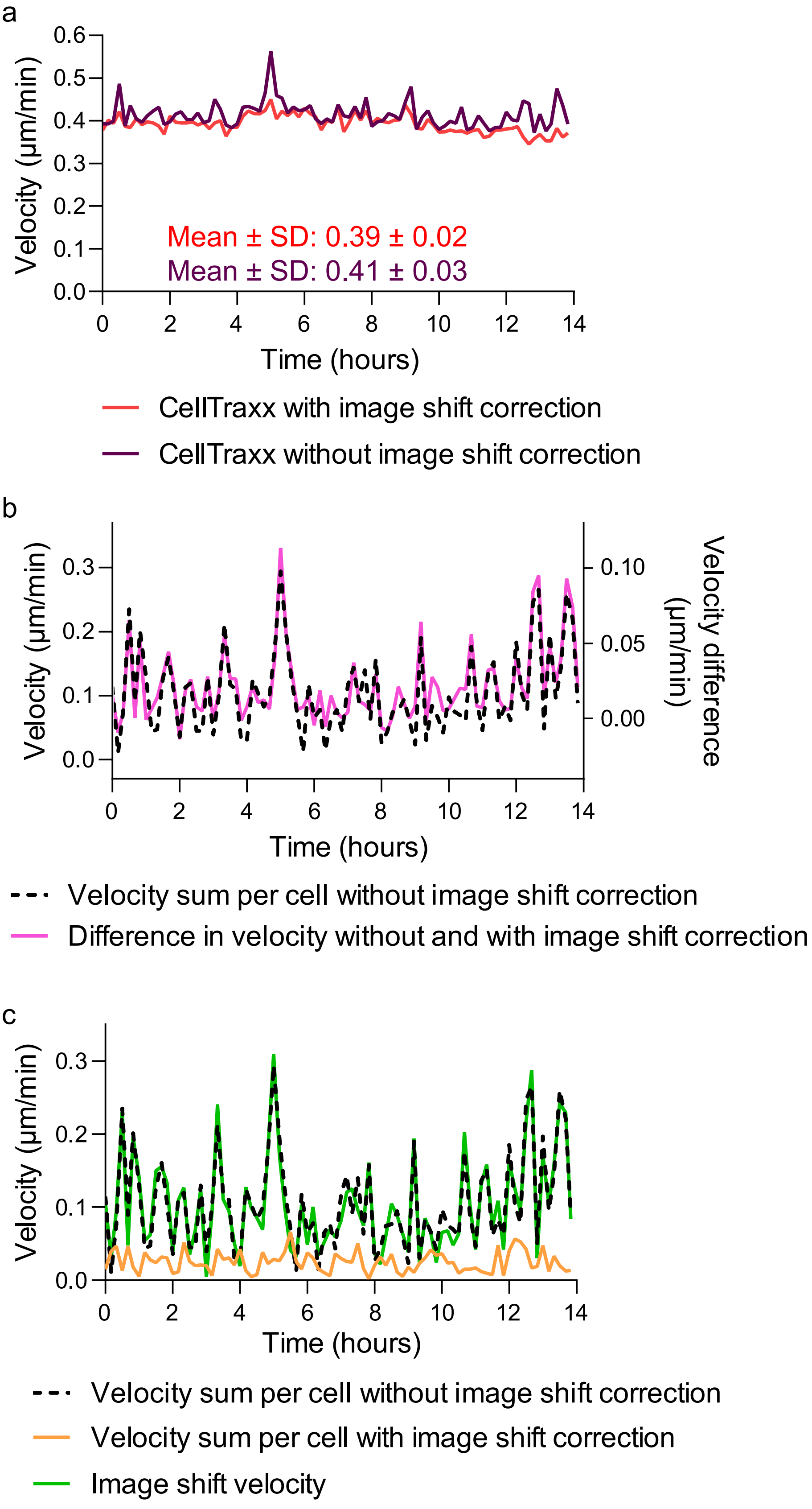
a
Velocity (µm/min)
0.6
0.5
0.4
0.3
0.2
0.1
0.0
Mean ± SD: 0.39 ± 0.02
Mean ± SD: 0.41 ± 0.03
0 2 4 6 8 10 12 14
Time (hours)
CellTraxx with image shift correction
CellTraxx without image shift correction
b
Velocity (µm/min)
0.3
0.2
0.1
0.0
0.10
0.05
0.00
Velocity difference (µm/min)
0 2 4 6 8 10 12 14
Time (hours)
Velocity sum per cell without image shift correction
Difference in velocity without and with image shift correction
c
Velocity (µm/min)
0.3
0.2
0.1
0.0
0 2 4 6 8 10 12 14
Time (hours)
Velocity sum per cell without image shift correction
Velocity sum per cell with image shift correction
Image shift velocity

Figure S1. **Image shift correction.** The data presented in this figure are generated from an image series of HeLa cells, imaged every 10 minutes for 14 hours in Incucyte S3. The images were then analysed by CellTraxx with or without image shift correction. Note that the image series analysed here is the same as in Figure 2 and 3. (a) The graph represents the mean velocity over time (from image to image) *with* image shift correction (red) and *without* image shift correction (purple). The mean velocities ± one standard deviation are written with corresponding colours. (b) A comparison of the difference between the two curves in (a) (magenta curve) and the "Velocity sum per cell" over time (dashed, black curve). The “Velocity sum per cell” is the length of the vector sum of all cell velocities divided by the number of cells. (c) A comparison of the "Velocity sum per cell" with image shift correction (orange curve) and without image shift correction (dashed, black curve. The dashed, black curve is the same as in (b). When images are well aligned, the value stays below 0.1 µm/min. The green curve shows the velocity of the image shifts (= shift distance / time interval) as detected by CellTraxx during image shift correction.

Figure S2

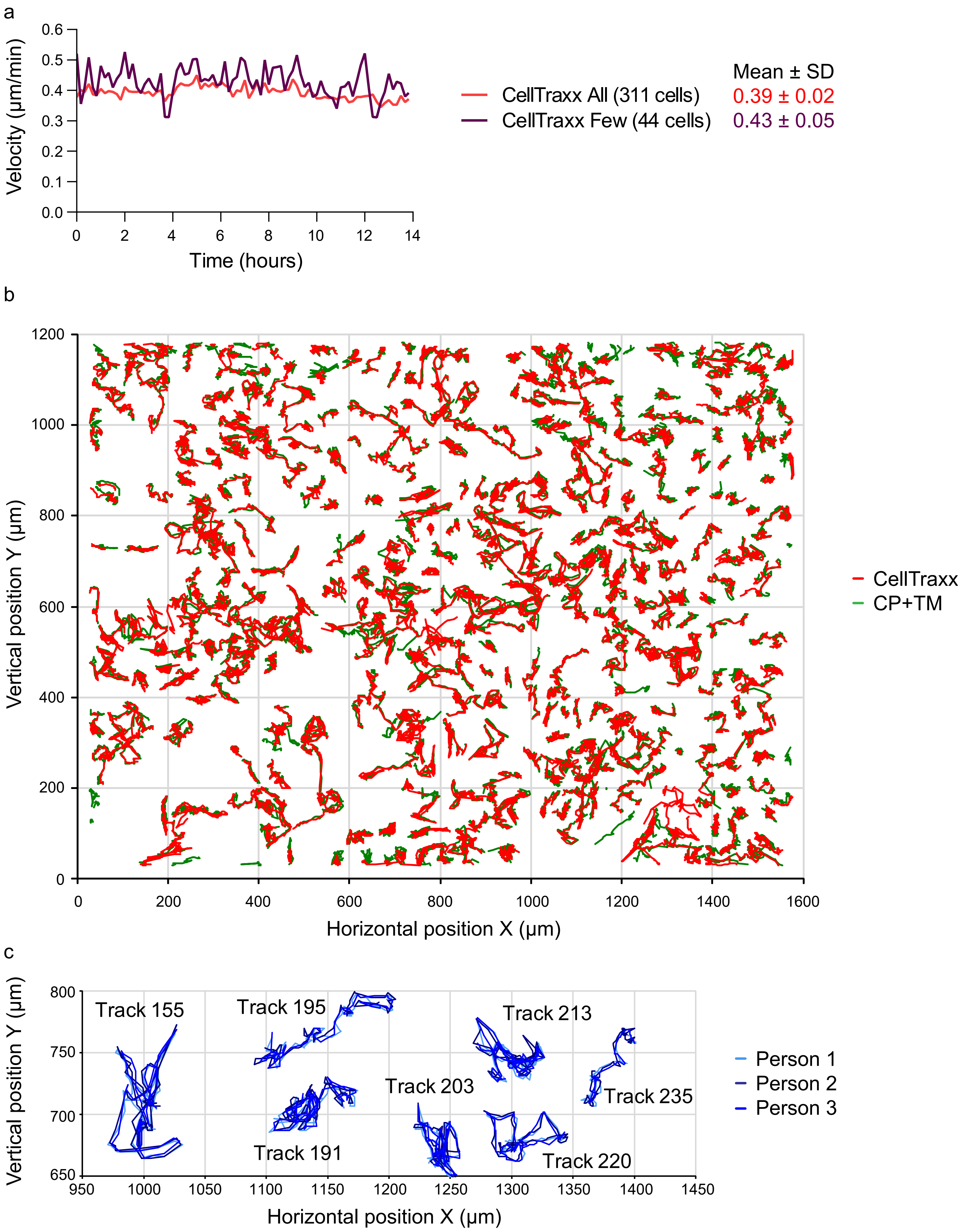

a
Velocity (µm/min)
0.6
0.5
0.4
0.3
0.2
0.1
0.0
0
2
4
6
8
10
12
14
Time (hours)
Mean ± SD
CellTraxx All (311 cells)
0.39 ± 0.02
CellTraxx Few (44 cells)
0.43 ± 0.05
b
Vertical position Y (µm)
1200
1000
800
600
400
200
0
0
200
400
600
800
1000
1200
1400
1600
Horizontal position X (µm)
CellTraxx
CP+TM
c
Vertical position Y (µm)
800
750
700
650
950
1000
1050
1100
1150
1200
1250
1300
1350
1400
1450
Horizontal position X (µm)
Track 155
Track 195
Track 213
Track 191
Track 203
Track 235
Track 220
Person 1
Person 2
Person 3

Figure S2. **Comparison of tracking methods.** The data presented in this figure are generated from an image series of HeLa cells, imaged every 10 minutes for 14 hours in Incucyte S3. The images were then analysed by CellTraxx. Note that the image series analysed here is the same as in Figure 2 and 3. (a) The graph represents the mean velocity over time (from image to image) of the whole image series containing 311 cells in the first image (red) and a cropped area of the image series containing 44 cells in the first image (purple). The mean velocity ± one standard deviation is noted in corresponding colours. (b) The trajectories tracked by CellTraxx (red) and CP+TM (green) plotted as in the movie. Note that the analysis for CP+TM were run on image shift corrected videos. (c) The seven same single tracks presented in Figure 3c, tracked by three different persons (different shades of blue) are presented in the plot. The tracks are numbered with their track number from the CellTraxx analysis. Note that the analysis for manual tracking and CP+TM were run on image shift corrected videos.

Figure S3

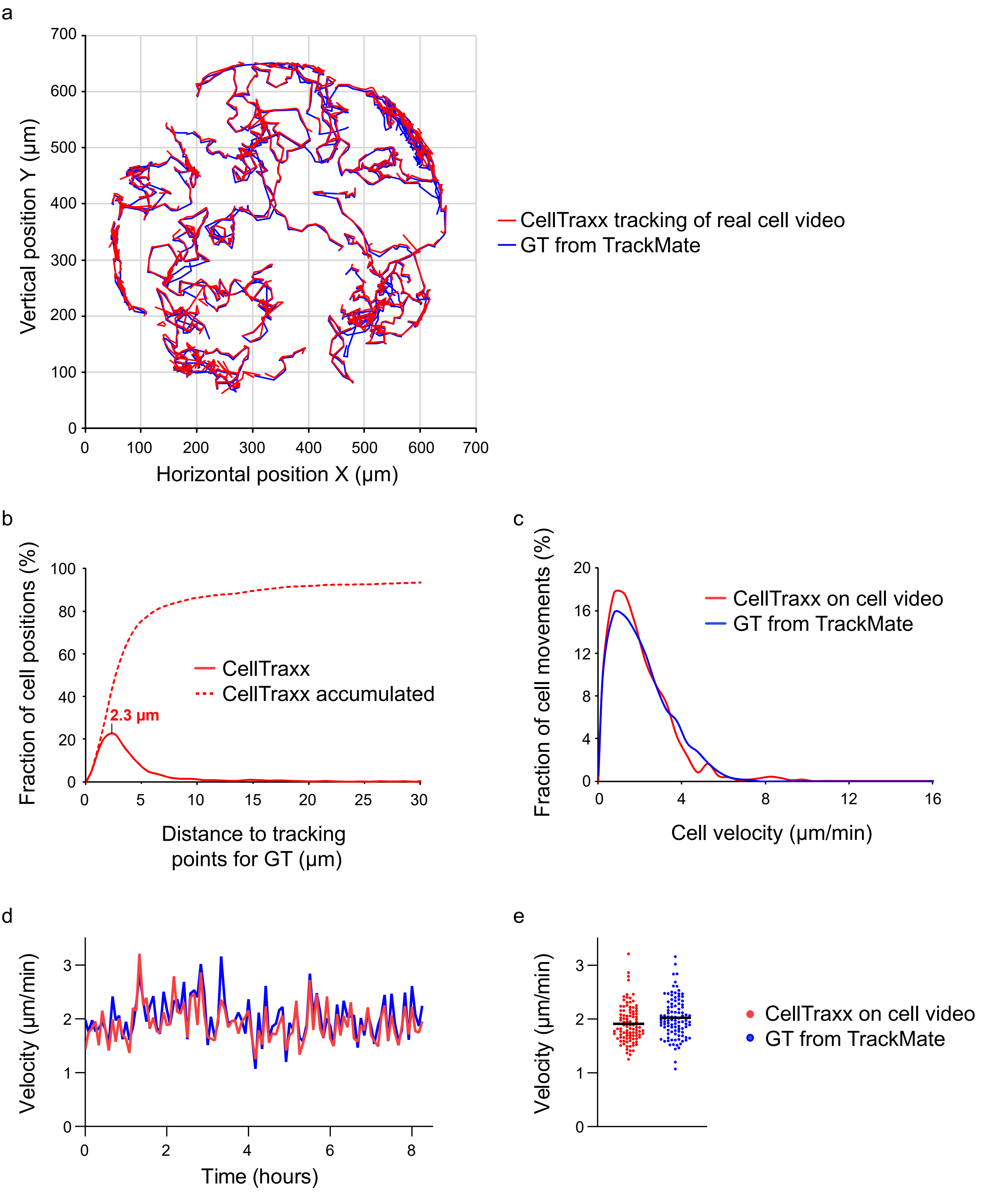
a
Vertical position Y (µm)
Horizontal position X (µm)
0
100
200
300
400
500
600
700
CellTraxx tracking of real cell video
GT from TrackMate
b
Fraction of cell positions (%)
Distance to tracking points for GT (µm)
CellTraxx
CellTraxx accumulated
2.3 µm
0
20
40
60
80
100
5
10
15
20
25
30
c
Fraction of cell movements (%)
Cell velocity (µm/min)
CellTraxx on cell video
GT from TrackMate
0
4
8
12
16
20
d
Velocity (µm/min)
Time (hours)
0
1
2
3
4
6
8
e
Velocity (µm/min)
CellTraxx on cell video
GT from TrackMate
0
1
2
3

Figure S3. **Cell Tracking Challenge.** Analysis of images number 1275 – 1375 from the training dataset BF-C2DL-MuSC 01 in the Cell Tracking Challenge repository. (a) Plot with the 29 tracks found by TrackMate's analysis of the gold truth (GT) annotated images generated by several human experts in the Cell Tracking Challenge for the BF-C2DL-MuSC 01_GT dataset (blue lines) overlaid by the 121 matched tracks from the CellTraxx analysis of the corresponding real cell images (red lines). (b) The normalised distribution of distances between pairs of cell positions from the gold truth data and the CellTraxx analysis. (c) Plot showing the cell velocity distribution curves calculated from the TrackMate analysis of the gold truth data and the CellTraxx analysis of the corresponding microscope images, based on matched tracks. (d) A comparison of mean velocity over time calculated from tracking by CellTraxx of the real cell images (red) and by TrackMate analysis of the corresponding GT images (blue). (e) A comparison of the mean velocities from image to image calculated from tracking by CellTraxx of the real cell images (red) and by TrackMate analysis of the corresponding GT images (blue). Each dot represents the mean velocity from one time point to the next. The black line shows the overall mean velocity.

Figure S4

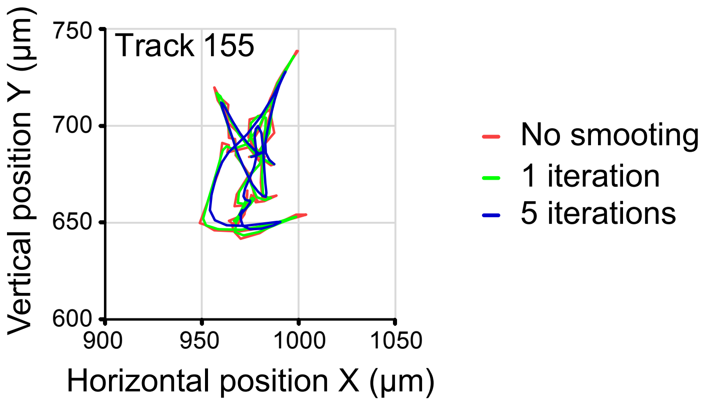

Figure S4. Track smoothing. Plots showing a single track as detected by CellTraxx without smoothing (red), with 1 smoothing iteration (green), and with 5 smoothing iterations (blue). The track shown here (number 155) is the same as in the left part of Figure 3c.
