# Supplementary material for: Automated tracking of cell migration in phase contrast images with CellTraxx
Source: Sci Rep. 2023 Dec 27;13:22982. doi: 10.1038/s41598-023-50227-9 (PMC10752880; doi:10.1038/s41598-023-50227-9)
Supplement: Supplementary file 20 — Supplementary Information 1. [file 41598_2023_50227_MOESM20_ESM.pdf]

CellTraxx User Manual

Version 4.6

Contents

Materials (software and data) 1
Preparations 2
  Installing the software 2
  Image files 3
Running CellTraxx 4
  1. ImageJ 4
  2. Settings window 4
    General settings 4
    Segmentation 6
    Tracking 7
    Output 8
  3. Interactive tuning 8
  4. The command window 10
CellTraxx output 11
Validation of the data 19
Wound healing mode 20
Cell counting (e.g., proliferation experiments) 21
Troubleshooting 21
Advanced running 23

Materials (software and data):

- The CellTraxx executable `celltraxx.exe` (see next chapter for instructions on how to install CellTraxx).
- ImageJ or Fiji, version 1.53t or newer (https://imagej.nih.gov/ij/download.html https://imagej.net/software/fiji/downloads).
- Image series to analyse, AVI video files or bitmap images (see next chapter for more information).
- May need the "Microsoft Visual C++ 2015-2019 Redistributable (x64)" package if it is not already installed on the computer. See *Troubleshooting*, page 22 for further details.

Preparations:

Installing the software:

1) Use a web browser to open the CellTraxx GitHub repository

https://github.com/borge-holme/celltraxx_download/tree/main

2) Click the green **[Code]** button and select **Download ZIP**.

3) Open the **Downloads** folder, right-click the icon of the file `celltraxx_download-main.zip` , choose **WinZip** in the menu and select the option **Unzip to here**.

4) Open the new folder `\celltraxx_download-main\` which appears in the **Downloads** folder.

5) Copy the entire folder `\celltraxx_system\` to the root of your C disk drive so its path becomes `C:\celltraxx_system\`. This folder name is hard-coded in `celltraxx.exe` and cannot be changed (unless the code is recompiled).

6) Copy the **CellTraxx User Manual** and the remaining files and folders in `\celltraxx_download-main\` to a more permanent location for future reference.

The `\celltraxx_system\` folder contains the following files:

`celltraxx.exe` - the software which performs the image analysis.

`celltraxx_100um.bmp` - an image file used by celltraxx.exe to add scale bars to output images if desired.

`celltraxx_bitmaps2process.txt` - text file read by celltraxx.exe containing the names of all bitmap images to analyse in the right order. This text file is automatically updated when running the ImageJ macro, but can also be edited manually if running celltraxx.exe without ImageJ (see *Advanced running*).

`celltraxx_defaults.txt` - text file read by celltraxx.exe, and updated by the ImageJ macro, containing all adjustable settings. For more information, see *Advanced running*.

`celltraxx_error_messages.txt` - a text file written by celltraxx.exe and read by the ImageJ macro to convey error messages. Any valid error messages from celltraxx.exe are automatically displayed in a message box on the computer when running the macro in ImageJ. Alternatively, open this text file to check for error messages.

`celltraxx_imagej_macro.ijm` - ImageJ macro providing a user-friendly interface for converting AVI videos to bitmap images, obtaining and tuning the desired command file settings, running celltraxx.exe and post processing output data to AVI videos and charts.

The following files are required for using the tuning tool in ImageJ:

`celltraxx_tuning_image_inset.bmp` – an empty version of the top ribbon in the tuning window displayed by ImageJ.

`celltraxx_tuning_info.txt` – an ancillary file to allow CellTraxx to remember previous settings.

`tuning_temp` – is a folder which is automatically generated when running the CellTraxx as a place to store all standard results files generated during tuning. After analysing many different videos, this folder will have accumulated a large number of temporary files, so empty the folder regularly to free up disk space.

The files in the `\celltraxx_system\` folder can be used for advanced running of the program outside ImageJ, (see *Advanced running*, page 23).

Image files:

The main program celltraxx.exe reads a series of phase contrast images stored as uncompressed bitmaps. The ImageJ macro will convert uncompressed AVI video files to bitmaps if a new AVI file is present in the data folder. Download your image series as uncompressed AVI files from the Incucyte or other microscopes and store in a folder named `X:\celltraxx_data\` that can be placed on any disk drive (X) on your computer. Note that the folder has to be placed directly on the root of the disk you choose, as the program will not find the folder if it is hidden in a sub-folder. Also, avoid space characters in folder names. Copy AVI files to the `X:\celltraxx_data\` folder before running CellTraxx. Then copy the desired results back to a suitable folder or simply rename the results sub-folder after the analysis is finished. The program will run faster if the `celltraxx_data` folder is located on the local hard drive (`C:\`). But if limited space, an external disk (e.g., `D:\`) can be used. CellTraxx will analyse all AVI files that are present in the `X:\celltraxx_data\` folder, so no explicit listing or selection of files to process is necessary.

From the Incucyte software, download the images as AVI videos in the following way:

Select **Export** > **As displayed** > **Next**, select the wells to export > **Next**, select timepoints **>** **Next**, select folder, choose type of file: **AVI no compression** > **Export**

Tip: File names cannot contain any spaces, use underscore (_) instead.

Tip: We recommend to store Incucyte files with VID number and well coordinates (as proposed by Incucyte; `VID1234_B2_1.avi`). In the output data from CellTraxx, well coordinates are included as separate columns to allow sorting of data based on the well coordinates (see below under, CellTraxx Output, page 11).

Tip: Image series captured by Incucyte must be generated using Incucyte® ImageLock 96-well plate (Sartorius, Item no: BA-04856) to ensure a more accurate (X,Y) repositioning form image to image. We also recommend only one image per well as this seems to increase the accuracy of the (X,Y) repositioning.

Tip: If images are taken with the 10X objective and exported without any scaling, the pixel size is 1.24 µm/pixel.

Running CellTraxx:

1. ImageJ

For a user friendly and interactive interface, the program is run from ImageJ. For experienced users or if tuning is not required, there is also a possibility to run the celltraxx.exe directly from a command window or by simply double-clicking the celltraxx.exe icon (see *Advanced running*).

Open ImageJ or Fiji. Select **Plugins** in the ImageJ toolbar, > **Macros** > **Install**… Find the folder C:\celltraxx_system\ and select the file celltraxx_imagej_macro.ijm before clicking **[Open]**. To run the macro, select **Plugins** > **Macros** > **celltraxx_imagej_macro**. With this method, the macro needs to be installed every time ImageJ is reopened. If you rather want to permanently install the CellTraxx macro in Fiji, copy the file celltraxx_imagej_macro.ijm into the folder C:\Users**<your_username>**\ImageJ**<2>**\Fiji.app\plugins\Macros\ where **<your_username>** should be the name of the local user who installed Fiji. Depending on the ImageJ version, the sub-folder can be called \ImageJ\ or \ImageJ2\. After a restart of Fiji, the macro can be started from the Fiji menu as described above (i.e., **Plugins** > **Macros** > **celltraxx_imagej_macro**). Alternatively, just drag the celltraxx_imagej_macro.ijm macro file from the C:\celltraxx_system\ folder into ImageJ and press **[Run]** whenever you want to start the macro.

> *Tip: Do not keep open any of the CellTraxx files in the C:\celltraxx_system\ folder, nor the output files generated while CellTraxx is running. This could cause the program to halt.*

2. Settings window

Two windows appear when the celltraxx_imagej_macro.ijm starts: ImageJ Log and CellTraxx Settings. The Log window will show the status of the analysis (do not close while running), whereas the CellTraxx Settings window is used to input the required parameters. See Figure 1.

General settings:

Settings parameters to adjust (the values from the previous run are given as defaults):

Wound healing mode: Check this box to select wound healing mode. In this mode only the cells closest to the wound are tracked. See *Wound healing mode*, page 20 for extra information and tips when using this feature.

Perform flat-field correction: Check this box to carry out a pseudo flat-field correction. If so, the acronym "_FFC" will be added to new images generated in X:\celltraxx_data\ and at the end of the results sub-folder to remind the user that flat-field correction was employed. A flat-field correction renormalizes pixel intensities across an image to correct for uneven illumination or slowly varying background patterns. Flat-field correction should only be used when images contain an uneven, static background and should be used with care as it sensitizes the segmentation. Flat-field correction is normally not necessary on image series from Incucyte, and we recommend keeping it off as default. The pseudo flat-field correction

in CellTraxx manages without a true, blank background image, which is normally needed. Instead, it requires that cells are not too dense and that they all move around enough so that the most common grey level over time in any pixel represents the background shade.

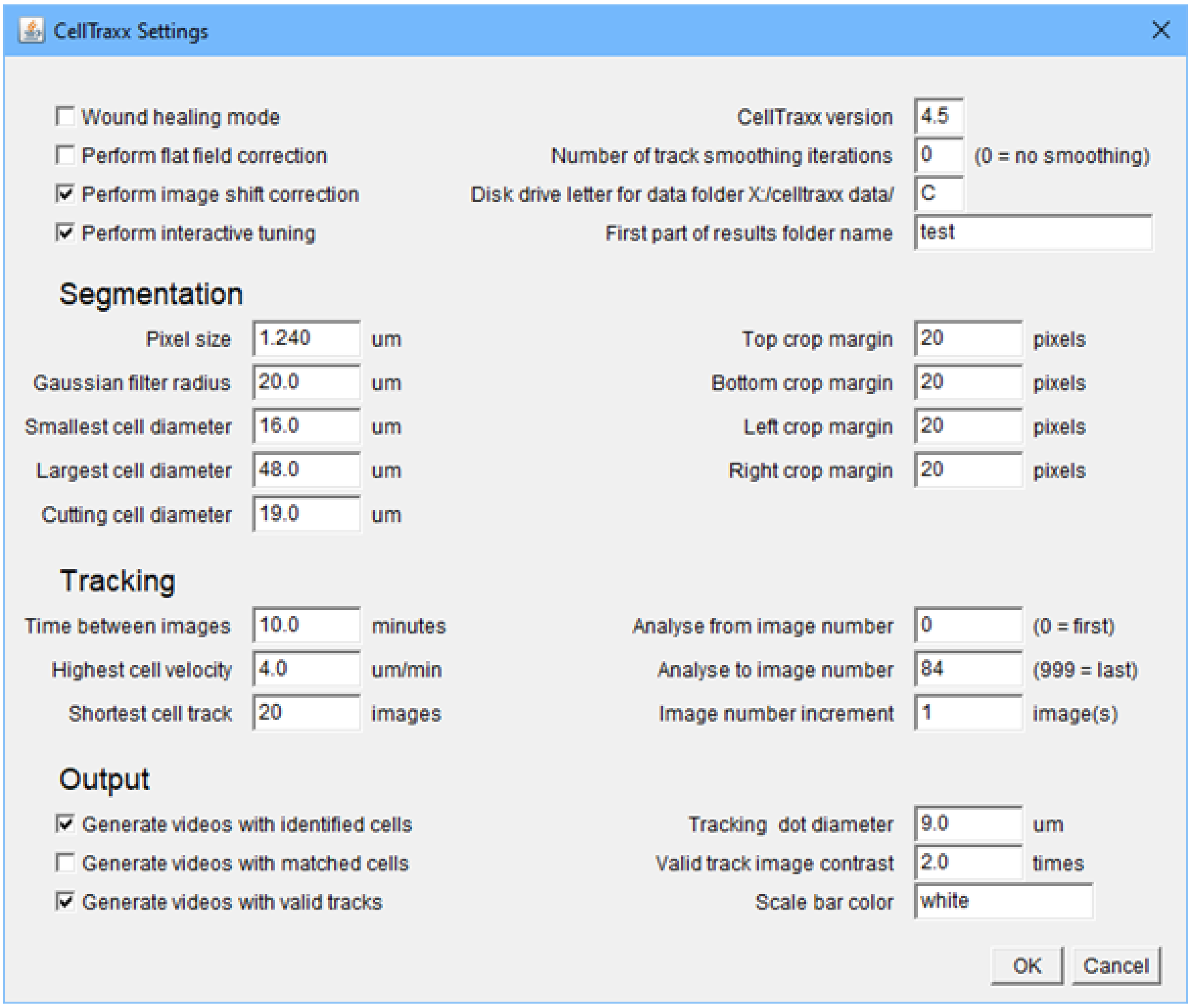

Figure 1. The CellTraxx Settings window as displayed when the ImageJ macro starts.

Perform image shift correction: Check this box to include correction of poor image alignment. Misalignments occur when the microscope is not able to find back to the exact (*X*,*Y*) position when returning to a previous well location. Performing image shift correction extends the running-time by typically 20 %. However, for Incucyte image series, we recommend to always include image shift correction to increase the accuracy.

Perform interactive tuning: Uncheck this box if you do not need to use the interactive tuning window for adjusting the cell segmentation parameters when [OK] is clicked. For convenience, this box is set to be checked by default in the ImageJ macro.

CellTraxx version: The version number of the program needs to match the number which is encoded in celltraxx.exe. An error message occurs if the version number is not correct. To find the CellTraxx version number, check the file; `celltraxx_defaults.txt` in the `C:\celltraxx_system\` folder.

Number of track smoothing iterations: This setting allows smoothening of the cell trajectories found by CellTraxx. It can be used to remove noise from the trajectories. CellTraxx finds the midpoint of the cells based on the dark spots inside the cells (mainly the nucleoli). Sometimes, these spots move, thus the midpoint of the cell is slightly shifted, even if the cell's periphery is not moving. This effect may cause small, artificial movements of the cell centre found by CellTraxx. For slow moving cells, such small, artificial movements might make a significant contribution to the measured velocity of the cells. We have therefore included the option to smooth the tracks to reduce this noise while retaining the start and end points of the tracks. Choose the number of iterations (smoothings) to be performed. If chosen, we recommend 1-2 iterations. Always carefully check that the tracking dot still follows the cell centre in a reasonable manner. If "0" is written, track smoothing is not performed. As default, we recommend no track smoothing.

Disk drive letter for data folder X:/celltraxx_data/: Setting the letter of the disk where the `\celltraxx_data\` folder is found, e.g., "C" if the data are stored on the local disk `C:\celltraxx_data`, or for instance "F" if an external disk with this designation is used for CellTraxx data storage. (See *Preparations, Installing the software*, page 2).

First part of results folder name: The results/output data are automatically stored in a sub-folder inside the `X:\celltraxx_data\` folder (see *Preparations*, *Image Files* and **Disk drive letter for data**). Any desired text string added to the default folder name can be given here. In addition, the output folder name will automatically be given the following suffix "`_GFR=??um_d=??-??um_Cut=??um`" where "GFR" denotes Gaussian Filter Radius, "d" gives the accepted diameter range and Cut shows the selected cutting diameter. The question marks correspond to the different chosen values. No white space characters are allowed and will be replaced by underscore. This combination of a user selected first part and a tuning specific second part of the folder name makes it easy to keep a good overview of all results when different settings are tested to optimize an analysis.

Segmentation

Pixel size: The lateral size of the pixels in the image series. Pixels are assumed to be square shaped. Images exported from the Incucyte and made with the 10X objective have a pixel size of 1.24 µm.

Gaussian filter radius: To separate the cells from the background, CellTraxx blurs the images using a Gaussian filter. Increasing the Gaussian filter radius increases the blurring and typically makes the cell outline rounder. Reducing the radius may give better separation of denser or low contrast cells. For good segmentation, the optimal value for the Gaussian filter radius can be tested in the interactive tuning window (see chapter 3). Good values for the Gaussian filter radius are typically between 12 µm and 22 µm but vary from cell line to cell line. We recommend using the highest Gaussian filter radius that still separates the cells well.

Smallest cell diameter: This parameter excludes all identified objects with a diameter below the given value. CellTraxx uses the equivalent circular diameter which is the diameter of a circle with the same area as the identified object, regardless of its roundness. Use this parameter to avoid tracking small regions such as debris or floating cells. Notice that "Smallest cell diameter" refers to the equivalent diameter of the region *outlined* by CellTraxx, which is generally smaller than the outer diameter of the cell. The CellTraxx equivalent diameter is influenced by the Gaussian filter radius. For medium sized cells such as HeLa, a typical value would be 13-17 µm.

Largest cell diameter: Sets the upper limit for the diameter of an object to be included as a cell. Larger regions will be scrapped. Note that also this parameter refers to the equivalent circular diameter outlined by CellTraxx and not the actual cell diameter. For medium sized cells such as HeLa, a typical value would be around 50 µm.

Cutting cell diameter: This parameter is used by the special CellTraxx cutting routine which tries to separate merged cells by cutting at narrow, concave points on the regions' edges. CellTraxx uses a complex test to determine where to cut. But the value of this parameter should generally be set to a few microns larger than "Smallest cell diameter".

Top / Bottom / Left / Right crop margin: Only cells within the crop margins will be tracked by the software. Images exported from Incucyte may sometimes contain black lines at the edges (in some cases only a few pixels wide). Since these black regions would interfere with the analysis, we recommend to always include a small crop margin (i.e., 20 pixels from all directions) for image series exported from Incucyte. If only a smaller part of the image is relevant to analyse, the crop margins can be set accordingly. It is also possible to use a small analysis window to speed up an initial test run of new data. See *Wound healing mode*, page 20 for information on how to set the crop margins for analysis of wound healing experiments.

Tracking

Time between images: The time (in minutes) between consecutive images in all videos to be analysed in the same run.

Highest cell velocity: This parameter sets an upper limit to how far a cell may move from one image to the next. A too low value could erroneously end a track if the cell made a particularly long jump. A too high value might allow a track to jump to a neighbouring cell. A good starting value for an image series of relatively fast-moving cells (average velocity 0.6-0.8 µm/min) would be 5-6 µm/min. See *Validation of the data*, page 20 for more information on how to find optimal settings for this parameter.

Shortest cell track: Used for determining which tracks to include in the statistical analysis and in the “valid track” videos. This parameter enables the exclusion of tracks shorter than the given number of images. A high value will ensure that only cells that were tracked over many images are analysed. However, one may then lose several cell tracks where a cell is lost in two or more consecutive images (due to for example low contrast) and then found again. New tracks that begin less than "Shortest cell track" number of images before the end of the video will be excluded since they can never reach the given limit. The shortest cell track cannot be set to less than three images.

Analyse from image number: Allows the user to choose at which image in the video the analysis will begin. This is convenient for long image series where only the middle or last part includes interesting changes. Note that CellTraxx will give image number 0 (zero) to the first image of a video.

Analyse to image number: Gives the last image number to include in the analysis. If set to a value larger than the number of images (in e.g., 999), the series up to the last available image will be analysed. Be aware that image series exported from Incucyte often contain a duplication of the last image at the end of the series. In addition, time point 0 is image number 1 from Incucyte. Therefore, to avoid the duplicated image when analysing the whole image series, set "Analyse to image number" to 2 less than the number of images in the AVI video.

The number of your image series can be found in the upper left corner after opening/dragging your AVI file into ImageJ.

Image number increment: Used for setting if every image (1), every second image (2), every third image (3) and so on, should be analysed. This can simulate the effect of longer time between capturing images. One can also save analysis time by using a value larger than 1 if cells do not move much between consecutive images.

Output

Generate videos with identified cells: When checked, CellTraxx generates AVI videos showing identified cells (i.e., cells which are accepted with the current settings) outlined in nuances of green. Note that here all identified cells are outlined even if not part of a track. Recall that the cell outline defined by CellTraxx is usually smaller than the actual cell outline and is influenced by the Gaussian filter radius. Examine these images if CellTraxx is used for quantifying cells (e.g., for proliferation experiments).

Generate videos with matched cells: Check this box to make CellTraxx generate AVI videos which show the cells that are accepted with the current settings and are matched with cells in the previous image. Note that these cells are not included in the valid track videos if the track they belong to is shorter than "Shortest cell track".

Generate videos with valid tracks: When the box is checked, CellTraxx will produce AVI videos where only valid tracks are shown. These are the tracks that are used in the statistical analysis and contribute to the various results CSV files. The track lines and cell centres of mass are drawn in the video images with matching colours which are different from one track to the next. Valid tracks are defined by the settings "Longest cell movement" and "Shortest cell track". Only tracks that belong to cells which are still visible in the current image are shown. This means that if a cell has moved outside the cropping frame or the cell is lost, its track is no longer displayed in the video.

Tracking dot diameter: Sets the size of the tracking dot which can be displayed at the cell centre of mass in the output images. A value of 8 µm is good for middle sized cells. Decrease this number to avoid blocking too much of small cells. A value of zero gives no tracking dot.

Valid track image contrast: This option makes CellTraxx increase the contrast within the cropping frame in the output images. Default value 2 makes the cells more visible without looking artificially enhanced.

Scale bar colour: The scale bar is automatically set to 100 µm and placed in the lower right corner of the output images. The colour of the scale bar and text can be the decided by writing a colour. Valid names are “white”, “black”, “red”, “green”, “blue”, “cyan”, “magenta”, “yellow”, “orange”, “darkred”, “darkgreen”, “darkblue” and “pink”. The scalebar is only included in the output videos if a valid colour name is written.

Press **[OK]** to continue or **[Cancel]** to abort.

3. Interactive tuning

After pressing **[OK]**, the interactive tuning window appears, see Figure 2. The command window where `celltraxx.exe` is being run once in tuning mode will also appear for some seconds and then close automatically. The tuning mode allows for interactive adjustments of

the important segmentation parameters; Gaussian filter radius, smallest and largest equivalent cell diameter, and cutting diameter. After pressing the **[Update]** button or changing between displaying the **[First]**, **[Middle]** or **[Last]** image, the `celltraxx.exe` program is again executed with the current settings. This typically takes a few seconds, using longer time for a large filter radius and large analysis frame. As soon as the command window closes, the effects of the new settings are displayed in the CellTraxx Tuning Image window.

Even if several image series are about to be analysed simultaneously when multiple AVI video files are present in the `X:\celltraxx_data\` folder, the interactive tuning tool only displays images from the first video. The rationale for this is that the other videos should have been made under similar conditions such that a common set of analysis settings can be used for all the videos that are analysed together. If some videos require different settings, either remove those AVI files from `X:\celltraxx_data\` or temporarily give them a different extension (like `.avi_HIDDEN`) to prevent CellTraxx from analyzing those files during the next run.

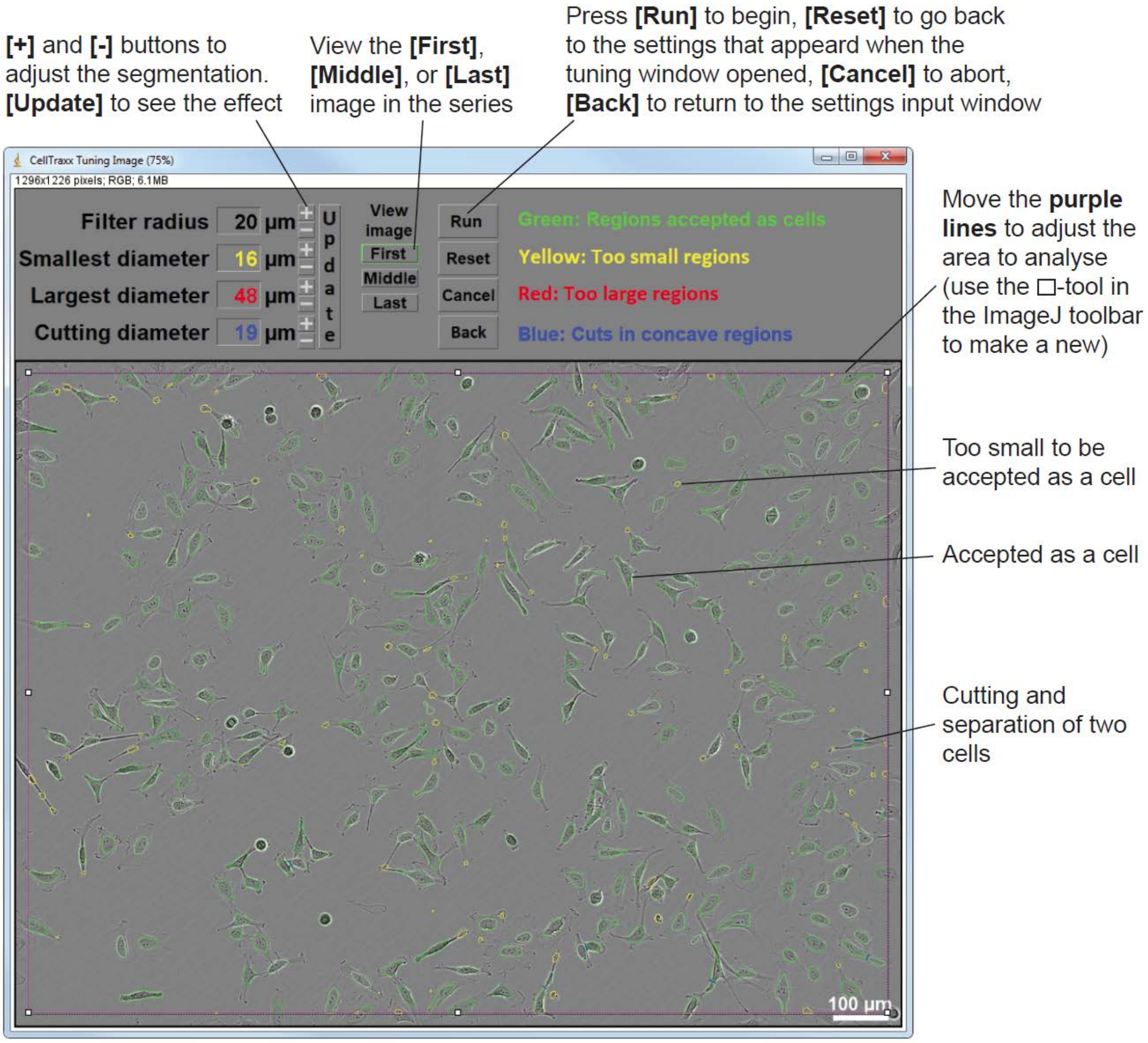

Figure 2. The interactive tuning window as displayed when running CellTraxx through the ImageJ macro. Note that there are no red areas shown in this image since no regions are too large.

Tip: To zoom in and out, simply hold the cursor over the interesting spot in the image and press + or -. To move around without losing the cropping frame, choose the "scrolling tool" (hand icon) in the ImageJ toolbar. If the crop margin is lost, use the rectangle tool to draw it again and press ***[Update]*** *to see the effect.*

Tip: Many versions of ImageJ will change the cursor to a grey + when hovering inside an image. For Incucyte images with generally grey backgrounds this renders the cursor nearly invisible. To keep a normal, white pointer arrow as cursor at all times, select **Edit** > **Options** > **Misc…** *and check the box before "***Use pointer cursor***" in the ImageJ menu.*

In the interactive tuning window, the Gaussian filter radius, the smallest and largest cell diameters, and the cutting diameter can be adjusted by clicking the **[+]** or **[-]** buttons (see **Running CellTraxx**, **Segmentation** above). The corresponding values are immediately updated in the CellTraxx Tuning Image window. However, to see how the analysis will run with the new settings, one must click **[Update]** (or **[First]** / **[Middle]** / **[Last]**) to allow `celltraxx.exe` to be executed and then show how the new settings changed the segmentation results. For convenience, the buttons **[First]**, **[Middle]** and **[Last]** are included to allow the user to see how the current settings affect the segmentation of the first, middle or last image to be analysed, as given by the current parameter settings. For long time series it is often useful to check if settings that look good for the first image will also work for the last image when the cells are typically more densely spaced.

The magenta cropping frame can be moved or resized to select a desired area to analyse. Click and drag the white boxes at the corners or edges to resize. To move the frame, click inside the frame and keep the mouse button down while moving the frame, then release the mouse button. Click **[Update]**, **[First]**, **[Middle]** or **[Last]** to see the effects of a new cropping frame. To create a new cropping frame if the magenta frame is lost, use the rectangle selection tool in the ImageJ toolbar and draw the new frame by click, keep the mouse button down, move to the opposite corner and release.

Regions in the tuning image outlined with various shades of green are areas accepted as cells. Yellow regions are too small and red regions are too large to be accepted in the analysis (see Figure 2). The "Smallest diameter" value should be chosen such that all debris particles and other objects too small to be a cell appear with yellow outline. The "Largest diameter" should be made small enough that any neighbouring cells which become merged even with the best choice of "Filter radius" and "Cutting diameter" are drawn with red outline. This excludes merged double or triple cells from further analysis.

Tip: The analysis of the tuning image is based on only that image. When running CellTraxx, the program uses additional information from the previous and the following image to aid the identification of cells in the current image. Therefore, the images in the final videos may contain accepted cells that were not shown as identified in the tuning window.

Press **[Run]** to begin the analysis of all available AVI files using the current settings. Press **[Reset]** to reload the settings that appeared when the tuning window opened. Press **[Back]** to go back to the CellTraxx Settings input window, or press **[Cancel]** to abort.

4. The command window

After pressing **[Run]**, a command window which is running `celltraxx.exe` opens and the progress of the analysis can be followed in that window. The window closes automatically when `celltraxx.exe` is finished. Do not close the command window during execution.

However, if you need to stop `celltraxx.exe` while running, say after starting it by mistake, select the command window and press the keys **[Ctrl]**+**[C]** or [Ctrl]+[Break] simultaneously. When `celltraxx.exe` is finished, the ImageJ macro takes over again and performs post processing of the output bitmap images and CSV results files produced by `celltraxx.exe`. When all is finished, the Log window displays: “Finished processing all .avi videos in the folder X:/celltraxx_data/”.

If a new AVI video file is present in the `X:\celltraxx_data\` folder, the ImageJ macro converts it to individual bitmap images that can be read by `celltraxx.exe`. If such images are already present, the macro saves time by not repeating the conversion. The bitmap images and CSV results files generated by `celltraxx.exe`, and the corresponding AVI videos and plots made by the ImageJ macro are all stored in the same sub-folder under `X:\celltraxx_data\`... Data are overwritten if exactly the same folder name has been used before. Therefore, make sure to change the first part of the results folder name text string if old data generated on the same videos with the same settings should be kept.

> *Tips! When running a new analysis with a different set of AVI videos from another experiment, it is convenient to rename the whole* `X:\celltraxx_data\` *folder containing the previous run, by adding a suffix to the folder name. Then create a new folder called* `X:\celltraxx_data\` *and move the next AVI video files to analyse into this folder. An even faster alternative if the AVI videos are already stored in a folder on the root directory of the data disk (e.g., in* `X:\My_avi_files\`) *is to simply rename this folder to* `X:\celltraxx_data\` *so no copying of large video files is needed.*

CellTraxx output

The output folder (e.g., `X:\celltraxx_data\My_cells_GFR=19um_d=15-40um_cut=14um\`) contains the following files:

Summary files for the whole run:

> *Tips! To read the CSV files, open the files in Excel, select the first column and then go to* **Data** > **Text to columns** > select **Delimited** > **Next** > select **Comma** > **Finish**

`_Grey_level_curves_for_first_images.csv` - Celltraxx.exe analyses the first image in each video to automatically find a good grey level limit for segmenting cells. This file summarises the frequencies of the grey values in the whole image and in the background. A plot can be made of the data in case there is suspicion that the segmentation has not been optimal in some video.

`_Identified_cells_summary.csv` - This file summarises the number of identified cells in each image in all videos analysed. The data can be used for example for quantification of cell number over time (i.e., for proliferation experiments).

`_Log_window_summary.csv` - This file stores the tabular information that was written to the command window while `celltraxx.exe` was running. The data include the image name, the performed image shift corrections in the *X* and *Y* directions (if activated), the grey levels and spread in grey levels in the images, and the segmentation limits used. This file also contains the number of identified cells in each image and the accumulated number of matched cells during the analyses.

`_Overall_summary.csv` - This is the most important results file for the regular user. Each row in the file summarises some of the common cell migration results for each image series. The measurements reported are either based on the averages over tracks (*n* equals number of valid tracks), averages over time (*n* equals number of times steps in the series) or averages over all cell movements (*n* equals all cell movements that are part of a valid track) (see upper part of Figure 3). Several common measurements such as mean velocity, directness, Forward Migration Index (FMIx and FMYy), accumulated distance, Euclidian distance, and Euclidian velocity are included. The statistical parameters SD (Standard Deviation and SEM (Standard Error of the Mean) are included in separate columns following each measured mean value. The file also lists the number of cells identified in the first and last images, as well as the total number of valid tracks for each image series. Videos downloaded from Incucyte S3 contain a video number and well coordinates (e.g., VID1234_B2_1.avi). CellTraxx will therefore search the video file names for any digits followed by an underscore and then a letter and a digit. These values will be written separately near the end of the `_Overall_summary.csv` file. This enables the user to easily sort the results based on video numbers or the positions in the 96-well plates (see last columns in Figure 3).

	Results based on:			Tracks	Tracks	Tracks	Tracks	Tracks	Tracks	Tracks	Tracks	Tracks	Tracks	Tracks	Tracks	Tracks	Tracks	Tracks	Tracks	Tracks
				Total number	Velocity	Velocity	Velocity	Directness	Directness	Directness	FMI X	FMI X	FMI X	FMI Y	FMI Y	FMI Y	Accumulated distance	Accumulated distance	Accumulated distance	Euclidian distance
First image		Last image		n	Mean	SD	SEM	Mean	SD	SEM	Mean	SD	SEM	Mean	SD	SEM	Mean	SD	SEM	Mean
Name	# cells	Name	# cells	[ ]	[um/min]	[um/min]	[um/min]	[ ]	[ ]	[ ]	[ ]	[ ]	[ ]	[ ]	[ ]	[ ]	[um]	[um]	[um]	[um]
VID959_B4_1_0000	311	VID959_B4_1_0084	416	442	0.40457	0.17592	0.00837	0.18912	0.12539	0.00596	0.12566	0.11479	0.00546	0.11275	0.09913	0.00472	253.87769	134.67008	6.4056	40.51211.

Tracks	Tracks	Tracks	Tracks	Tracks	Time steps	Time steps	Time steps	Time steps	All cell movements	All cell movements	All cell movements	All cell movements	Histogram	Histogram	Histogram	Histogram			
Euclidian distance	Euclidian distance	Euclidian velocity	Euclidian velocity	Euclidian velocity	Total number	Velocity	Velocity	Velocity	Total number	Velocity	Velocity	Velocity	Velocity	Velocity	Velocity	Velocity			
SD	SEM	Mean	SD	SEM	n	Mean	SD	SEM	n	Mean	SD	SEM	Mean	Mode	Median (Q2)	Upper quartile (Q3)	Video	Well coordinates	
[um]	[um]	[um/min]	[um/min]	[um/min]	[ ]	[um/min]	[um/min]	[um/min]	[ ]	[um/min]	[um/min]	[um/min]	[um/min]	[um/min]	[um/min]	[um/min]	number	Letter	Number
25.68737	1.22182	0.07177	0.05084	0.00242	84	0.39284	0.02118	0.00231	28604	0.3923	0.36296	0.00215	0.39229	0.11674	0.2603	0.50352	959	B	4

Figure 3. The overall summary file with the results from all videos that are analysed during the same run. Data from each video is listed in consecutive rows. In this example, only the top row of data is shown.

<u>CellTraxx settings files</u>: These are stored in the results folder for future reference and to allow repeating the same run with the same settings later if desired.

`celltraxx_bitmaps2process.txt` - This file contains the names of the videos in the order they were analysed as well as the first image number and last image number that was analysed.

`celltraxx_settings.txt` – A copy of the file `celltraxx_defaults.txt` containing the settings which were used during this run. Note that the copy has a different name ending with "settings" to distinguish it from the defaults file in the `C:\celltraxx_system\` folder. To repeat an earlier analysis – possibly with some minor changes - one could rename the `celltraxx_defaults.txt` file, copy the previously used `celltraxx_settings.txt` file into the `C:\celltraxx_system\` folder and then rename the file to `celltraxx_defaults.txt` before running CellTraxx again.

<u>Results files generated for each individual video</u>: In the following, `<VIDEONAME>` means the original name of each AVI video file without the extension (.avi).

`<VIDEONAME>_____0000_First_Image_Background_02_iterations.bmp` - This bitmap image shows the pixels used to calculate the background grey level by masking in black pixels dark and bright areas that were excluded. (See `segmentation limit` in *Advanced running*). If any non-masked pixels in this image contain cells, debris or other non-background items, the automatically determined segmentation limit may not be optimal.

`<VIDEONAME>__Diameter-histogram.csv` - This file includes the size distributions of candidate cells and identified cells. The number of data rows in the file is determined by the value of "`# bins in histogram`" in the `celltraxx_defaults.txt` file. The first column gives bin midpoints for the equivalent circular diameters. The second column shows the number of candidate cells (i.e., segmented regions) in each diameter bin. The third column counts the number of identified cells (i.e., candidate cells with diameters between "Smallest cell diameter" and "Largest cell diameter").

`<VIDEONAME>__Diameter-histogram.png` - This plot is generated from the corresponding `<VIDEONAME>__Diameter-histogram.csv` file by the ImageJ macro. The plot is useful for checking that the values of "Smallest cell diameter" and "Largest cell diameter" were correctly chosen. The "Smallest cell diameter" should lie near the left edge of the main peak in the plot, and not include much of the peak or shoulder of small debris if present. The "Largest cell diameter" should include the right-hand shoulder of the main peak and thus not exclude any large cells that clearly belong to the bell-shaped cell size distribution.

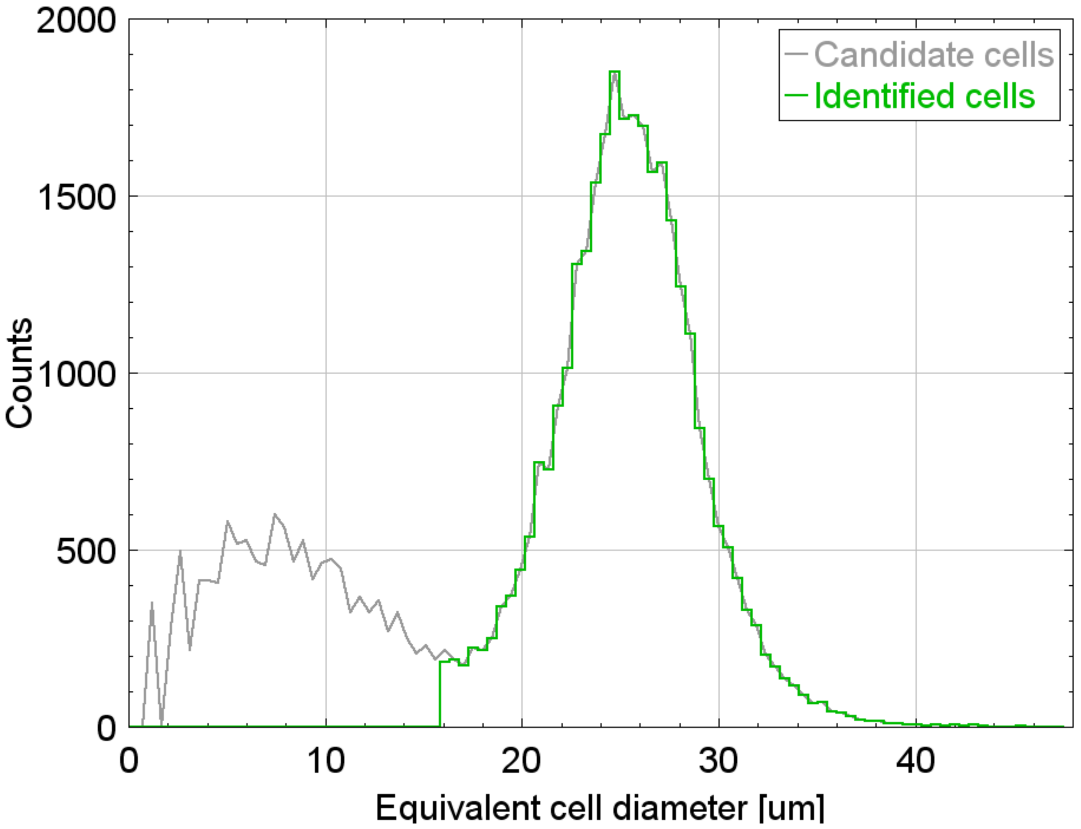

Figure 4. Two size distribution curves with number of cells plotted versus the equivalent circular diameter for all candidate cells (grey) and the identified cells (green). The identified cells are selected based on their size and are included in the analysis. The excluded, small candidate cells typically represent particles such as cell debris, dying cells or other types of noise.

`<VIDEONAME>__Image-shift-matrices.csv` - When image shift is activated, this file will include the series of matrices used to determine how much the current image should be shifted sideways to correct for any repositioning inaccuracies in the (X,Y) plane (see text above). These files may give hints of what went wrong in cases where the image shift correction did not work as expected, giving large sideways jumps in the results videos.

`<VIDEONAME>__Matched-cells.csv` - This file provides information as to which cell numbers have been matched for each track and at each time step.

`<VIDEONAME>__Position-data.csv` - This file includes a table with position and velocity data for each track at each time step (image). The data can be used to generate trajectory plots. Therefore, a blank line is inserted between each track so the tracks can easily be plotted individually.

`<VIDEONAME>__Position-data_Common-origin.png` - The cell trajectories plotted from a common origin. All valid tracks are included. Note that the scale of both axes (in µm) alters automatically with the endpoint of the most distant tracks, by rounding upwards to the nearest 100 µm. See Figure 5 for an example plot.

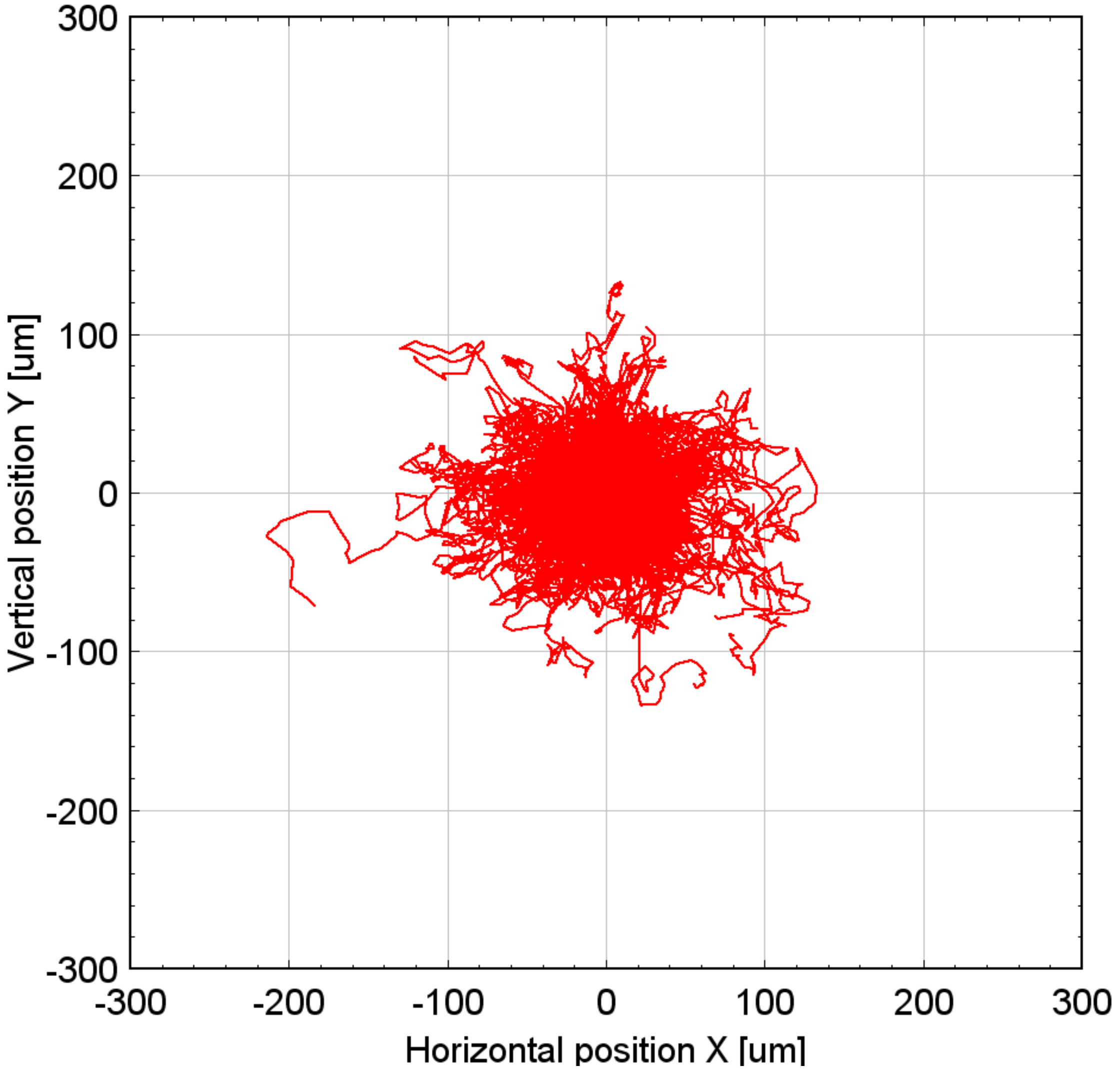

Figure 5. Cell trajectories plotted from a common origin.

`<VIDEONAME>__Position-data_Common-origin_Long-tracks-numbered.png` - The cell trajectories plotted from a common origin and with the track numbers displayed at the end of the longest tracks. All valid tracks are included. They are drawn with the same colour as in the valid track video. Note that the scale of the axis alters automatically with the length of the tracks. See Figure 6 for an example.

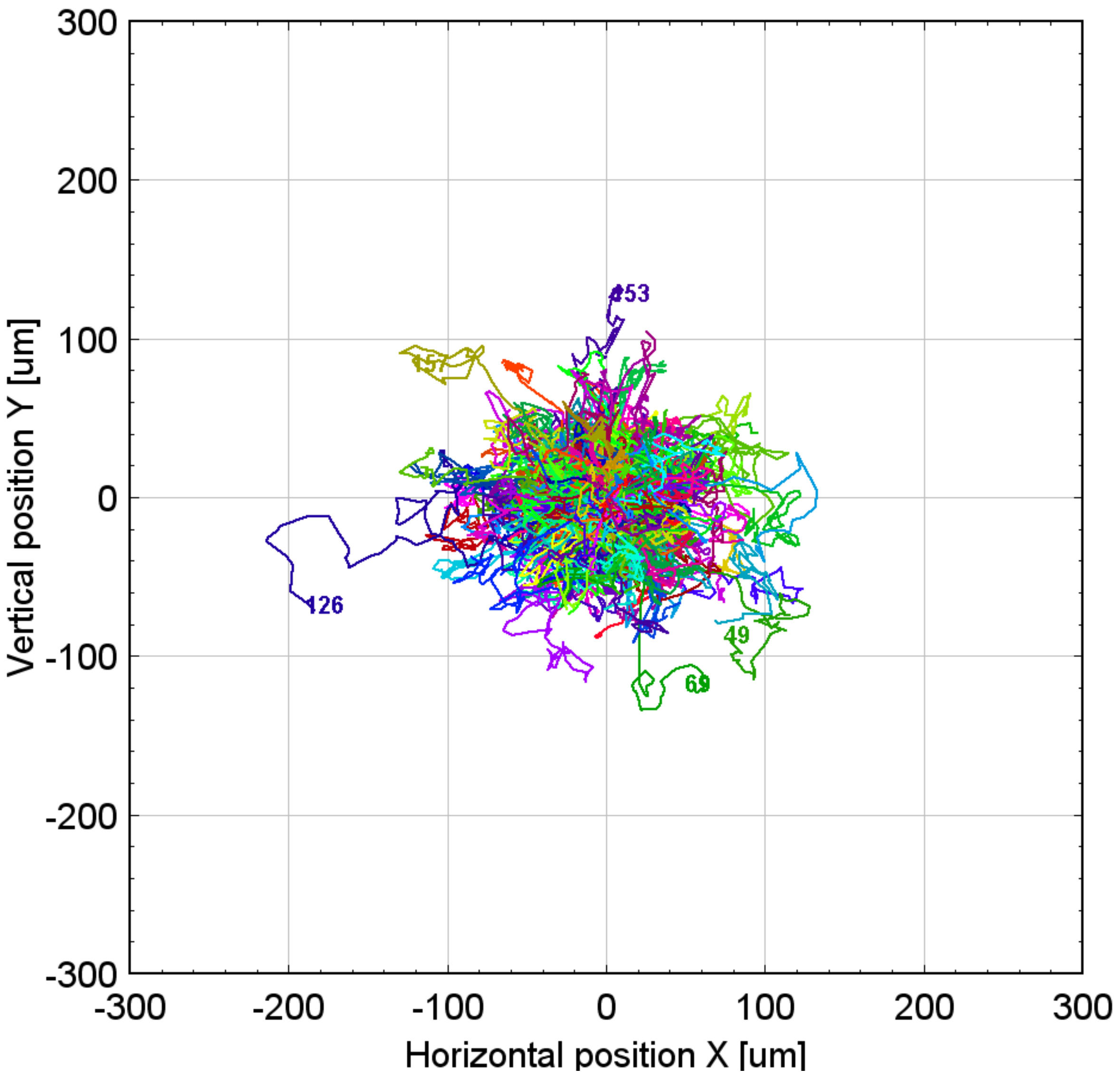

Figure 6. Cell trajectories plotted from a common origin with the longest track numbers indicated. The track colours are the same as in the valid track video.

`<VIDEONAME>__Position-data-as-in-image.png` – All valid cell tracks plotted as positioned in the image series. The colours are the same as in the valid track video. However, since the video shows only tracks where the cells are still present in the image, this plot will generally contain more tracks than even the last image in the valid track video. An example plot is given in Figure 7.

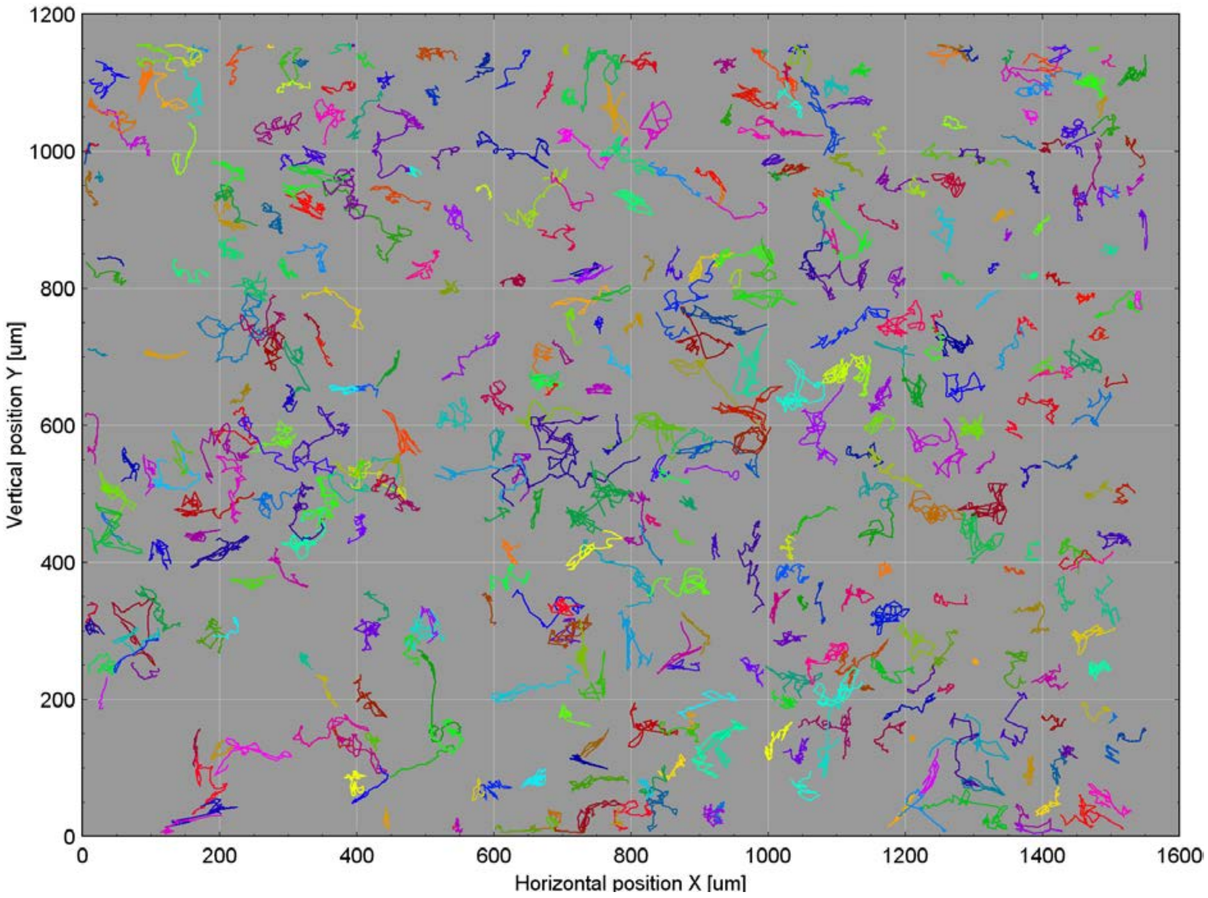

Figure 7. Valid cell trajectories plotted as positioned in the image series. The tracks are drawn with the same colours as in the valid track video.

`<VIDEONAME>__Position-data-as-in-image_Tracks-numbered.png` - The cell trajectories plotted as positioned in the image series. All valid tracks are included and are drawn with the same colours as in the valid track video. The track numbers, as given in the results CSV files, are drawn at the end point of each track. Figure 8 shows an example of such a plot.

`<VIDEONAME>__Track-data.csv` - This file contains an overview of the position and average velocity data for each track. Measurements listed are; The first and last image number of the track, (*X*,*Y*) position at the start and end of each track, Delta X and Y (change in coordinates from the start to the end point), Euclidian distance (length of a straight line from the start to the end of a track) and corresponding velocity, accumulated distance (total length of all cell movements for the track), *X* and *Y* forward migration index, angle (between the horizontal axis and a straight line from the start to the end of a track), number of time steps for the track, and finally the track mean velocity with SD and SEM.

`<VIDEONAME>__Velocity-histogram.csv` - This file provides the velocity distribution of all valid track cell movements in the video. The velocities are sorted into bins (see *Advanced running* for setting bin width) and counted. The first column gives the bin midpoints in units of µm/min. The second column contains the total number of counts at all time steps for each bin. The third column gives the position of a parabola fitted to the peak of

the histogram curve. The top point of the parabola is used for determining the velocity mode value (i.e., the most common velocity). Subsequent columns contain counts at all analysed time steps in case the user would like to study changes over time in the velocity distribution.

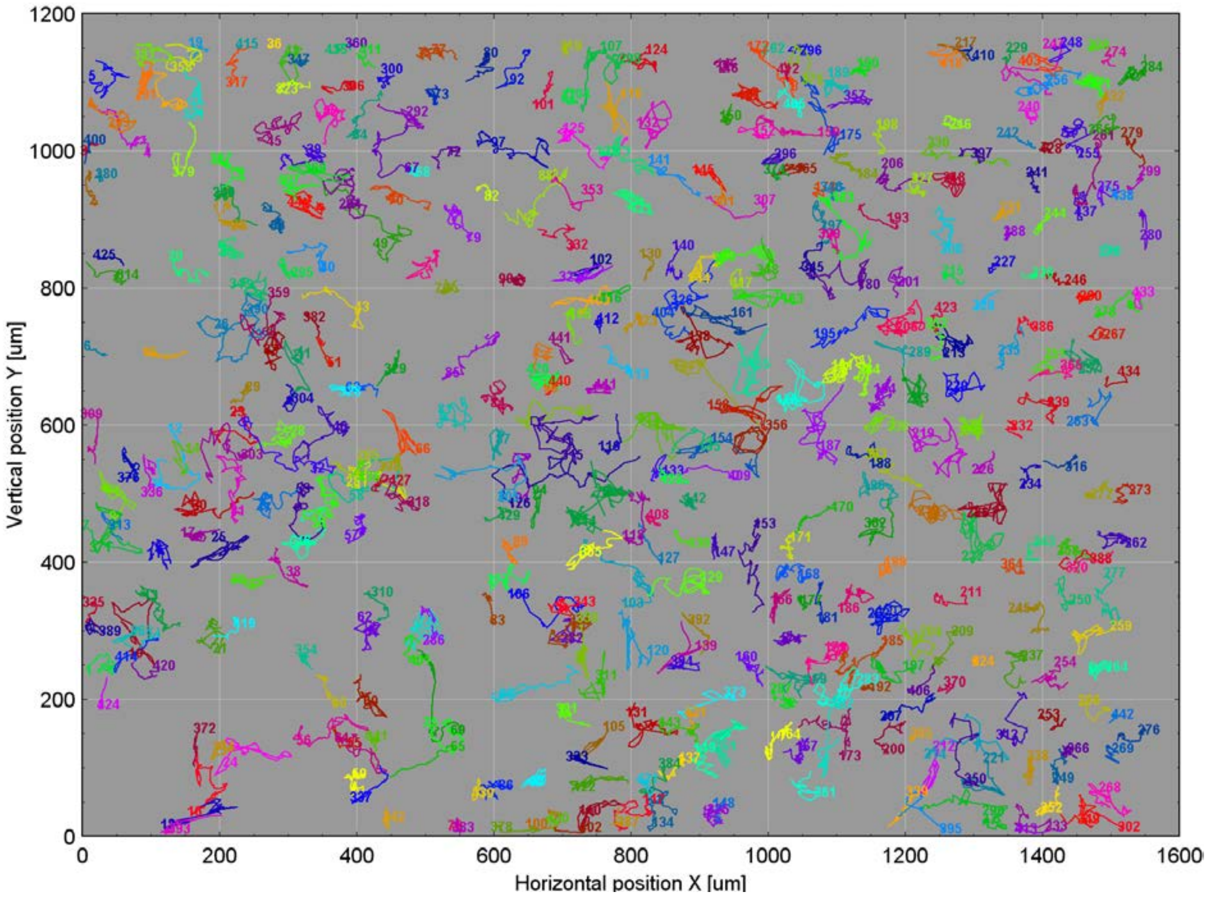

Figure 8. All valid cell tracks plotted at their respective locations in the video images. The track colours match those in the valid track video. The end points of all tracks are marked with their track numbers.

`<VIDEONAME>__Velocity-histogram.png` - This plot shows the cell velocity distribution for all cell movements, as given by the first two columns of the velocity histogram. The best fit parabola is drawn with a blue line. The parabola and velocity distribution curves are used by CellTraxx to calculate the mean, mode and median (Q2) velocity, as well as the 75th percentile (Q3) velocity. The *X*-axis ends at the user defined "Highest cell velocity". See Figure 9 for an example plot.

`<VIDEONAME>__Velocity-matrix.csv` – This comprehensive file contains the velocities for all valid cell movements and their averages calculated in three different ways. The tracks are stacked next to each other in columns and the time increases down each column. Above and to the left of the velocity matrix are average velocities with Standard Deviations (SD) and Standard Errors of the Mean (SEM), calculated for all movements, each track and each time step. Note that these three mean values are generally somewhat different. This is not due to errors in the calculations, but results from the different weighting of the numbers when averaging all cell movements, compared to when first averaging cell

movements within each track and then averaging over all valid tracks. The same goes for averaging over time. The fifth column, under the heading "Velocity sum / cell" gives for each image the length of the vector obtained when adding all the velocity vectors from all cells at this time step. Since cells generally move in random directions relative to each other, the "Velocity sum" vector is normally short with a length close to zero. However, if an image is shifted relative to the previous image (and image shift correction is not activated), all cells will get a common addition to their velocity vectors. This extra contribution will add up when the "Velocity sum" vector is calculated. Thus, a long vector will signify an image shift. The length of the "Velocity vector" is divided by the number of cells to get a measure of the image shift which is independent of the cell count. A plot of the "Velocity sum / cell" is therefore a good way to check if image shift correction should be turned on, in case there are many peaks above 0.1 µm/min in that plot. See also a simpler way to check this in the next paragraph.

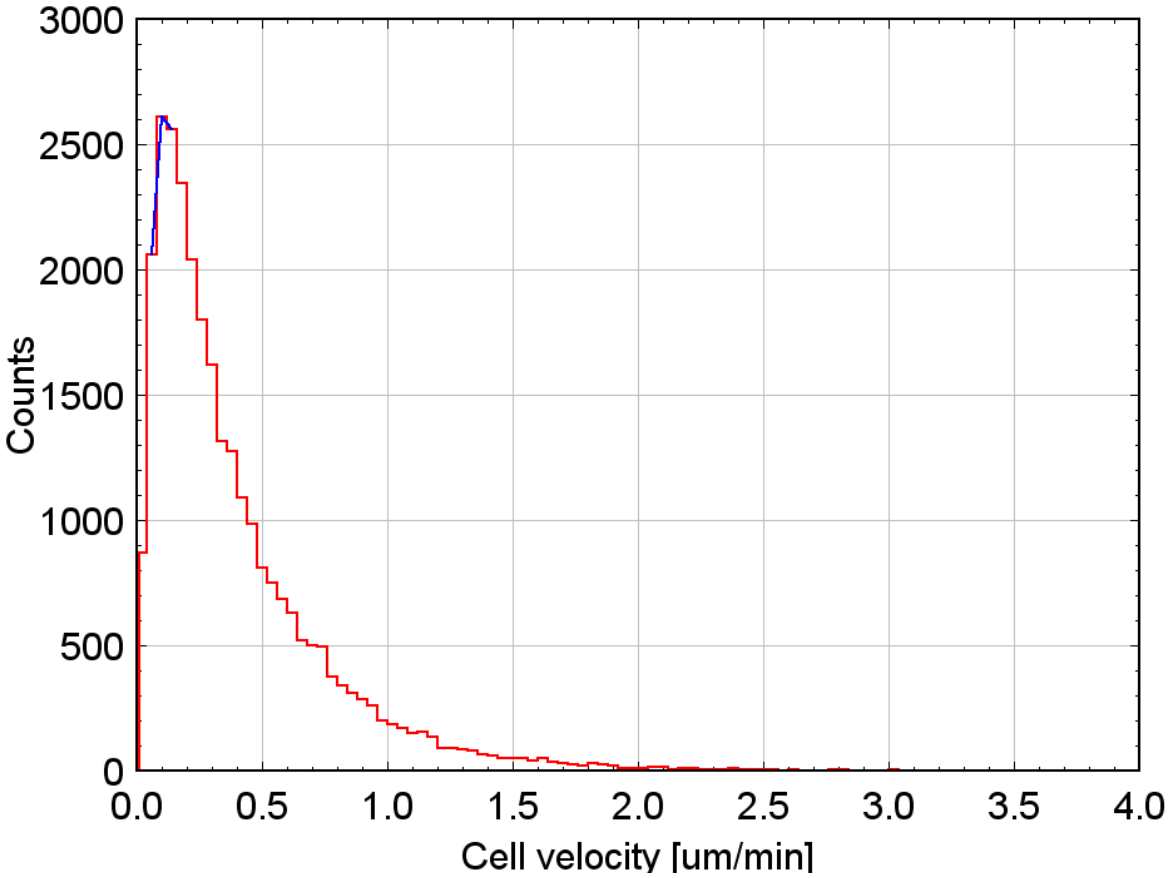

Figure 9. The velocity histogram (red) with a fitted parabola (blue).

`<VIDEONAME>__Velocity-vs-Time.png` - This plot shows the mean velocity versus time in hours. If few cells are tracked, the curve becomes noisier. Note that the length of the vertical axis is automatically adjusted to fit the measured velocities. Figure 10 shows an example plot.

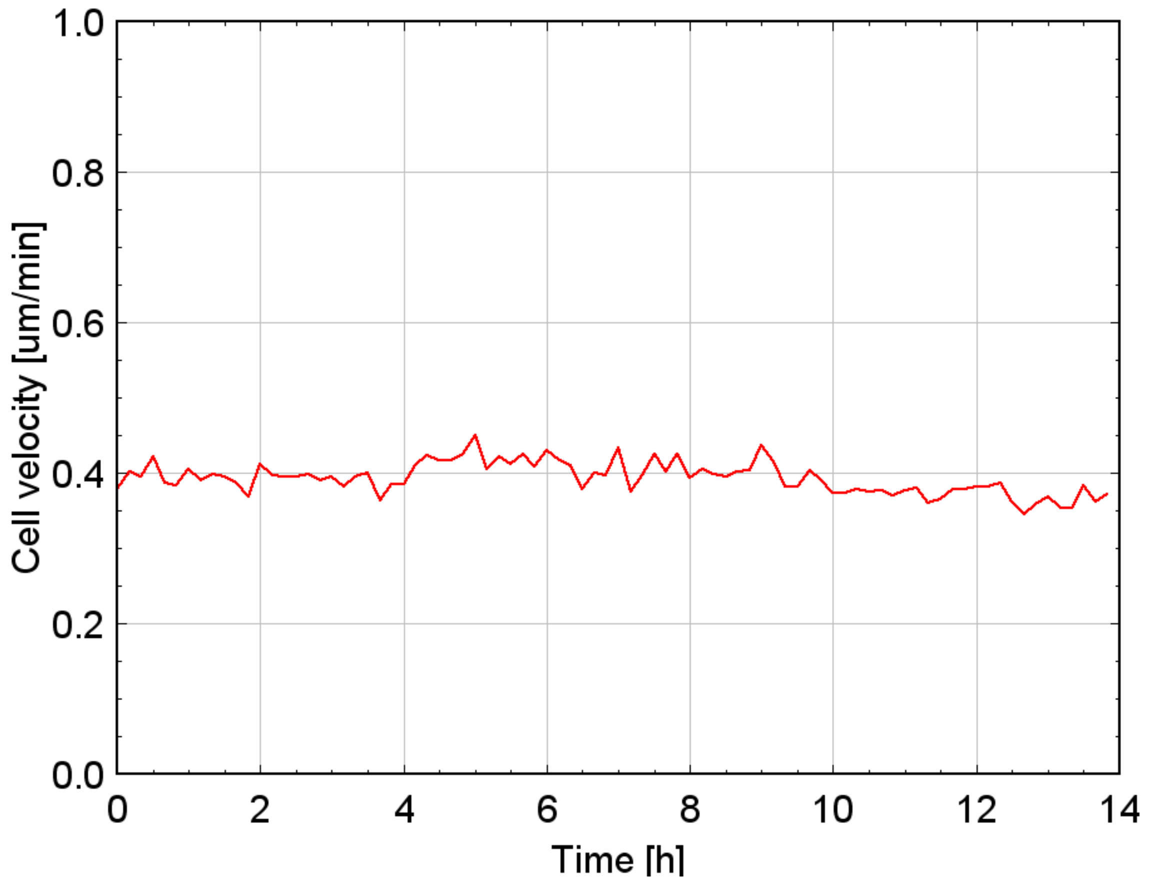

Figure 10. A plot showing the mean velocity (µm/min) versus time (hours).

Depending on the check boxes in the lower left corner of the settings window, three AVI videos can be generated by the ImageJ macro, based on bitmaps written by `celltraxx.exe`. Normally the bitmaps are deleted from the results sub-folder once the videos have been generated to save disk space.

`<VIDEONAME>__Video_Identified.avi` - Video showing identified cells outlined in different shades of green colour.

`<VIDEONAME>__Video_Matched.avi` - Video with all matched cells, including those with too short tracks to be used in the statistical calculations. Cell outlines and tracks are displayed in matching colours that differ from one track to the next.

`<VIDEONAME>__Video_Valid_Track.avi`- Video where only the valid tracks are included. The colour scheme is the same as for the Matched videos and the various track plots described above. Normally, only the valid track videos should be generated to save disk space.

Validation of the data:

- We recommend running at least three parallel wells (three replicates) for each condition in each experiment. The mean measurements of replicates are usually very similar and thus high variation between replicates implies something is wrong with either the

experiment or with the tracking. Examine your data carefully if you discover higher variation between samples than normal.

- Examine the videos with valid tracks to see if most cells are tracked, and whether the tracks are prematurely halted or unreal, large movements are allowed. If too large, unreal movements can be seen, decrease the value for "Highest cell velocity”. If long cell movements are lost i.e., a track is stopped when a cell makes a large movement, increase the value for "Highest cell velocity”. Also inspect the output file `<VIDEONAME>__Velocity-histogram.png` and check that the velocity distribution curve is not significantly cut at the right end of the horizontal axis. The horizontal axis should ideally end where the smoothed tail of the distribution curve reaches zero. If too much of the tail is cut off, the "Highest cell velocity" value should be increased. If the curve reaches zero long before the right-hand edge, this parameter can be reduced. (See Figure 9 where the "Highest cell velocity" is reasonable.)
- If many of the cells are not identified, improve the cell segmentation by adjusting the “Gaussian filter radius” (see *Running CellTraxx*, *Segmentation*, page 6) or “Segmentation limit” (see next point and *Advanced running*, page 26).
- Check the bitmap images which are generated to calculate the background grey levels and the automatic segmentation grey limits, (`<VIDEONAME>_____0000_First_ Image_Background_02_iterations.bmp`). If one of these images is dominated by black pixels and less than, 10 % of the area contains background pixels, CellTraxx may have too few pixels for the statistical analysis and the segmentation might be poor. This can happen if the cells are too dense or the image series has a noisy background, such as cells in a coated well (e.g., with collagen). In these cases, it might help to increase the segmentation limit (see *Advanced running,* page 26).
- If the number of cells identified in the last image is much smaller than the total number of valid tracks in that video, the settings should be re-evaluated. A high number of tracks compared to the final number of cells means that many tracks have stopped and re-started, indicating that the segmentation and/or tracking is not optimal.
- If an analysis contains too many short tracks, the settings should be improved. Check the velocity matrix file (see under *CellTraxx output,* page 17). The number of time steps for each track gives an indication. With good settings there should be some cells that are tracked through the entire video.

Wound healing mode:

In this mode, CellTraxx assumes that the wound (stripe where cells have been removed) is horizontal and nearly centered in the image. To tracks cells in a wound healing experiment, check the box for "Wound healing mode" in the CellTraxx Settings window in ImageJ. In the subsequent CellTraxx Tuning Image window, move the magenta frame which defines the crops margins so they cover the wound area plus the desired number of cells lying closest to the wound area, both above and below the wound. Since CellTraxx defines the middle of the wound based on the crop frame, try to center it well. Perform the rest of the tuning as normal. CellTraxx will now only track the cells closest to the wound as they move into the wound area. Note that, if there is significant drift in the images due to inaccaurate image repositioning and heavy image shift correction, one might experience that the cells at the

border of the original crop margin will appear as mirrored in the generated AVI video file. This is intentional and will not significantly influence the analysis.

Since the cells in a wound healing experiment are confluent, it might be difficult for CellTraxx to keep separate cells that come very close, often passing under or on top of each other. Therefore tracks might more frequently stop and start during wound helaing analysis. This might influence measurements like directionality and Forward Migration Index (FMI). This is important to keep in mind when analysing the results. Examine the tracking carefully and use the numbers with caution.

When wound healing mode is activated, CellTraxx will add additional directional measurement data in the rightmost columns of the `<VIDEONAME>__Velocity-matrix.csv` file. Use this file to extract wound healing results after the run is completed.

> *Tip: Note that for wound healing experiments, batch analysis of many videos in one run is not advicable since the wound area will typically vary from image series to image series and thus the crop margins to define the wound area will have to be set separately for each file.*

Cell counting (e.g., proliferation experiments):

Since CellTraxx segments and identifies cells in phase contrast images, the program can also be used to count cells e.g. in proliferation experiments. For this purpose, run CellTraxx as described above with a few exceptions as outlined here: CellTraxx has a feature to recognize cells based on the previous image. This feature is normally kept "on" for tracking experiments. However, for cell counting this feature is not needed and moreover, could in some cases negatively interfere with the identification of cells. To turn the feature off, open the file `celltraxx_defaults.txt` in the `C:\celltraxx_system\` folder. Change the "yes" to "no" after the phrase "Keep cells from previous image" near the bottom of the parameter list. (See *Advancerd running,* page 26). In the settings window, the parameters "Highest cell velocity" and "Shortest cell track" could be set to any number (3 or more for "Shortest cell track"), but feel free to keep the defaults. Also, check the box in front of "Generate videos with identified cells" in the settings window (See *Running CellTraxx, Output*, page 8). The cell counts per image in all image series analysed are summarized in the output file, `_Identified_cells_summary.csv`. For easy sorting of the data (for example parallel samples in the same row/column of a 96 well plate), the VID number and the well coordinate letter and number are included separately in columns 2, 3 and 4, respectively, if the Incucyte S3 name convention has been used. To organize the data in columns instead of rows, simply select all, copy and then paste special with transpose into a new spreadsheet.

Troubleshooting:

- To stop celltraxx.exe while running: Select the celltraxx.exe window and press the keys **[Ctrl]**+**[C]** or **[Ctrl]**+**[Break]**.

- Check for any error messages in the file `celltraxx_error_messages.txt` in the folder `C:\celltraxx_system\`.

If the software will not run:

- Download the latest version of ImageJ or Fiji. Version 1.53t or newer can be used.
- Check that the software is stored exactly as described in *Preparations, Installing the software*, page 2.
- Check that there is a folder called `X:\celltraxx_data\` containing the AVI files to be analysed, and where `X` is a valid disk drive letter specified in the CellTraxx Settings window after "Disk drive letter for data folder…".
- Check that there are no spaces in the names of the AVI files or the folders.
- If you cannot find the **celltraxx_imagej_macro** in the list of ImageJ macros under the Plugins menu, it must be installed again. See *Running CellTraxx, 1. Image J*, page 4.
- Make sure that the version number specified in the CellTraxx Settings window in ImageJ, which is the same as given in the `celltraxx_defaults.txt` file, matches the version number which is coded inside `celltraxx.exe`. The ImageJ macro will display an error message if the numbers do not match.
- If the AVI files are compressed or not in the right format, download them again from Incucyte as described under *Preparations*, *Image files*, page 3.
- Do not close the `ImageJ Log` window or the command window while running (unless the execution was started by mistake).
- Do not open the output files before the run is finished. If many videos were analysed, it can take several minutes for ImageJ to post process the results and generate output videos. Wait until the ImageJ Log window displays "Finished processing all .avi videos in the folder…".
- Error in CellTraxx: The number of cells has reached the maximum size of the arrays which store cell data. An error message appears if the number of cells in an image exceeds 6000. Increase the crop margins to analyse a smaller selection if this occurs.
- If any of the files in the `C:\celltraxx_system\` folder, or any of the new output files, are open in another program, CellTraxx may halt execution and give an error message. Check and close the file(s) and rerun CellTraxx. If CellTraxx is not running normally after closing the files, check that the files are not corrupt.
- Right-click the `celltraxx.exe` icon and select "Run as administrator" to see if this causes a command window with output information to appear. If so, it might help to log in as administrator while running CellTraxx or to relax the **Properties** > **Security settings** of the `C:\celltraxx_system\` folder and/or `celltraxx.exe` file. If the ImageJ macro gives file access error messages, it might likewise be necessary to run ImageJ as administrator.
- The folder `\alternative_celltraxx_versions\` which was downloaded from GitHub (See *Installing the software*, page 2) contains executables compiled for PCs that have older redistributables (see next item). Copy the executables to `C:\celltraxx_system\` and double-click the icon to see if one of them will run. If so, rename the working program to `celltraxx.exe`.
- If not running Windows, compile the C source code `celltraxx_v46_2023-10-07.c` available in the folder `\alternative_celltraxx_versions\`.
- Check that the required Microsoft Visual C++ Redistributable Package is installed: Open **Settings** on the **Start Menu** and click **Apps**. In the list of installed programs, search for *Microsoft Visual C++ 2015-2019 Redistributable (x64)*. For older Windows versions go to **Control panel** > **Programs and features**. If not installed, go to: https://learn.microsoft.com/en-us/cpp/windows/latest-supported-vc-redist?view=msvc-170#visual-studio-2015-2017-2019-and-2022 to download and install the missing

redistributable.

The interactive tuning tool is not working:

- The AVI file(s) must contain at least 3 images.
- Shortest cell track cannot be smaller than 3.
- The magenta cropping frame is lost: The magenta lines that enclose the area to analyse may disappear if the user clicks on the image outside the cropping frame. To regain the magenta frame simply use the *Rectangle tool* in ImageJ and draw a new rectangle. The cropping frame will reappear after you click **[Update]**, **[First]**, **[Middle]** or **[Last]**.
- The CellTraxx Tuning Image may appear cropped on the screen or shows only part of the button row or image if your screen resolution is too small to display the tuning image with 100 % zoom. If so, activate the tuning image and press **[-]** to reduce the image magnification until the full tuning image is visible. You may zoom back to view regions of special interest by pointing the cursor on that location and pressing **[+]**.

Image shift correction is not working:

- If there are still large jumps in a video even when the image shift correction is activated, CellTraxx was unable to correct the image shift. This may occur if the shift is more than 100 pixels or if some large object is moving across the images. In the latter case, consider using a smaller cropping frame which avoids the disturbance. In the former case, it is always possible to increase SHIFTXY in the program code and recompile if you have access to a C/C++ compiler.

Segmentation is not working properly:

- If automated segmentation does not find the cells or selects large background areas, the user can set a negative value for the `Segmentation limit`, typically between -0.5 and -2. Then the automated limit is overruled, and the grey level limit used by CellTraxx for segmentation will be the *mean grey value* + `Segmentation limit` * *the standard deviation of the grey levels in the image.* A more negative value will cause smaller regions to be selected. Do not forget to set the Segmentation limit back to the default value of 1.5 once the troublesome image series has been analysed.

Advanced running

Advanced settings

The settings used in the previous run are read by CellTraxx from the file; `celltraxx_defaults.txt` in the `C:\celltraxx_system\` folder. The most common user-defined parameters can be adjusted in the "CellTraxx Settings" window which appears when the ImageJ macro is executed. To keep this window reasonably simple, not all CellTraxx parameters are shown in the settings window. The omitted parameters will normally not need to be adjusted. But in certain cases (for example if the background is noisy as in the collagen coated dishes) they can be changed manually by editing the `celltraxx_defaults.txt` text file. Make sure to never change any of the keywords in

the left-hand column of this file. Otherwise CellTraxx may stop running since it cannot recognize the text when searching for specific parameters. Also note that adjustment of parameters that are later done in the ImageJ "CellTraxx Settings" window will overwrite those in the `celltraxx_defaults.txt` file. The following list includes a description of the adjustable parameters in `celltraxx_defaults.txt`. Parameters that have already been described under *Running CellTraxx*, pages 4-8 are left without comments in the list below:

`Wound healing mode`

`Perform flat field correction`

`Perform image shift correction [um]`

`Perform interactive tuning`

`Version`

`Track smoothing iterations`

`Results folder drive letter`

`Results folder name` - gives the full name of the results folder under `X:\celltraxx_data\` where the next results will be stored. The last part of this name will automatically be changed by the ImageJ macro if diameters or the filter size are changed in the settings window or during tuning.

`First part of folder name`

`Pixel size [um]`

`Gaussian filter radius [um]`

`Smallest cell diameter [um]`

`Largest  cell diameter [um]`

`Cutting  cell diameter [um]`

`Top    crop margin [pixels]`

`Bottom crop margin [pixels]`

`Left   crop margin [pixels]`

`Right  crop margin [pixels]`

`Time between images   [min]`

`Highest cell velocity [um/min]`

`Shortest cell track   [images]`

`First image number`

`Last  image number`

`Image number increment`

`Make identified cell videos`

`Make matched cell videos`

`Make valid track videos`

`Tracking dot diameter [um]`

`Valid track image contrast`

`Scale bar color`

`Draw cell outline` - When set to "`yes`", CellTraxx draws a colour-coded outline around the segmented parts of cells in videos with matched cells. This parameter has no effect on other types of output videos.

`Draw cell track line` - When set to "`yes`", CellTraxx includes in videos with matched cells and valid tracks a colour-coded line which follows the tracked path of currently visible, tracked cells.

The following eight settings beginning with "`Write`" are set to "`no`" by default. They may be changed to "`yes`" during troubleshooting if CellTraxx has problems analysing a video correctly. Then the desired bitmap images will be present in the results folder when CellTraxx is finished. It may help to look at images from the various stages of analysis to better understand where the problems arise.

`Write mirror margin images` – If set to "`yes`", CellTraxx makes bitmaps where the pixels inside the cropping frame have been mirrored outside the frame with a margin 100 pixels wide on all sides. This image is used by CellTraxx when running the Gaussian filter to allow fast and efficient filtering without edge effects.

`Write shifted images` - CellTraxx will always write shifted bitmaps to the current results folder and read them back when making the valid track videos. If this parameter is set to "`yes`", the shifted bitmaps will not be deleted after the videos have been made. Shifting is done only inside the cropping frame. Pixels outside the frame are unchanged. If the required image shift is large, the mirrored parts of the image may be easily visible near the edges of the cropping frame.

`Write gaussian smoothed images` - If set to "`yes`", CellTraxx makes bitmaps which show the effect of Gaussian smoothing within the cropping frame. The stored bitmaps have only 256 grey levels, but in the computer memory the smoothed image has decimal grey levels which allows segmentation at non-integer grey level values.

`Write segmented cell images` - If set to "`yes`", CellTraxx makes bitmaps where all segmented, candidate cell regions are outlined in yellow.

`Write cut cell images` - If set to "`yes`", CellTraxx makes bitmaps where candidate cell regions are outlined in green and cutting lines are shown in blue. Cyan coloured pixels on the periphery of cell regions show candidate points for cutting but where the region is not concave. Magenta pixels show concave candidate points. CellTraxx does not always cut

between two opposing magenta pixels, for instance if their separation is larger than the `Cutting cell diameter`.

`Write identified cell images` - If set to "`yes`", CellTraxx makes bitmaps where regions that are accepted as cells with the current settings are outlined in various shades of green. The shading makes it easier to see if adjacent cells have been correctly separated or not. These images can be useful for checking the settings in proliferation experiments.

`Write matched cell images` - If set to "`yes`", CellTraxx makes bitmaps where identified cells that are also matched with cells in the previous image are marked with tracking dots, outlines and/or track lines, depending on the given settings. The difference between these images and the valid track images is that the matched images show also tracks that are shorter than `Shortest cell track`.

`Write valid track images` - If set to "`yes`", CellTraxx makes bitmaps where only cells in tracks with length equal to or longer than `Shortest cell track` are marked. Note that only tracks where the cell is currently still being tracked are included. For instance, tracks for cells that have moved outside the cropping frame are not shown.

`Keep cells from previous image` - This parameter should normally be set to "`yes`" since it generally improves the tracking by using the location of identified cells in the previous image. However, some cells may get straight-lined edges if they lie close to other cells. Only use "`no`" in special cases such as cell counting for proliferation experiments or where tests show that this gives fewer tracking artefacts.

`Segmentation limit [SDs]` - By default the segmentation limit should be kept at 1.5 standard deviations (SDs). This will in most cases result in a good segmentation of cells from the background. The segmentation limit determines how sensitive the masking of dark and bright pixels should be before CellTraxx analyses the cell-free background grey level to find the "Automatic grey limit" value. A larger `Segmentation limit` will mask fewer pixels and leave more background pixels for CellTraxx to analyse. This can be checked in the output images `<VIDEONAME>_____0000_First_Image_Background_02_iterations.bmp`. Too many masked (black) pixels in these images may result in poorer segmentation (see Figure 11). This may happen when the background noise is high (e.g., cells in collagen). Increasing the segmentation limit from 1.5 to 2 might improve the automatic segmentation in such cases. The black pixels should cover all cells, bubbles and debris, but not mask normal background regions free from cells.

If automated segmentation does not find the cells or selects large background areas, the user can set a negative value for the `Segmentation limit`, typically between -0.5 and -2. Then the automated limit is overruled, and the grey level limit used by CellTraxx for segmentation will be the *mean grey value* + `Segmentation limit` * *the standard deviation of the grey levels in the image*. A more negative value will cause smaller regions to be selected. Do not forget to set the `Segmentation limit` back to the default value of 1.5 once the troublesome image series has been analysed.

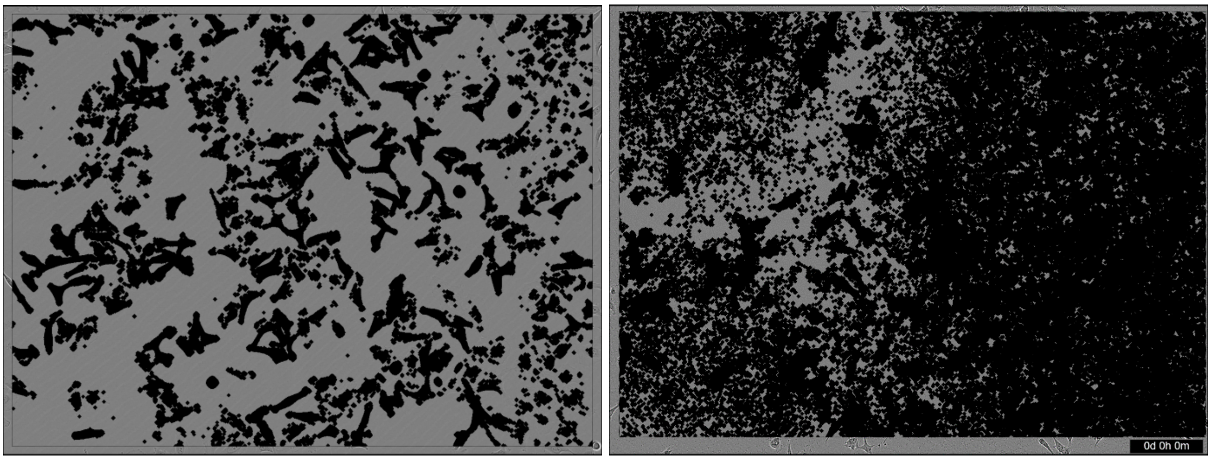

Figure 11. Examples of the effect of the `Segmentation limit` on the output image `<VIDEONAME>_____0000_First_Image_Background_02_iterations.bmp` which is stored in all results folders. The images are taken from a run with a good segmentation limit (left) and poor segmentation (right). In the latter example, increasing the `Segmentation limit` will improve the segmentation.

`# bins in histogram` - This parameter determines the number of rows in the `<VIDEONAME>__Diameter_histogram.csv` and the `<VIDEONAME>__Velocity_histogram.csv` output files. The default number of bins is 100, which works well in most cases.

`Tuning image code` - A parameter which allows the ImageJ macro to tell `celltraxx.exe` if the first, middle or last image shall be analysed during interactive tuning. When running celltraxx.exe without the ImageJ macro (see next paragraph), the `Tuning image code` should be 0 (zero).

Running CellTraxx without ImageJ

It is also possible to run CellTraxx without the ImageJ macro. All files referred to below should be present in the `C:\celltraxx_system\` folder. If all parameters in the above-described file `celltraxx_defaults.txt` are set correctly, and the desired video names with correct image numbers to analyse are included in the file `celltraxx_bitmaps2process.txt`, one can simply double-click the icon for `celltraxx.exe`. The command window will open and report the progress of the analysis in the usual way. However, since the ImageJ macro is not activated, there will be no results summary plots in the form of PNG images and no AVI videos automatically stored in the results folder. In case celltraxx.exe aborts with an error, the error message can be found in the file `celltraxx_error_messages.txt`, which otherwise contains only the two words "NO ERRORS". Running CellTraxx in this way can be convenient when repeating some previous analysis that does not require tuning and where only the output CSV files are needed.
