# Supplementary material for: Automated tracking of cell migration in phase contrast images with CellTraxx
Source: Sci Rep. 2023 Dec 27;13:22982. doi: 10.1038/s41598-023-50227-9 (PMC10752880; doi:10.1038/s41598-023-50227-9)
Supplement: Supplementary file 21 — Supplementary Information 2. [file 41598_2023_50227_MOESM21_ESM.pdf]

Description of the CellTraxx functionality and algorithms

The CellTraxx ImageJ Macro has the following features and does the following actions:

1) Will process all AVI files available in the data directory. Thus, it is not necessary to name the input files explicitly.
2) Converts AVI video files to individual bitmap images that can be read by celltraxx.exe using the built-in functionality in ImageJ. To save time, this is only done during the first run and is not repeated if such bitmaps already exist in the data directory.
3) Performs a pseudo flat field correction if selected by the user. The macro uses the built-in "Z Project" feature of ImageJ to find the median grey level at each pixel in the image. The whole video is divided by the background image and re-scaled to give an 8-bit video with an even background grey level. For videos with a low cell density and where all the cells move around so all pixels are free from cells more than half the time, the median grey level will represent the image background without cells everywhere. This functionality is therefore applicable only under such conditions but can be useful if a real flat field correction was not done on the original video. A reasonably homogeneous background is required for the global thresholding used by celltraxx.exe to function properly.
4) Shows the user the so-called "tuning window" where the segmentation result of running celltraxx.exe on the first image of the first video is shown. By clicking on buttons on the image, the user can interactively change the Gaussian filter radius, the min and max size for an accepted cell, and the diameter which governs the special cutting routine (see item H below).

When the user determines that the settings are good and clicks the [Run] button in the tuning window, the macro saves the current settings to a text file and runs celltraxx.exe which reads the settings file and sequentially processes the bitmaps. After celltraxx.exe has finished analysing all images in all available AVI videos, the ImageJ macro continues and does the following post processing:

5) Reads the bitmap output images from cellttraxx.exe and saves the user-selected types of videos as non-compressed AVI files.
6) Reads some of the results CSV files generated by celltraxx.exe for each original video and uses the plotting functionality in ImageJ to generate summary plots that are saved as PNG images. In this way the following plots are made: Velocity histogram, mean velocity versus time, tracks plotted from common origin (with and without numbers for the longest tracks), tracks plotted at the same location as in the image and with the same colour coding as in the results videos, and finally the cell diameter histogram showing the sizes of candidate cells and accepted cells as two separate curves.

The CellTraxx core software was written in C and compiled by Microsoft Visual Studio as a console application. The executable celltraxx.exe does the following:

A) Reads the newest default settings file.
B) Reads and processes all bitmap images in sequence, one image at the time such that it can handle both long videos and many separate videos without memory overflow.

C) **Crops the image** to the user-selected crop margins. To minimize the execution time during the subsequent image alignment and Gaussian filtering, the images are shifted 100 pixels to the right and upwards in the floating-point matrix where the grey level data are now stored. This allows the new image to be shifted up to 100 pixels in each direction when trying to find the best match with the previous image (see item D below) without risking the array indices to be out of bounds. Likewise, the Gaussian filter weighting matrix (see E) can be moved across the image with no array index checking since it has already been verified that the half-width of the weighting matrix is less than 100 pixels. To ensure that the Gaussian filter has realistic pixel data to work on beyond the cropping borders, the image is mirrored at the crop margins. First, the 100 pixels above the bottom crop margin are mirrored down to the bottom of the expanded image matrix. Then, the 100 pixels below the top crop margin are mirrored upwards. Finally, the 100 pixels right and left of their respective crop margins are mirrored sideways to give a shifted and mirrored image which is 200 pixels wider and higher than the selected crop margins. Since the matrix which holds the expanded image contains floating-point values, the subsequent sideways shifting and filtering can be done with decimal grey level values. We thereby avoid round-off errors. It also means that we can use an essentially continuous grey scale where the normal values for black (0) and white (255) still apply. The floating-point image matrix is thus an alternative to a 16-bit image.

D) **Lateral shifting** of a new image to match the previous image if "Perform image shift correction" was selected: The image alignment is done by calculating the average difference in grey level values for all overlapping pixels when the current and previous image are shifted in a systematic pattern. The goal is to find the shift vector which gives the smallest average grey level difference since it represents the shift where the images are most similar. The initial try is done using a shift vector [*i*,*j*] which gave the best fit for the previous image pair. This saves execution time if images drift slowly and systematically through the video. The average grey level difference value is placed at position [*i*,*j*] in a separate shift vector matrix. To further save execution time, only 24 additional shift vectors are tried in the first search for the smallest average difference in grey level. Celltraxx.exe checks all the eight neighbouring shift vectors (with distance 1 pixel laterally or diagonally), then the corresponding eight vectors with distance 3 pixels laterally and diagonally, and finally the eight vectors with 5 pixels separation. Our current experience is that this search is wide enough to not mistake a small local minimum for the global minimum in the grey level difference values of the shift vector matrix. If the current shift vector [*i*,*j*] gives the smallest grey level difference among all tested shift vectors, the search is terminated. If not, the search is repeated but now centred on that shift vector [*m*,*n*] which gave the currently lowest grey level difference. These iterations continue until a minimum value is found, assumed to be the global minimum which gives the correct image shift. To further improve the quality of the shift and the smoothness of the resulting, shifted videos, a sub-pixel shift is performed. By fitting parabolas to the minimum point of the shift vector matrix and the nearest neighbours in the x and y directions, the fractional shift vector [*f*,*g*] in floating-point values is calculated. The shifting of the current image is done by re-mapping using linear interpolation between the four neighbouring grey level values of the decimal shift vector. See the code in celltraxx.c available on GitHub for how the program selects two triangles that contain the nearest neighbour points and does the interpolation.

E) **Gaussian smoothing** is done by first generating a two-dimensional weighting matrix based on the selected Gaussian filter radius. The matrix extends laterally for as many pixels as needed for the weight to reach 1 % of its maximum value at the user-selected radius. The weights of the matrix are normalized to make their sum equal to 1. The filtering is done in the normal way by letting the centre of this matrix run across the image to calculate the weighted sum of the pixel grey values which then becomes the filtered grey value of the given pixel. As mentioned under item C), thanks to the 100-pixel image shift margin and the check that the weighting matrix half-size is always smaller than this margin, the smoothing calculation with a quadruple for-loop can be run fast without array index checking. Still, the Gaussian smoothing step is one of the main factors that slow down the execution speed of celltraxx.exe. One can notice that each image is processed more slowly when the Gaussian filter radius is large. The rationale for using a large radius Gaussian filter is that phase contrast images from the Incucyte S3 microscope generally give cells that are darker than the background, often with several particularly dark spots in the cell nuclei. In some cases, the phase contrast makes the outer edges of the cells brighter than the background. This is useful for separating nearby cells. After applying a suitably sized Gaussian smoothing filter to the image, we therefore end up with dark regions at the location of cell nuclei and brighter "walls" separating individual cells. The mirroring of the image along the cropping frame makes the smoothing function of celltraxx.exe less sensitive to the pixel values along the frame edges and corners compared to the built-in Gaussian blur function in ImageJ [https://imagej.nih.gov/ij/developer/api/ij/ij/plugin/filter/GaussianBlur.html].

F) **Segmentation** of candidate cell regions is done by marking all pixels in the smoothed image that are darker than a global threshold value. The threshold is a floating-point number. This allows distinguishing fine grey scale nuances from one another. Celltraxx.exe is designed to analyse hundreds or even thousands of videos with no user intervention after the initial parameters have been determined based on the first image of the first video, assumed to be representative of the remaining videos. Therefore, celltraxx.exe needs a particularly robust algorithm to determine the correct grey level threshold for each video. After encountering a video where an air bubble covered part of the image, giving some large, dark, concentric rings surrounding a bright circle, the following algorithm was developed to handle also such cases: Since the smoothed image contains dark cells on a brighter background, the basic idea is to find the grey level of the darkest pixel in the background. Anything darker is likely to be a smeared-out cell nucleus. The grey level analysis is done only on the first image in each video since this generally has fewest cells and thereby most background pixels available. To determine which pixels that belong to the background – and to be able to handle anomalies like bubbles – celltraxx.exe calculates the average grey level and the grey level standard deviation of the image before Gaussian smoothing. An image mask is then made which excludes all pixels that are darker or brighter than *Mean_Grey_level* ± *Segmentation_Limit* * *Grey_Level_Standard_Deviation*. The variable *Segmentation_Limit* may be changed by advanced users by editing the celltraxx_defaults.txt file, but is set to 1.5 as default. This mask generally eliminates dark cell centres, and anomalies like bubbles. For images with particularly noisy background, the *Segmentation_Limit* may have to be increased to prevent that too many pixels are masked (see Figure 8 in the CellTraxx User Manual). To be on the safe side, celltraxx.exe expands the masked regions by four pixels using a simple, four-connected dilate algorithm. This is typically enough to fully mask cell centres and anomalies. To allow the user to check if the masking is reasonable, the original image

with masked pixels in black is written to the current results folder where the filename starts with the name of the video and ends with "…_First_Image_Background_02_iterations.bmp". A grey level histogram is now generated from the non-masked background pixels of the smoothed image. The histogram contains all grey levels between 0 and 255 in steps of 1. The peak of the histogram is determined to find the most common grey level of the background pixels. Then another histogram is made which includes only pixels that are within ± 20 grey levels on each side of the most common grey level. This histogram has a bin width of 0.05 grey levels, thus gives a high-resolution version of the peak in the smoothed background grey level distribution. This peak typically has the shape of a Gaussian function, probably because the image noise of an even background has a Gaussian distribution, even after the image has been smoothed. Since this new histogram gives the number of pixels as a function of grey level with a resolution of 0.05, we could in principle follow the left shoulder of the Gaussian peak until it had sunk to a count of 1, take the corresponding decimal grey level value as the darkest pixel in the background and use this as the segmentation limit. However, it is risky to use the slim tail of an exponentially falling curve to find the grey level limit. Such a method would be prone to statistical fluctuations in the counting statistics and would not be very robust. Instead, celltraxx.exe uses curve fitting to the high-resolution histogram to find a better grey level limit. The curve fitting is done by first taking the logarithm of the histogram curve and then normalizing it so the highest point has value 1. Since the logarithm of a Gaussian is a parabola, it was fast and easy to use our self-made least squares parabola fitting routine to match the transformed histogram peak. (This way of fitting a Gaussian was proposed by Caruana already in 1986[1]].) Since it is the darker half of the peak which is most important to fit, the parabola fitting is done over the interval where the normalized, logarithmic values reach -1.5 on the left side and -0.5 on the right side of the most common grey value. This interval corresponds to about 1/30th and 1/3rd of the maximum peak counts, respectively. Once the parabola coefficients are found, we have a smooth curve which can be extrapolated to any desired grey level. Instead of extrapolating to the value which represents the very darkest pixel, celltraxx.exe uses the slightly brighter limit where 0.1 % of the background pixels are darker than this value. This is simply done by calculating at which grey level the parabola reaches -3 on the left (dark) side. The decimal grey level value which satisfies this criterion is used as the upper grey level limit for segmentation of cell candidate regions for all images in that video. The segmented image is a 16-bit integer matrix where all pixels darker than the decimal grey level limit in the smoothed image are coded as 1 and the remaining pixels as 255. The choice of a 16-bit matrix was made to later allow coding identified cells individually in the same matrix by using thousands of different integer values if needed. Celltraxx.exe writes a CSV file to the current results folder which gives the values of the high-resolution histogram for the smoothed image, the unmasked background, the normalized logarithm of the counts and the fitted parabola. The file is named "_Grey_level_curves_for_first_images.csv" and includes the data for the first image of all videos analysed in that run. If the user suspects that the automatic segmentation did not work well for a certain video, it is easy to plot all the curves and look for anomalies.

This segmentation procedure is more complex than, and different from, the commonly used, automatic global thresholding algorithms featured in ImageJ. This level of complexity and robustness is necessary to handle Incucyte S3 images, and the algorithm works quite well for such datasets.

G) **Filling holes** in all the cell candidate regions is done by a flood filling algorithm which begins at the first available background pixels near all four corners of the cropping frame. Using the four corners makes it very likely that all background pixels are found, even if the cell density is high and cell clustering is common. The background pixels are filled with the value 0, while any isolated holes inside a cell candidate region are still coded as 255. Afterwards, such pixels are changed to 1 to fill the holes.

H) **Cutting** apart cells that were not properly separated by the above algorithms may be necessary, particularly in images where the cells are very dense. We tried to use the common technique of watershed by flooding for this purpose. That turned out, however, to split real cells into two regions wherever the Gaussian smoothing was not strong enough to give only one local minimum for each cell. Since cells often have several dark spots in their nuclei, even the smoothed image may contain several local minima per cell. We therefore developed an alternative algorithm that could cut cell candidate regions at narrow passages where the edge is concave. Such cutting would optimally separate two or more merged cells where they touch. The algorithm is used on all cell candidate regions which have an equivalent circular diameter larger than the minimum cell diameter set by the user. The edge pixels of these regions are identified, and the coordinates stored in an ordered list, beginning and ending with the lower left pixel and moving clockwise around the periphery of the candidate region. From this list of points, a two-dimensional matrix is generated which contains the distances between any two pairs of edge pixels. A local minimum in this matrix, which does not lie near the matrix diagonal, will reveal two separated edge pixels that represent a narrow passage where two cells have merged. However, two opposite edge pixels on a narrow cell with nearly parallel edges will also give a local minimum in the distance matrix. Therefore, celltraxx.exe uses vector cross products and circle fitting to calculate the local radius of curvature at the candidate edge points. Convex edge points get a negative radius of curvature value to distinguish them from the desired concave points. We tried many different selection criteria to get cuts at the desired locations, but not across narrow "structures" on cells or over too long distances on large clusters of cells. The current criterion can certainly be further optimized, but uses a combination of several tests that require both candidate points to be concave edge points with small radii of curvature, and that the cutting distance is small compared to the user defined cutting diameter. In addition, the midpoint of the potential cut must lie inside the candidate cell region. With these criteria, celltraxx.exe can reasonably well split cells that were not properly separated during the initial segmentation. Cutting lines made by this algorithm are displayed in the ImageJ tuning window as blue lines. The user can therefore fine tune the cutting diameter value and instantly see if this improves the cell separation. The cutting algorithm was developed independently by us, but its initial stages are somewhat similar to the method recently proposed by Miró-Nicolau[2]. The type of cutting between concave edge points is also described by Wang in 2016 and Zhang in 2022[3, 4].

I) **Saving the tuning image** to file is only done when celltraxx.exe is run from the ImageJ macro in tuning mode. Then celltraxx.exe stops execution to allow the user to inspect how the changed settings affected the segmentation of the image. The ImageJ macro also allows the user to select the middle or last image of the video. When one of these buttons are clicked, celltraxx.exe runs in tuning mode but analyses the selected image instead of the first one.

J) **Tracking** or **matching of identified cells** with the corresponding cells in the previous image is done by using the nearest neighbour method for the identified cell centroids. A two-dimensional matrix is populated with the distances between each cell in the current image and each cell in the previous image. The shortest distance in the entire matrix is located and taken as the currently most certain match of a cell from one image to the next. Once a cell has been matched, the corresponding row and column in the distance matrix are filled with a high value to prevent the cell from being matched again, and to give a new global minimum distance somewhere else in the matrix. This process is repeated until the user defined maximum cell jump distance (= *Highest cell velocity * Time between images * Image number increment*) is reached. In this way, the stationary cells are matched first, giving a reasonably robust matching scheme. Our method was programmed independently, but turned out to be quite similar to the algorithm used by for instance Yang et al., 2020[5]. Any cells in the current image that are not matched by a cell in the previous image are kept in memory so they may be matched with cells in the next image and thus become the start of a new track. Since celltraxx.exe is mainly intended to measure the cell velocity and not to produce a high-quality map of cell lineages and track splitting, the program currently does not detect cell division events and simply lets the track continue on the nearest daughter cell, starting a new track on the other daughter cell. There is, however, a simple track repair function which checks if a non-matched cell can be matched with another non-matched cell two time steps earlier. If so, a new cell is added to the list of cells in the intermediate image by placing the cell midway between the identified cells.

K) **Smoothing of tracks** is an option in celltraxx.exe which was introduced to get more correct average velocities for stationary cells versus moving cells. Since the dark spots in the cell interior will often move around even if the cell as a whole lies quite still, the centroid of the identified cell region will typically have small sideways movements also for stationary cells. This makes the track look like a tangle of short line segments for such cells. Since the average velocity of a cell is equivalent to the total length of the track divided by the duration of the track, a stationary cell can still get an appreciable average velocity even though it has not moved a detectable net distance sideways. The Euclidian velocity, calculated as the distance from start to end of the track divided by its duration will then be a better measure of the cell's motion. To mitigate this problem, celltraxx.exe can optionally run a simple, gliding average filter on the x and y coordinates of all tracks. The filter smooths each track over time by giving a weight of 0.25 to the previous time step, 0.5 to the current time step and 0.25 to the next time step. This weighting scheme will turn a regular zig-zag function into a flat curve midway between the alternating max and min values. The first and last points of the tracks are left unchanged by the smoothing filter. By running this simple filter over several iterations, the tracks can be smoothed to the desired degree.

L) **Results files** in CSV format which summarize the analysis of each video are written to the current results directory when the last image of each video has been processed. When all available videos have been analysed, celltraxx.exe also finishes the before mentioned file "_Grey_level_curves_for_first_images.csv" along with three other summary CSV files: "_Identified_cells_summary.csv" allows easy plotting of the number of cells as a function of image number and time. "_Log_window_summary.csv" contains the information tabulated in the command window when celltraxx.exe is running, thus includes a log of how far each image had to be shifted laterally if the image shift correction was activated. "_Overall_summary.csv" lists relevant statistics for each video, including the mean

value, standard deviation, and standard error of the mean for cell velocities and several common tracking metrics.

M) **Bitmap images** from the various stages of the analysis are written to the results directory if the user has activated this option. Most images are written during the analysis of each image. However, the images which contain valid tracks cannot be generated until the entire video has been analysed and celltraxx.exe has found the tracks which are of sufficient length. Therefore, celltraxx.exe always writes the shifted images to the results folder. When the analysis is finished and bitmaps for the video with valid tracks shall be generated, celltraxx.exe reads back the shifted images in turn and paints the coloured tracking dots at the centre of mass and the tracking tails on each image. Unless the user has decided to keep the shifted images, celltraxx.exe deletes them after use. Similarly, the ImageJ macro cleans up the results folder by deleting the valid track bitmaps after the AVI video has been generated.

References

1. Caruana, RA *et al.* Fast algorithm for the resolution of spectra. *Anal Chem* **58** (1986).

2. Miró-Nicolau, M *et al.* Improving concave point detection to better segment overlapped objects in images. *Multimedia Tools and Applications* (2023).

3. Wang, W *et al.* (ed. R. Sudhakar) (IntechOpen, Rijeka; 2016).

4. Zhang, Z *et al.* A novel concavity based method for automatic segmentation of touching cells in microfluidic chips. *Expert Systems with Applications* **202** (2022).

5. Yang, FW *et al.* Investigating Optimal Time Step Intervals of Imaging for Data Quality through a Novel Fully-Automated Cell Tracking Approach. *Journal of Imaging* **6** (2020).
