# Supplementary material for: Automated tracking of cell migration in phase contrast images with CellTraxx
Source: Sci Rep. 2023 Dec 27;13:22982. doi: 10.1038/s41598-023-50227-9 (PMC10752880; doi:10.1038/s41598-023-50227-9)
Supplement: Supplementary file 22 — Supplementary Information 3. [file 41598_2023_50227_MOESM22_ESM.pdf]

```
Wound healing mode                no
Perform flat field correction     no
Perform image shift correction    yes
Perform interactive tuning        no
Version                           4.6
Track smoothing iterations        0
Results folder drive letter       C
Results folder name               1
_Random_migration_HeLa_cells_GFR=20um_d=16-48um_cut=19um
First part of folder name         1_Random_migration_HeLa_cells
Pixel size [um]                   1.24
Gaussian filter radius [um]       20
Smallest cell diameter [um]       16
Largest  cell diameter [um]       48
Cutting  cell diameter [um]       19
Top    crop margin [pixels]       20
Bottom crop margin [pixels]       20
Left   crop margin [pixels]       20
Right  crop margin [pixels]       20
Time between images      [min]    10
Highest cell velocity [um/min]    4
Shortest cell track   [images]    20
First image number                0
Last image number                 84
Image number increment            1
Make identified cell videos       no
Make matched cell videos          no
Make valid track videos           yes
Tracking dot diameter [um]        9
Valid track image contrast        2
Scale bar color                   white
Draw cell outline                 yes
Draw cell track line              yes
Write mirror margin images        no
Write shifted images              no
Write gaussian smoothed images    no
Write segmented cell images       no
Write cut cell images             no
Write identified cell images      no
Write matched cell images         no
Write valid track images          no
Keep cells from previous image    yes
Segmentation limit [SDs]          1.5
# bins in histogram               100
Tuning image code                 0
```
