# Supplementary material for: Automated tracking of cell migration in phase contrast images with CellTraxx
Source: Sci Rep. 2023 Dec 27;13:22982. doi: 10.1038/s41598-023-50227-9 (PMC10752880; doi:10.1038/s41598-023-50227-9)
Supplement: Supplementary file 23 — Supplementary Information 4. [file 41598_2023_50227_MOESM23_ESM.pdf]

Comparing CellTraxx results to gold truth cell positions from the Cell Tracking Challenge

In another attempt to test how accurate CellTraxx is tracking, we used CellTraxx to analyse a movie from the Cell Tracking Challenge repository (http://data.celltrackingchallenge.net/training-datasets/BF-C2DL-MuSC.zip). The Cell Tracking Challenge is an initiative to promote the development of cell tracking tools and offers a standardized, objective evaluation of these tools (http://celltrackingchallenge.net/). Their benchmarking parameter for segmentation (SEG) is based on reference images with manually drawn areas of selected cells. Since CellTraxx does not attempt to find the full outlines of cells, we could not run a fair test using the SEG parameter. Further, the tracking evaluation parameter (TRA) is based on lineage tracking, while CellTraxx is not reporting this type of data. We could therefore not use the Cell Tracking Challenge metrics SEG or TRA to investigate how well CellTraxx works. However, as a compromise, we used TrackMate in ImageJ to track the given gold truth images from the Cell Tracking Challenge repository. This gave a series of tracks with the (X,Y) centre positions which we could compare to the coordinates from our CellTraxx analysis of the actual, live cell images. We chose to test our tracking on an image series of mouse muscle stem cells in hydrogel microwells ("BF-C2DL-MuSC") since the cell size and distribution somewhat resembled the images that CellTraxx was developed for. We analysed the hundred last images of the series since they had the most cells and would give a good test of CellTraxx without having to handle too many images. To process these images in CellTraxx, we had to invert the images since the original cell centres were brighter than the surroundings. Due to quite uneven background grey level we also had to apply CellTraxx' pseudo flat-field correction and use a fixed segmentation limit (see Supplementary file S1 for a more detailed description of these features). With these adaptions, we found that the (X,Y) positions of the trajectories from CellTraxx matched very well with the gold truth cell coordinates (Figure S3 below).

To calculate the distance between CellTraxx and the gold truth, we allowed matching of several different, short CellTraxx trajectories into each of the gold truth tracks. Only CellTraxx tracks where the starting points were less than 15 µm from a track point in the gold truth data were included, a total of 121 tracks. The mode (most common) point distance between CellTraxx and the gold truth was 2.3 µm, while 14 % of the points missed by more than 10 µm (Figure S3b).

Although CellTraxx managed to detect the location of the real cell centres with high precision, our program was not particularly good at tracking the movement of the cells in these videos over long times. The tracks generated by CellTraxx were chopped into shorter tracks. While the gold truth data obtained by TrackMate only had 29 tracks, CellTraxx generated 183 tracks. We noticed that the cells in the video moved by extending dark protrusions which were often recognized as cells by CellTraxx (Movie 5). When the cell nucleus moved in the direction of the protrusion in the next image, the old track was lost and the new track followed. In addition, the background grey values in the image series, was dropping over time. Since CellTraxx calculates the segmentation grey level limit based on the first image, this version of CellTraxx will not work very well on videos that change grey level over time. We propose that CellTraxx works best on videos imaged with Incucyte S3 or similar microscopes which generate image series with a steady grey level background.

Despite the challenges mentioned above, the velocities based on the tracking performed by CellTraxx on the real cell video and TrackMate on the gold truth video were quite similar (Figure S3c-e). When we plot the velocity versus time (Figure S3d) or the mean velocities of each time step (Figure S3e), we clearly see a good correspondence. Velocity mean values were 1.9 µm/min for CellTraxx and 2.0 µm/min for the gold truth data tracked by TrackMate. The movie contains only 15-25 cells. This gives poor counting statistics and thereby, noisy data (high and low peaks in Figure S3d and wide spread in Figure S3e). However, the fluctuations in Figure S3d match very well between the CellTraxx analysis of live cells and the TrackMate analysis of gold truth images. The good correspondence in the velocity measurements is not surprising since CellTraxx could find the cell centres with high precision even though the tracks were often short.
