# Supplementary material for: Automated tracking of cell migration in phase contrast images with CellTraxx
Source: Sci Rep. 2023 Dec 27;13:22982. doi: 10.1038/s41598-023-50227-9 (PMC10752880; doi:10.1038/s41598-023-50227-9)
Supplement: Supplementary file 24 — Supplementary Legends. [file 41598_2023_50227_MOESM24_ESM.docx]

**Movies:**

Movie 1. **Tracking of dividing cells by CellTraxx*.*** The example presented is a zoom-in taken from Movie 3, showing migrating HeLa cells captured in Incucyte S3 every 10 minutes and then analysed by CellTraxx. Note the dividing cell in the middle (marked with green) and the dividing cell at the left border (marked in red). Scale bar 50 µm.

Movie 2. **HeLa cells identified by CellTraxx.** Migrating HeLa cells captured every 10 minutes for 14 hours in Incucyte S3 and then analysed by CellTraxx. The movie shows identified cells in each image outlined in nuances of green. Scale bar 100 µm.

Movie 3. **Tracking of HeLa cells by CellTraxx.** Migrating HeLa cells captured every 10 minutes for 14 hours in Incucyte S3 and then analysed by CellTraxx. The movie shows valid tracks in each image over time. Scale bar 100 µm.

Movie 4. **Tracking of HeLa cells by CP+TM.** Migrating HeLa cells captured every 10 minutes for 14 hours in Incucyte S3 and analysed in ImageJ/Fiji using TrackMate in combination with the integrated Cellpose detector. The movie shows valid tracks in each image over time. Note that this video is based on the same data as for Movie 2 and 3, but was cropped before the analysis to match the crop margins of Movie 2 and 3.

Movie 5. **Tracking by CellTraxx of Cell Tracking Challenge video.** Images number t1275-t1375 from the last part of the training dataset BF-C2DL-MuSC 01 in the Cell Tracking Challenge repository, analysed by CellTraxx. The movie shows valid tracks in each image over time. Scale bar 100 µm.

Movie 6. **Tracking of RPE1 cells by CellTraxx.** Migrating RPE1 cells captured every 10 minutes for 10 hours in Incucyte S3 and then analysed by CellTraxx. The movie shows valid tracks in each image. Scale bar 100 µm.

Movie 7. **Tracking of MDA-MB-231 cells by CellTraxx.** Migrating MDA-MB-231 cells captured every 10 minutes for 10 hours in Incucyte S3 and then analysed by CellTraxx. The movie shows valid tracks in each image. Scale bar 100 µm. Note that flat-field correction was used for this analysis.

Movie 8. **Tracking of HT1080 cells by CellTraxx.** Migrating HT1080 cells captured every 10 minutes for 10 hours in Incucyte S3 and then analysed by CellTraxx. The movie shows valid tracks in each image. Scale bar 100 µm.

Movie 9. **Tracking of U2OS cells by CellTraxx.** Migrating U2OS cells captured every 10 minutes for 10 hours in Incucyte S3 and then analysed by CellTraxx. The movie shows valid tracks in each image. Scale bar 100 µm.

Movie 10. **Tracking of PC-3 cells by CellTraxx.** Migrating PC-3 cells captured every 10 minutes for 10 hours in Incucyte S3 and then analysed by CellTraxx. The movie shows valid tracks in each image. Scale bar 100 µm. Note that flat-field correction was used for this analysis.

Movie 11. **Tracking of PC-3 cells by CellTraxx without flat-field correction.** Migrating PC-3 cells captured every 10 minutes for 10 hours in Incucyte S3 and then analysed by CellTraxx. The movie shows valid tracks in each image. Scale bar 100 µm. Note that this is the same video as presented in Movie 10 but here analysed without flat-field correction.

Movie 12. **Tracking by CellTraxx of MDA-MB-231 cells embedded in collagen.** MDA-MB-231 cells migrating inside collagen captured every 10 minutes for 18 hours. The movie shows valid tracks in each image. Scale bar 100 µm.

Movie 13. **Tracking by CellTraxx of HT1080 cells in a wound healing experiment.** HT1080 cells migrating in a wound healing experiment were imaged every 5 minutes for 15 hours. The movie shows valid tracks in each image. Scale bar 100 µm.

Movie 14. **Tracking by CP+TM of HT1080 cells in a wound healing experiment.** HT1080 cells migrating in a wound healing experiment were imaged every 5 minutes for 15 hours and analysed in ImageJ/Fiji using TrackMate in combination with the integrated Cellpose detector. The movie shows valid tracks in each image for the 45 longest tracks recorded.

Movie 15. **Tracking by CellTraxx of PC-3 cells without HGF stimulation.** Migrating PC-3 cells without HGF stimulation captured every 10 minutes for 10 hours in Incucyte S3 and then analysed by CellTraxx. The movie shows valid tracks in each image. One representative replicate is shown. Scale bar 100 µm.

Movie 16. **Tracking by CellTraxx of PC-3 cells with HGF stimulation.** Migrating PC-3 cells with HGF stimulation captured every 10 minutes for 10 hours in Incucyte S3 and then analysed by CellTraxx. The movie shows valid tracks in each image. One representative replicate is shown. Scale bar 100 µm.

Movie 17. **Tracking by CellTraxx of PC-3 cells without HGF stimulation in a wound healing experiment.** A wound healing experiment where unstimulated PC-3 cells were imaged every 5 minutes for 15 hours in Incucyte S3 and then analysed by CellTraxx. The movie shows valid tracks in each image. One representative replicate is shown. Scale bar 100 µm.

Movie 18. **Tracking by CellTraxx of PC-3 cells without HGF stimulation in a wound healing experiment.** A wound healing experiment where HGF stimulated PC-3 cells were imaged every 5 minutes for 15 hours in Incucyte S3 and then analysed by CellTraxx. The movie shows valid tracks in each image. One representative replicate is shown. Scale bar 100 µm.

Supplementary File S1. CellTraxx User Manual.

Supplementary File S2. A description of the CellTraxx algorithms.

Supplementary File S3. An overview of the settings used by CellTraxx to analyse the HeLa cell image series described in Figure 2.

Supplementary File S4. Comparing CellTraxx to gold truth cell positions from the Cell Tracking Challenge.
